# Biclustering reveals breast cancer tumour subgroups with common clinical features and improves prediction of disease recurrence

**DOI:** 10.1186/1471-2164-14-102

**Published:** 2013-02-13

**Authors:** Yi Kan Wang, Cristin G Print, Edmund J Crampin

**Affiliations:** 1 Auckland Bioengineering Institute, University of Auckland, Auckland, New Zealand; 2 Department of Molecular Medicine and Pathology, University of Auckland, Auckland, New Zealand; 3 New Zealand Bioinformatics Institute, University of Auckland, Auckland, New Zealand; 4 Maurice Wilkins Centre for Molecular Biodiscovery, University of Auckland, Auckland, New Zealand; 5 Department of Engineering Science, University of Auckland, Auckland, New Zealand; 6 Melbourne School of Engineering, University of Melbourne, Victoria, Australia

**Keywords:** Biclustering, Gene expression profiles, Tumour classification, Survival prediction, Breast cancer

## Abstract

**Background:**

Many studies have revealed correlations between breast tumour phenotypes, variations in gene expression, and patient survival outcomes. The molecular heterogeneity between breast tumours revealed by these studies has allowed prediction of prognosis and has underpinned stratified therapy, where groups of patients with particular tumour types receive specific treatments. The molecular tests used to predict prognosis and stratify treatment usually utilise fixed sets of genomic biomarkers, with the same biomarker sets being used to test all patients. In this paper we suggest that instead of fixed sets of genomic biomarkers, it may be more effective to use a stratified biomarker approach, where optimal biomarker sets are automatically chosen for particular patient groups, analogous to the choice of optimal treatments for groups of similar patients in stratified therapy. We illustrate the effectiveness of a biclustering approach to select optimal gene sets for determining the prognosis of specific strata of patients, based on potentially overlapping, non-discrete molecular characteristics of tumours.

**Results:**

Biclustering identified tightly co-expressed gene sets in the tumours of restricted subgroups of breast cancer patients. The co-expressed genes in these biclusters were significantly enriched for particular biological annotations and gene regulatory modules associated with breast cancer biology. Tumours identified within the same bicluster were more likely to present with similar clinical features. Bicluster membership combined with clinical information could predict patient prognosis in conditional inference tree and ridge regression class prediction models.

**Conclusions:**

The increasing clinical use of genomic profiling demands identification of more effective methods to segregate patients into prognostic and treatment groups. We have shown that biclustering can be used to select optimal gene sets for determining the prognosis of specific strata of patients.

## Background

Breast tumours are currently primarily classified based on histological appearance or grade, as well as using certain molecular biomarkers. Numerous studies have correlated variation in gene expression between individuals to phenotypic diversity in breast tumours [1-8]. Information about expression of biologically important genes may improve clinical decision-making by improving the prediction of clinical outcomes, in comparison to traditional histological grade [7,9-14]. Perou et al. [2] pioneered breast tumour classification based on hierarchical clustering of genes, identifying biologically meaningful molecular signatures associated with features such as cellular proliferation and signalling pathway activity. Related work [3,15,16] has identified five distinct molecular-based subtypes (the PAM50 subtypes): Basal, Her2, Luminal A, Luminal B and Normal-like breast tumours. A recent study has clustered a combined dataset including germline and somatic copy number variation, SNP information and gene expression data for some 2000 breast tumours, finding 10 molecularly defined subgroups with apparently distinct biology and disease-specific survival characteristics [17].

Many of these studies use clustering techniques [18-23] to group tumours with similar gene expression patterns, and thus to identify clinically relevant molecular biomarkers for tumour classification. However, genes that are co-expressed across one subset of tumours may be expressed almost independently in another subset [24]. Therefore, subtle but important biological features characterised by specific gene sets may be missed when viewed across data sets made up of a wide range of tumours.

To overcome this limitation, biclustering algorithms group genes and tumour samples simultaneously, to identify subsets of genes with similar expression patterns over subsets of tumour samples. Biclustering was first applied in gene expression profiling studies by Cheng and Church [22]. Since then various biclustering approaches have been proposed [20,21,25-32]. We reasoned that biclustering may be able to identify clinically-significant gene expression modules that stratify breast cancers according to inter-tumour heterogeneity. To our knowledge, the use of biclustering has not previously been investigated for this purpose. We used a biclustering technique, cMonkey [32], to group breast tumours from 437 patients into subgroups which were conditionally dependent on expression profiles of specific gene subsets. We subsequently determined the association of biclusters with differential survival outcomes, and examined the potential for biclusters to improve prediction of early relapse, in order to test the suitability of biclusters as stratified biomarkers that are personalised to specific subsets of breast cancer patients.

## Results

### Biclustering breast cancer gene expression data identifies tumour subgroups with similar clinical features

**Figure 1:**
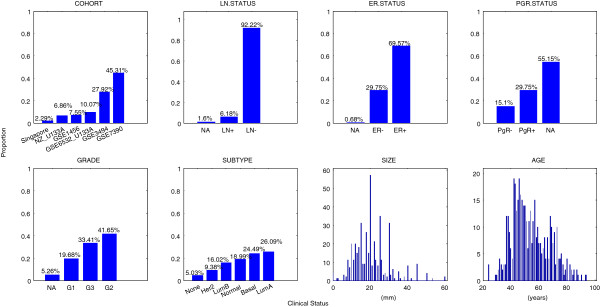
**Clinical annotations for patients without adjuvant therapy (437 patients). **The proportion of patients/tumours in each clinical category is denoted above the bars. Clinical variables include cohort, lymph node status (LN status), estrogen receptor status (ER status), Progesterone status (PgR status), tumour histological grade (G1, G2, G3), tumour subtype (Basal, Her2, LumA, LumB, Normal, None), tumour size (mm), patient age (years).

We analysed a previously published dataset which combines Affymetrix HG-U133A and HG-U133PLUS2 microarray data and matched clinical data from 6 cohorts of patients from independent studies. We selected 437 primary breast tumours from patients who had not received adjuvant or neo-adjuvant chemotherapy, endocrine-based therapy or radiotherapy (see Methods). We restricted our analysis to non-adjuvant treated tumours so that direct associations between molecular information and long-term disease outcomes might be observed without the confounding affects of different treatments. After processing, the dataset consisted of 7756 probe sets (genes) across the 437 tumours. The 7 clinical annotations for each tumour/patient were: lymph node (LN) status; hormone receptor status (estrogen receptor (ER) status and progesterone receptor (PgR) status); tumour grade; tumour subtype; tumour size; patient age. The clinical information associated with these 437 tumours is summarised in Figure 1. More than 90% of these were lymph node negative (LN-) tumours, which is consistent with the fact that a clinical decision had been made not to treat these patients with adjuvant therapy.

**Figure 2:**
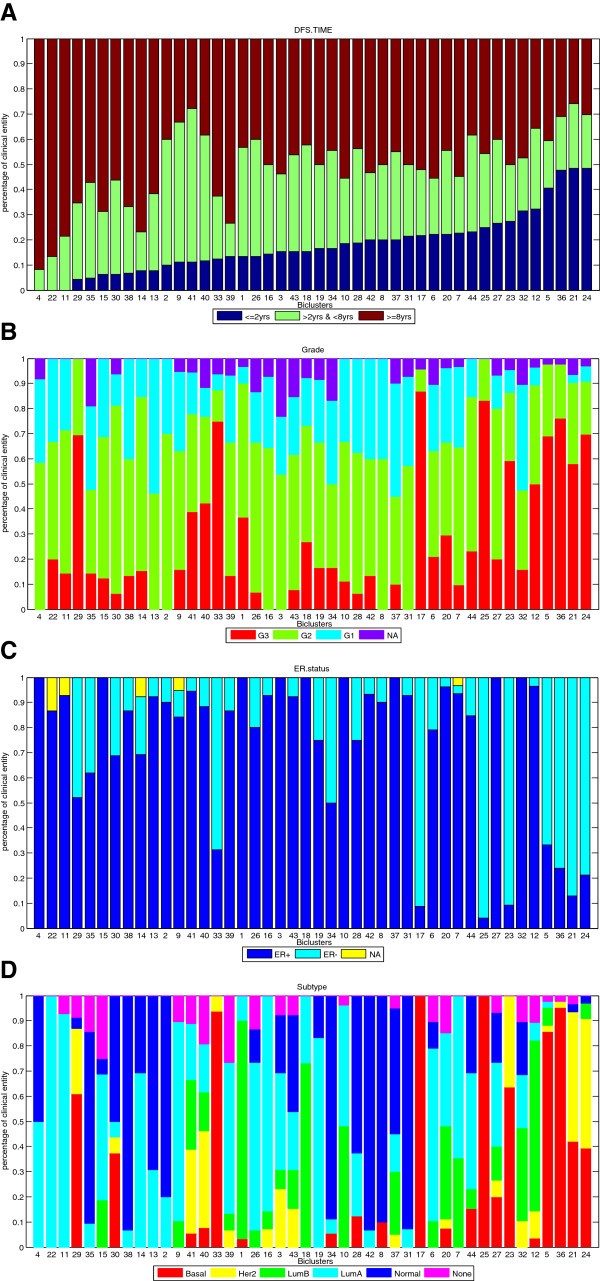
**Clinical information across all biclusters, sorted in ascending order of early relapse proportion. **Panel (**A**) Patients whose disease free survival (DFS) time was less than 2 years are classified into early relapse group (dark blue); Patients whose DFS time was more than 8 years are classified into late relapse group (dark red); and patients whose DFS time was between 2 and 8 years were grouped into the intermediate group (green). The proportion of samples in each group was computed and biclusters are ranked in ascending order of early relapse proportion. For each bicluster, the proportion of samples in each category of (**B**) Grade, (**C**) estrogen receptor (ER) status and (**D**) tumour subtype are presented.

Using cMonkey to bicluster the dataset we obtained 44 biclusters with an average number of 20 tumours contributing to each bicluster. To determine whether the tumour subgroups and corresponding gene modules identified by biclustering were biologically and clinically meaningful, we assessed whether tumours allocated to the same bicluster had similar clinico-pathological annotations and clinical outcomes, and whether the genes contributing to these biclusters were associated with the activity of distinct biological pathways. To make sure that the tumour subgroups identified in the biclusters do not simply recapitulate clinical cohort we verified that biclusters contain data derived from a range of cohorts. Then we computed the proportion of tumours in each subgroup in each of the 7 clinical categories. Figure 2 illustrates the proportion of tumour samples in each category of (A) histological grade, (B) ER status, and (C) molecular tumour subtype in each of the biclusters, ranked in order of the proportion of patients who suffered early relapse (Disease Free Survival time ≤ 2 years). These results show that biclusters do not simply recapitulate any of the already available clinical annotations.

**Figure 3:**
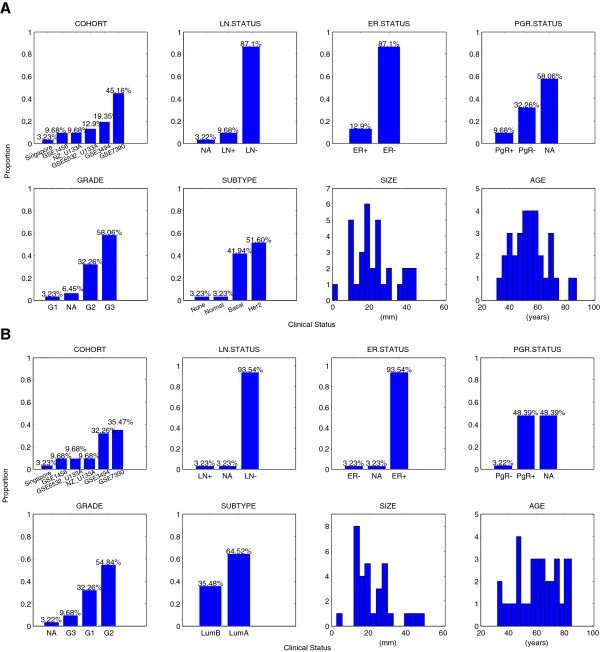
**Clinical information of tumour samples in biclusters 21 and 7. **Clinical information of tumour samples in (**A**) bicluster 21 (31 tumours) and (**B**) bicluster 7 (31 tumours), summarizing the proportion of tumours belonging to each category of clinical factor, including cohort, lymph node status (LN+, LN-), estrogen receptor status (ER+, ER-), Progesterone status (PgR+, PgR-), tumour grade (G1, G2, G3), tumour subtype (Basal, Her2, LumA, LumB, Normal, None), tumour size (mm), patient age (years). Bicluster 21 is composed of 31 tumour samples from six cohorts. y-axis indicates the proportion of samples in the group belongs to the same category. The number above each bar indicates the proportion of samples in the population of 437 non-adjuvant treated patients belongs to the same category.

Analysis of the clinical annotations of tumours in each bicluster however reveals similar clinico-pathological features. Clinical annotations for each bicluster are summarised in the supplementary material (Additional file 1: Figure S1). For example, a summary of clinical annotations for biclusters 21 and 7 are shown in Figure 3. Both consist of 31 tumour samples collected from all six cohorts. In bicluster 21 most tumours (> 90%) have either Basal-like (42%) or Her2 (52%) intrinsic subtype; most (~87%) are estrogen-receptor negative (ER-), and are grade 2 (32%) or 3 (58%). Therefore, the clinical information for bicluster 21 suggests that tumours in this group are aggressive and have pathological features associated with poor prognosis. By comparison, tumours in bicluster 7 are predominantly Luminal-like tumours, i.e. Luminal A (64%) and Luminal B (35%), and are estrogen-receptor positive (~93%), and histological grade 1 (32%) or 2 (55%). Thus the clinical information of tumours in bicluster 7 suggests that patients in this group have clinical features that are generally associated with less aggressive tumours, associated with good prognosis. Similar patterns can be observed in the remaining biclusters (see Additional file 1).

Overall, these data suggest that biclustering identifies subgroups of tumours selected from multiple cohorts, which do not simply recapitulate existing clinical annotations, but which are similar in clinico-pathological characteristics.

### Biclustered subgroups are associated with differential survival outcomes

**Figure 4:**
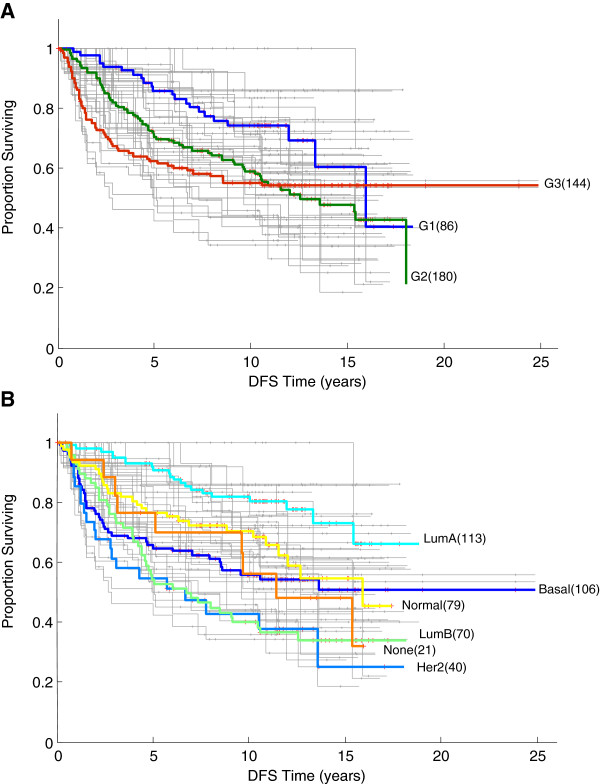
**Kaplan-Meier estimate of disease-free survival (DFS) distribution. **Kaplan-Meier plots of DFS for all biclustered patient groups (44 biclusters) in grey, superimposed on survival curve for tumours classified by histological grades (**A**) and tumour subtypes (**B**).

To test the hypothesis that subgroups of tumours identified in biclustering are associated with differential patient outcomes, we examined the disease free survival (DFS) distribution of patients in each bicluster. Figure 4 shows Kaplan-Meier survival curves for all 44 biclusters (grey) superimposed on the survival curves for histological grade (panel A) and tumour subtype (panel B). Bicluster-associated survival curves show a greater range than the grade-associated and subtype-associated survival curves. To test whether this range of survival distributions might arise by chance, we randomly assigned (sampling with replacement) the 437 tumours into 44 arbitrary groups with the same distribution of sizes as found in the biclusters. The Kaplan-Meier survival curves of these arbitrary groups (see Additional file 1: Figure S2) visually suggest that the bicluster-specific survival curves are more widely separated than expected at random. In order to assess this statistically, we computed the logrank-p values for all possible pairings of biclusters and for all possible pairings of the randomised groups. A two-sample Kolmogorov-Smirnov (KS) test rejected at the 5% significance level the null hypothesis that these distributions were the same (p-value = 1.7193 × 10^-16^). These results indicate that membership of the biclusters is associated with differential survival outcomes.

### Tumours grouped in biclusters are molecularly distinct

To reveal molecular activities of oncogenic pathways associated with the biclusters, we used a univariate Cox PH model to determine the molecular features most strongly associated with clinical outcome for each bicluster, identifying probe sets whose expression was significantly associated with DFS (Cox PH p-value < 0.01). We then used the GeneSetDB web tool [33] to identify the significantly enriched (GeneSetDB p-value < 0.01) functional annotations of these genes (see Additional file 2). For example, RNAs associated with several of the biclusters were enriched for cell cycle annotations (e.g. cell division [GO: 0051301] in bicluster 1, cell cycle checkpoint [GO: 0000075] in bicluster 10, mitotic cell cycle [GO: 0000278] in bicluster 17 and mitotic cell cycle checkpoint [GO: 0007093] in bicluster 26), proliferation (e.g. decreased cell proliferation [MP:0000352] in biclusters 18, 20 and 26, cell proliferation [GO: 0008283] and decreased B cell proliferation [MP: 0005093] in bicluster 23, increased cell proliferation [MP:0000351] in bicluster 33). RNAs associated with several of the biclusters were also enriched for immune response annotations (e.g. immunological synapse [GO: 0001772] and wound healing [GO: 0042060] in bicluster 7). Genes strongly associated with specific biclusters were also found to be significantly enriched for cancer-associated transcription factors, such as MYC (e.g. biclusters 35 and 38), STAT1 (e.g. biclusters 7 and 28), E2F (e.g. V$E2F_Q3_01 and V$E2F1_Q6: E2F-1 in biclusters 25 and 35), SP1 (e.g. biclusters 30 and 33) and NFE2L2 (bicluster 23), and enriched in regulatory pathways, which play an important role in breast cancer progress, such as Regulatory DNA replication (REACTOME Pathway ID: 69304) and p53-Dependent G1/S DNA damage checkpoint (REACTOME pathway ID: 69620 and 69613)) in bicluster 23, G1/S-Specific Transcription (REACTOME pathway ID: 453279 and 69205) in biclusters 18 and 20, Interferon signalling (REACTOME pathway ID: 913531) in biclusters 7 and 10, and SREBP signalling (Wikipathway PW:0000753) in bicluster 23. Additional file 3: Table S1 summarises biological meaningful annotations for biclusters associated with low risk of disease recurrence and for biclusters associated with high risk of relapse.

To compare the gene sets identified for each bicluster we searched for biologically significant genes which were differentially expressed between pairs of biclusters, showing a significant difference (logrank p-value < 0.01; Additional file 1: Figure S3) in survival distribution (see Methods; Additional file 1: Figure S4). As an example, Additional file 3: Table S2 summarises significant differentially expressed genes between bicluster 7 and bicluster 21. We analysed these gene classifiers and found them to be highly enriched in GO categories including response to estrogen stimulus, mammary gland development, defence response and wound healing. (A selected list of biological terms significantly enriched with differentially expressed genes between biclusters 7 and 21 is summarised in Additional file 3: Table S3). The gene classifiers were also highly associated with transcription factor STAT group (TransFac, p-value < 0.001), and were significantly enriched in regulatory pathways such as Downregulated of MTA-3 in ER-negative Breast Tumors (Biocarta, p-value = 1.80 × 10^-4^) and Role of ERBB2 in Signal Transduction and Oncology (Biocarta, p-value = 2.50 × 10^-3^). Breast abscess (Disease/Phenotype, p-value = 5.90 × 10^-6^) and abnormal mammary gland alveolus morphology (Disease/Phenotype, p-value = 7.90 × 10^-5^) were also found in genes characterizing the difference in survival outcomes between these two biclusters.

Overall, these results imply that the biclusters are molecularly defined by functionally coherent, but distinct, gene expression modules.

### Bicluster gene classifiers are different to commercial Oncotype DX and MammaPrint profiles

**Figure 5:**
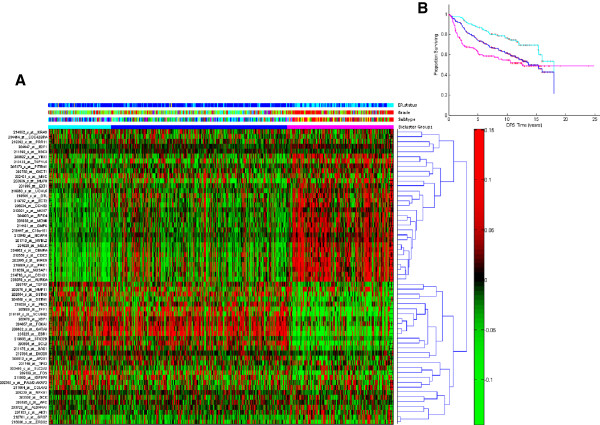
**Gene expression for tumours in each bicluster of known biomarkers. **Panel (**A**): Heatmap of oncogenic pathway activity across biclusters, sorted by the proportion of tumour samples in early relapse (DFS ≤ 2 years), as for Figure 2 (A). The colour bar labelling is the same as used in Figure 2. Each row represents a gene signature and each column represents a tumour. The red colour indicates that the expression of gene is higher than the average expression across all tumours; the green colour indicates that the expression of gene is lower than the average expression across all tumours. Panel (**B**): Kaplan Meier (KM) plots for survival estimation of patients in first 10 biclusters (KM curve in cyan), last 10 biclusters (KM curve in magenta) and the remainder of the biclusters (KM curve in blue).

To determine whether currently known biomarkers are associated with the biclusters, in Figure 5 (A) we plot gene expression for tumours in each bicluster of genes from the commercialised Oncotype DX and MammaPrint diagnostic tests, the genetic grade signature proposed by Ivshina et al. [34], and other key oncogenic markers. The figure indicates three broad groupings of biclusters, which have significant differences in disease-free survival, shown in Figure 5 (B). The first 10 biclusters (cyan bar) are associated with low risk of disease recurrence (longest DFS). The last 10 biclusters (magenta bar) are associated with high risk of disease recurrence (shortest DFS). The heatmap suggests that these known biomarkers are not able to distinguish patient subgroups identified by biclustering.

We then looked at the genes and related pathways identified as being differentially expressed between biclusters associated with low and high survival prognosis. Interestingly, many of these gene classifiers are not found in currently available prognostic gene classifiers for breast cancer. For each pair of biclusters showing differentially survival outcomes, we used prediction analysis of microarrays (PAM) [35] to identify those of probe sets which were able to accurately characterise survival outcome differences between the biclusters (Additional file 4). Among the gene classifiers for bicluster 7 and bicluster 21 (see Additional file 3: Table S4), GRB7 and ERBB2 (highly expressed in bicluster 21) and ESR1 and BCL2 (highly expressed in bicluster 7) are members of the Oncotype DX gene list. Overall, however, we found only a small proportion of gene classifiers that coincide with the commercial gene lists.

### Biclusters show strong prognostic association with breast cancer patient survival in comparison to conventional clinical markers

We used a univariate Cox PH model to examine the association between conventional prognostic factors (including hormone receptor status, lymph node status, tumour size and age) and disease free survival (DFS) time. Clinical variables were entered as binary data while tumour size and age were discretized into categorical variables (see Methods). The strength of association between clinical variables and the survival outcome, as quantified by Cox PH p-value, was compared to prognostic significance of biclustered tumour groups, using the same univariate Cox PH model. Bicluster membership was found to have a strong association with DFS (univariate Cox PH p = 8.67 × 10^-5^), after LN status (p = 1.71 × 10^-5^) and tumour size (p = 7.44 × 10^-5^), shown in Additional file 3: Table S5 (a). We also found that biclusters give better prognostication of relapse compared to the conventional molecular tumour subtype (univariate Cox PH p = 0.000427).

Next, we used a multivariate Cox PH model to estimate the association of biclusters with DFS in combination with the conventional clinical factors. We found that biclusters were significantly statistically associated with DFS time (Additional file 3: Table S5 (a), multivariate Cox PH p = 0.00103; compared to randomly allocated groups, Additional file 3: Table S5 (f), multivariate Cox PH p = 0.6163).

Finally, we also found that biclusters show stronger prognostic association with disease recurrence within pathology-specific tumour subsets (Additional file 3: Table S5 (b)-(e): LN- patient subgroup, univariate Cox PH p = 0.000183; ER+ patient subgroup, p = 0.00406; Grade 2 tumour subgroup, p = 0.000171; Grade 3 tumour subgroup, p = 0.0153). In each of these pathology-specific tumour subsets, multivariate Cox PH analysis also demonstrated that the tumour biclusters were significantly in associated with DFS compared to molecular tumour subtype when combined with other conventional clinical variables (Additional file 3: Table S5 (b)-(e)).

These univariate and multivariate Cox PH analyses suggest that the classification of tumours using biclustering can, alone or in combination with other clinical factors, be a significant prognostic variable, especially in pathology-specific patient subsets, in comparison to conventional clinical-pathological factors.

### Biclusters increase prediction of patient survival

The ultimate goal of tumour classification is to improve prediction of an individual’s risk of relapse, so as to aid the clinician in decision making on treatment strategy (e.g. whether a patient should or should not be given adjuvant therapy). To test whether biclusters improve prediction of patient survival, we used a conditional inference tree model to predict disease recurrence. We chose to examine disease recurrence (relapse) within 2 years of primary surgery. Therefore, we defined a dichotomous clinical outcomes decision tree, in which early relapse equates to disease free survival time ≤ 2 years, and late relapse to DFS time ≥ 8 years (defined in this manner the data set contains 92 early relapse and 211 late relapse patients). Bicluster membership (representing molecular information) and clinical information including hormone receptor status (ER status), lymph node status, histological grade, tumour size and patient age were included as binary variables for each tumour.

**Figure 6:**
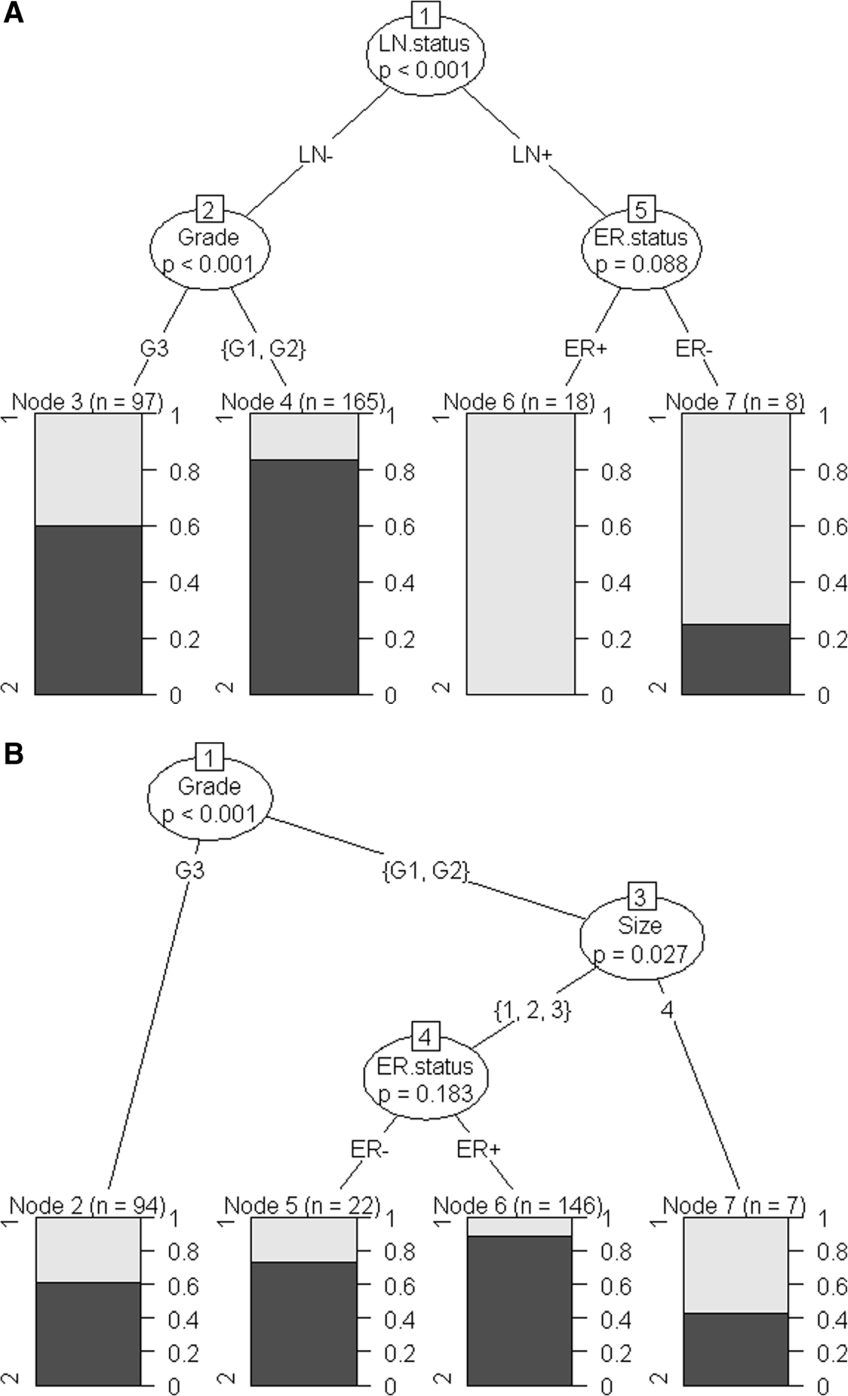
**The schematic diagram of the conditional inference tree of model using only conventional clinical variables. **Panel (**A**) indicates that Lymph Node status is the most significant factor followed by Grade and ER status. The survival outcomes are displayed in bar graphs in which Group1 represents the proportion of patients with DFS time < 2 yrs (early relapse) and Group2 represents the proportion of patients with DFS time > 8 yrs (late relapse). The tree is trimmed at the significance level of 0.5. Panel (**B**) illustrates a decision tree constructed for LN- tumours only.

**Figure 7:**
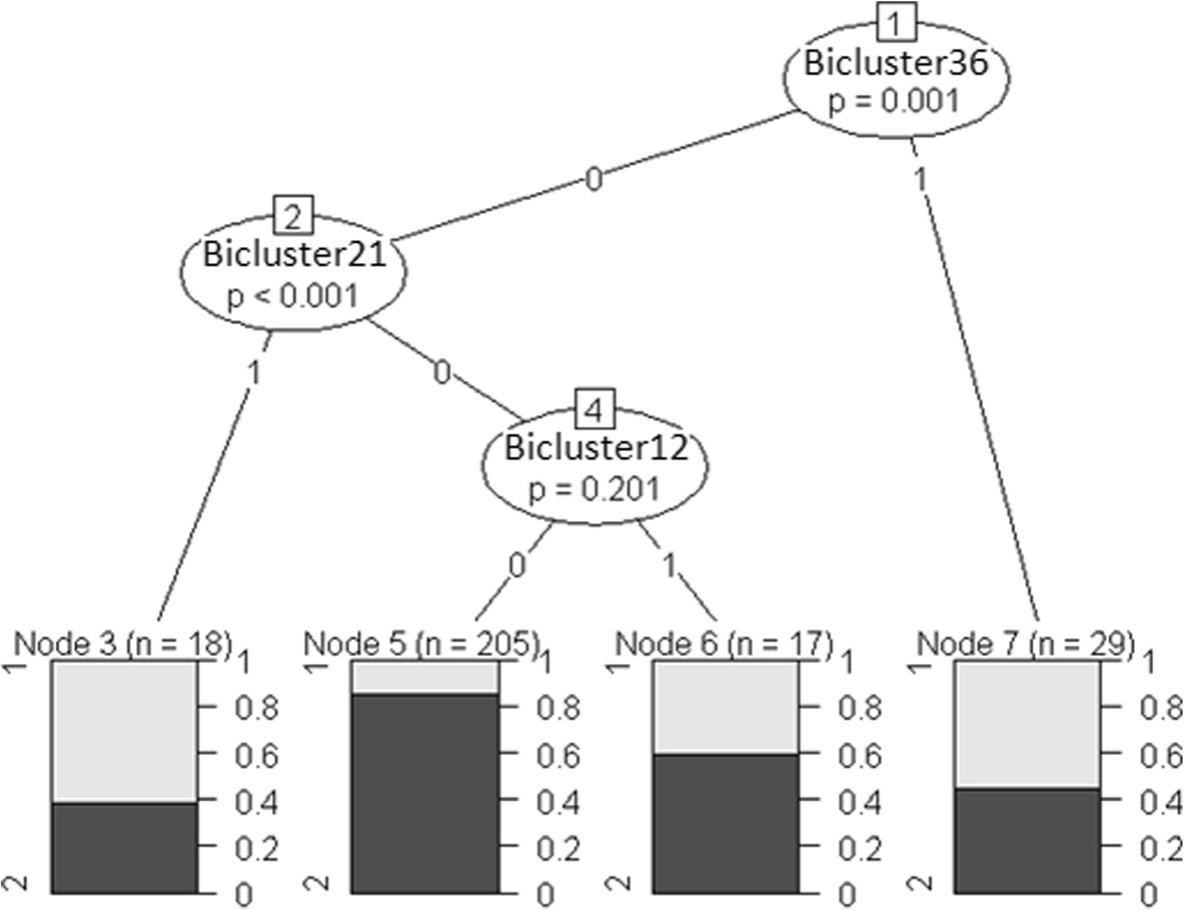
**The schematic diagram of the conditional inference tree constructed for LN- tumours incorporating bicluster membership. **The survival outcomes are displayed in bar graphs in which Group1 represents the proportion of patients with DFS time < 2 yrs (early relapse) and Group2 represents the proportion of patients with DFS time > 8 yrs (late relapse). The tree is trimmed at the significance level of 0.5.

Initially we generated a decision tree based on conventional clinical covariates only. Figure 6 (A) demonstrates that lymph node (LN) status was found to be the most important clinical variable for estimation of patient clinical outcomes (p < 0.001) in comparison to the remaining clinical variables, followed by Tumour Grade (p < 0.001) and ER status (p = 0.088). Since ~90% of the tumours in our dataset are LN negative tumours, we constructed a decision tree for the LN− branch, shown in Figure 6 (B). In LN− tumours, histological grade was the most significant variable (p < 0.001), followed by tumour size (p = 0.027) and ER status (p = 0.183). Next we constructed a decision tree for LN− tumours using both biclusters and clinical variables. Figure 7 shows that the bicluster 36 (p = 0.001) and bicluster 21 (p < 0.001) were significant molecular covariates in LN− tumours compared to the other clinical variables. We determined similar decision trees for continuous DFS time without dichotomisation (data not shown).

To assess the performance of the conditional inference tree predictors, we performed a cross-validation by generating 50 random splits of the data into training (80%) and test (20%) sets, ensuring that both training and test sets contained similar proportion of early and late relapse patients as the full data set. Biclusters derived by applying cMonkey to the training data only were combined with clinical variables and used to generate the decision tree. Performance was assessed by comparing relapse time predicted by the decision tree to the actual survival outcomes of patients in the test set. Tumours in the test set were assigned to biclusters by calculating correlation with the first principal component of the gene expression profile of each bicluster (see Methods). To assess the contribution of biclusters to prediction of relapse times, we compared these results with relapse times predicted using decision trees constructed from clinical variables only. The average rate of correctly predicted early relapse increased from 41% to 58% by incorporating biclusters into the predictors.

To assess the performance of the predictors for a clinically-determined subset of tumours we estimated of early relapse for patients with moderately differentiated grade 2 and poorly differentiated grade 3 tumours. We found 17% and 20% improvements in prediction of early relapse in these patients by including bicluster information (increase from 39% to 56% for patients with grade 2 tumours and from 46% to 66% for patients with grade 3 tumours).

**Figure 8:**
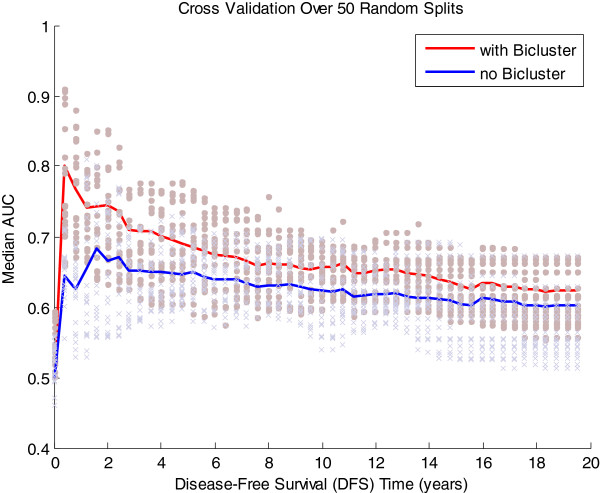
AUC evaluating the survival prediction of Cox PH based Ridge Regression Model, determined using cross validation.

We note that the conditional inference tree prediction model performed consistently with different choice of survival time boundaries (data not shown): no significant deviation in the performance was observed when different cut-off times for relapse were chosen. We also considered an alternative prediction model. Bøvelstad et al. [36,37] performed a comparative study of six Cox regression-based survival prediction methods, including univariate selection, supervised principal component analysis, partial least squares regression, ridge regression and lasso, finding that ridge regression presented the best overall performance. We repeated the above analysis using a Cox regression-based ridge regression model. A higher median area under the ROC curve (AUC) [38] was observed, in particular for DFS time less than 2 years, across 50 random data splits when the model incorporated bicluster information (Figure 8), indicating better prediction of early relapse compared to the model with clinical covariates only, qualitatively consistent with the results from the conditional inference tree.

Taken together, these results suggest that combining conventional clinical information with bicluster membership is able to provide a better prediction of early relapse in breast cancer patients.

## Discussion

In this study we have shown biclustering to be a useful approach to identifying subgroups of tumours, based on the use of stratified biomarkers that are personalised to specific subsets of patients. Biclustering determines gene modules and related clinical features which are important in determining phenotypic and clinical outcomes in those patients, but not in others.

In particular, we have applied biclustering to a large breast cancer expression data set that includes careful clinical annotations, and have used this method to identify clusters of breast tumours conditional on common expression profiles across a set of genes. We also demonstrated that biclusters do not simply recapitulate any obvious single, known clinical covariate (Figure 3 and Additional file 1: Figure S1), but instead represent a group of tumours co-expressing a set of genes that are associated with similar clinical presentation and give rise to recurrence risk. We found that biclusters have strong prognostic association with patient survival outcomes, especially in pathology-specific tumour subsets, compared to conventional molecular tumour subtype. The predictive value of biclustering in the prediction of early relapse of disease was observed by comparing prediction of conditional inference tree models constructed from clinical covariates with those constructed from a combination of clinical covariates and biclusters. On average we found that including biclusters provided close to a 20% improvement over prediction of early relapse using clinical covariates only.

We identified 44 biclusters in the dataset studied here. Comparisons showed that the tumours comprising some of these biclusters were enriched for similar clinical features, showing no significant difference in their Kaplan-Meier survival curves, despite subtle differences in the gene signatures contributing to the biclusters, i.e. *different* molecular signatures contributing to *similar* phenotype or disease state. Such differences may provide important information for prognosis and may contribute to our understanding of genetic regulatory mechanisms underlying patient outcomes.

Our analysis suggests that unsupervised biclustering is able to discover tumour subgroups determined from biologically meaningful gene sets. However, overall we found only a small proportion of these gene classifiers coincide with the 21-mRNA Oncotype DX RT-PCR assay [39] and the 70-mRNA MammaPrint [40] gene lists, which have been developed for determining the risk of breast tumour recurrence in patients with stage I or II node-negative breast cancer. By examining the expression pattern of Oncotype DX and Mammaprint profiles, as well as genetic grade markers proposed by Ivshina et al. [34], across all biclusters, we found that the expression patterns of these gene markers were associated with two broad groups of patients with differential clinical outcomes (see Figure 5 (A)). Membership of these groups is much broader than that of the biclusters we have identified. Given the functional enrichment analyses of the bicluster gene classifiers showing strong biological significance in breast cancer, this suggests a potential avenue for identifying new mRNA-based profiles for predicting disease recurrence.

Biclusters may overlap one another, both in the tumours and the gene signatures which determine them. The clinical and molecular heterogeneity within breast cancer suggests that it is unlikely that one may accurately determine unique characteristics of tumours or predict patient clinical outcomes using a single or a small group of gene markers. Different combinations of molecular pathways may be activated in, and therefore significantly associate with the recurrence of the disease in different patients. Therefore, biclusters which are determined from multiple and potentially overlapping gene expression profiles may be more relevant in the context of personalised medicine, which requires finer groupings of patients with specific molecular and clinical characteristics.

In performing cross-validation analysis we assigned ‘test’ tumours to biclusters derived from the ‘training’ set of tumours by ranking correlation with the first principal component of the gene signature of each bicluster. We found relatively high correlations between all members of the test set and all biclusters. Nevertheless, samples were allocated to biclusters with consistency in both molecular and clinical characteristics. In practice, new patients arriving in the clinic could be assigned to biclusters on the basis of both tumour gene expression and clinical covariates (we have shown that bicluster membership is associated with clinical variables, so this may add further useful information in associating new patients with tumour subgroups). Furthermore, the power of the predictive model can be improved by including new cases into the current ‘training’ population once clinical outcomes are known, hence re-determining biclusters periodically.

## Conclusions

With the increasing clinical use of genomic profiling, which is producing very large amounts of data for large numbers of patients, methods to use this data to segregate patients into prognostic and treatment groups is increasingly important. Our study proposes a biclustering approach to identify stratified biomarkers, without pre-selection of gene markers or molecular pathways. Our results show the utility of biclustering in identifying of molecularly and clinico-pathologically distinct tumour subtypes that are strongly associated with differential clinical outcomes. The prognostic significance of biclustered patient groups was observed in well-, moderately- and poorly- differentiated disease. Bicluster-specific patient stratification characterised by different gene profiles fits well within the context of personalised medicine: different sets of molecular pathways being active in different tumours.

## Methods

### Non-adjuvant treated breast cancer dataset

This dataset contains the expression profiles of more than 20,000 probe sets across 960 patient samples, measured using *Affymetrix* HG-U133A and HG-U133PLUS2 GeneChip arrays, quantile normalized across all patient samples, as described in Lasham et al. [41]. This study described gene expression microarray data and matched clinical data for primary breast tumour patients assembled from multiple published studies (GSE3494, GSE1456, GSE6532, GSE7390, GSE4922, GSE36771, and GSE36772).

In order that direct associations between molecular information and long-term disease outcomes might be observed without the confounding affects of different treatments, we restricted our analysis to patients who had not received adjuvant or neo-adjuvant chemotherapy, endocrine-based therapy or radiotherapy, resulting in a dataset for analysis containing 437 tumours from the following cohorts (Figure 1): GSE1456 (33 samples), GSE3494 (122), GSE6532_U133A (44), GSE7390 (198), NZ_U133A (30 samples; part of GSE36771) and Singapore (10 samples; 6 from GSE4922 and 4 from GSE36772).

We removed all genes without significant variation across samples by estimating a noise threshold based on the mean signal of the BioB 3’ probe set across all chips, and retaining all probes that were expressed above this noise level in at least 10% of chips. Since Affymetrix HG-U133A microarrays include multiple probe sets annotated with same official gene symbol (OGS), to avoid inclusion of outlying probe sets and to reduce computational cost, probe sets without OGS annotation in version 2.6.3 of the Bioconductor hgu133a.db annotation package were also removed. We retained 7756 probe sets for analysis – one probe set each OGS (whose expression had the highest correlation to the median expression of all probe sets with the same annotation).

Finally, to remove non-biological variation associated with cohort, ComBat [42] processing was then applied to adjust for cohort-correlated batch effect across the non-adjuvant treated tumour data set.

### Biclustering using cMonkey to identify molecular-based tumour subgroups

We used the cMonkey biclustering algorithm [32] to identify subgroups of non-adjuvant treated tumours with coherent patterns of gene expression over a subset of genes. cMonkey performs 2D clustering on both genes and conditions (microarrays) by using a Markov chain process that iteratively adds or removes genes and conditions to or from a cluster, initially with a single gene, until the optimal cluster is achieved [43]. Using this approach, genes and tumour samples can be allocated into more than one bicluster, consistent with the principle that genes may participate in more than one biological process or gene expression module [44].

We note that we used cMonkey to identify clusters of tumours conditional on common expression profiles across a set of genes, rather than the standard usage (to identify co-expressed genes across a subset of conditions), and therefore the data matrix was presented in a transposed form in comparison to normal usage. Since the genome information for *Homo sapiens* was not available for the new *R* package version of cMonkey (version 4.8.4) [45], the motif and network information was switched off in the input of the cMonkey command line.

We chose to use cMonkey because of its computational efficiency in dealing with high-dimensional microarray data, and its ability to detect co-expression patterns by considering the membership of tumours and genes simultaneously without large overlap between biclusters. However, a limitation is the relatively narrow distribution in the number of genes used to determine each bicluster – a known current limitation of cMonkey. While this biases the analysis towards biclusters defined by gene sets of similar size, the gene membership was shown to be different between biclusters.

### Differences in survival distribution – logrank test

We used the Kaplan-Meier plot to estimate disease free survival (DFS) distributions. The logrank test was used to assess the differences in survival distribution between any two sets of survival curves (smaller p-value indicates stronger statistical evidence against the null hypothesis that two curves were drawn from the same survival distribution). The Kaplan-Meier estimation and logrank test were performed using MatLab scripts *kmplot* and *logrank*[46].

### PAM to determine molecular classifiers for each bicluster

Prediction analysis of microarrays (PAM) [35] was used to determine the minimal set of genes classifying each pair of biclusters associated with low and high risk of recurrence, with the lowest misclassification rate. Initially, we ran the PAM algorithm with all 7756 probe sets as input in the low risk group to high risk group comparison and obtained a minimal set of probe sets, which gave the lowest misclassification error. All probes in the minimal set are differentially expressed between the low and high risk-associated biclusters. This was executed using the *pamr* package in R.

### Cox proportional hazards regression

The prognostic effect of molecular and clinical-pathological covariates on patient survival was assessed within the framework of Cox proportional hazard (PH) regression. A univariate Cox PH model was used to examine the individual association between each covariate and survival time. A multivariate Cox PH model was used to test the prognostic effect of combinations of covariates. The significance of association was quantified by the Cox PH p-value: smaller p-values indicate the stronger contributions of molecular and clinical variables to survival outcomes. The computation of the association between gene signatures and biclusters was implemented using the *coxphfit* function in MatLab and prognostic association was assessed using the *survival* package in R.

### Cross validation: Association of test data with biclusters

In order to determine the association between a tumour from the ‘test’ data set and the biclusters calculated from the ‘training’ set, we used the first principal component (PC1) as a representative gene expression profile for each tumour sample in each bicluster. We computed the Pearson’s correlation between these profiles and the gene expression profiles of tumours in the ‘test’ set for each bicluster. ‘Test’ tumours were associated with biclusters for which the correlation coefficient was larger than a pre-defined threshold value (> 0.9), such that each ‘test’ tumour sample could be allocated into more than one bicluster.

### Survival prediction model

To detect whether classification of tumours using biclustering can be used to improve the prediction of survival with censoring, we used two different approaches to estimate a regression relationship between survival time and the status of multiple covariates including both clinical and molecular variables (biclusters): (1) a conditional inference tree [47] constructed by recursive binary partitioning process and (2) ridge regression method based on Cox regression model [48]. For these models the covariates were entered as categorical variables, i.e. ER status (ER+, ER-); PgR status (PgR+, PgR-); LN status (LN+, LN-); Histological Tumour Grade (G1, G2, G3); Tumour Size (size < 10 mm, 10 mm ≤ size 20 mm, 20 mm ≤ size <40 mm, and size ≥40 mm); Patient Age (age < 40 years, 40 years ≤ age < 50 years, 50 years ≤ age < 60 years, and age ≥ 60 years); Bicluster Membership (0,1). The conditional inference tree was implemented through *ctree* function in the R package *party*. MatLab code for the Cox regression based ridge regression method is provided by Bøvelstad et al. [36].

The computational methods used in this study will be made available on request to the authors.

## Competing interests

The authors declare that they have no competing interests.

## Authors’ contributions

YKW performed the computational analysis and wrote the manuscript. CGP planned the project, oversaw preparation of the data set, co-supervised the computational analysis, and assisted with data interpretation and manuscript preparation. EJC planned the project, supervised the computational analysis, and assisted with data interpretation and manuscript preparation. All authors read and approved the final manuscript.

## Supplementary Material

Additional file 1: Figure S1 Clinical information for each individual bicluster determined by cMonkey from gene expression data of breast cancer patients who had not received adjuvant treatment (437 patients). The clinical variables include cohort, lymph node status (LN.status: LN+, LN- and NA), estrogen receptor status (ER.status: ER+, ER- and NA), progesterone receptor status (PgR.status: PgR+, PgR- and NA), tumour grade (Grade: G1, G2, G3 and NA), molecular subtype (Subtype: Basal, Her2, Luminal A, Luminal B, Normal and None), tumour size (Size in mm) and patient Age (in years). Missing information is noted as NA (Not Available). The percentage noted on each bar indicates the percentage of tumour samples of the specific category in each bicluster. **Figure S2. **Kaplan-Meier estimates of survival distribution of randomly selected patient groups. Kaplan-Meier estimate of disease free survival (DFS) for 44 groups with arbitrary patient membership (all patients were randomly allocated into 44 groups), superimposed on survival curve of (A) histological grades and (B) tumour subtypes, respectively. Distribution of bicluster-associated survival curves in Figure 4 is much broader than the distribution of patient group-associated survival curves by chance. **Figure S3.** Kaplan-Meier plots of pairs of biclusters showing differences in survival. Kaplan-Meier plots estimate disease free survival (DFS) distribution of pairwise biclusters comparison with significant difference in their survival distribution (logrank p value < 0.01). In each subplot, blue curve presents patients’ group associated with good prognosis while curves in green represents patients’ group associated with poor prognosis. Logrank p-values denotes on the top of each subplot indicates the significance of difference in survival distribution between two biclusters. **Figure S4. **Volcano plots showing genes that are differentially expressed between biclusters which have significant difference (logrank p values < 0.01) in their survival curves, associated with good and poor prognosis respectively. The statistical significance of differential gene expression was quantified by two sample *t*-test p-value. The fold change in expression level of each gene between two biclusters was evaluated. We identified genes whose expression level was significantly different by at least 2-fold change between biclusters associated with good and poor survival. We plot the statistical significance of differentially expressed genes against fold change in the expression levels between low and high risk biclusters to identify both statistically and biologically significant genes. By setting the cut off of significance level at 0.05 (horizontal red dashed line) and fold change at 2 (two vertical red dashed lines), the volcano plots identified genes with elevated expression levels, which represents the top-most significantly differentially expressed genes between biclusters associated with good and poor survival. The volcano plot was generated by *mavolcanoplot *in MatLab. **Figure S5. **Functional enrichment heatmap for PAM gene classifiers of biclusters which have significant difference (logrank p values < 0.01) in their survival curves, associated with good and poor prognosis. Click here for file

Additional file 2 Summarizes the significantly enriched (GeneSetDB p-value < 0.01) functional annotations of genes that were most strongly associated with clinical outcome for each bicluster. Click here for file

Additional file 3: Table S1 Functional annotation enrichment in biclusters associated with low risk of relapse and in biclusters associated with high risk of relapse. **Table S2.** Differentially expressed genes between Bicluster 7 and Bicluster 21. Genes are ranked in ascending order of p-values reported from two-sample *t*-test, and the related statistics scores of each gene, including t-scores, p-values, False Discovery Rate (FDR), q-values and Benjamini-Hochberg (BH) adjusted FDR are summarized. **Table S3. **Enrichment analysis of differentially expressed genes between Bicluster 7 and Bicluster 21 determined by GeneSetDB at the significance level < 0.001. **Table S4. **Gene Classifiers determined by PAM – the maximum number of probe sets which can accurately characterise Bicluster 7 and Bicluster 21. The highlighted genes overlap with the OncotypeDx commercial gene list. **Table S5. **Multivariate analyses of prognostic importance of biclusters in comparison to conventional clinical factors, molecular tumour subtypes, and genetic grade. Significance of biclustered classification for prognosis was compared to chance by randomly assigned tumours into 44 arbitrary groups and estimating the association between membership of these arbitrary groups and DFS. Univariate Cox PH analysis revealed no statistically significant association between arbitrary group membership and survival outcome. Significance is indicated in the ranges: 0 ≤ 0.001 ‘***’; 0.001 ≤ 0.01 ‘**’; 0.01 ≤ 0.05 ‘*’; 0.05 ≤ 0.1 ‘.’; 0.1 ≤ 1 ‘-’. **Table S6. **Summary of clinical information of 437 non-adjuvant treated patients. Click here for file

Additional file 4 Summarises probe sets which were able to accurately characterise survival outcome differences between the biclusters. Click here for file
