# Supplementary material for: Biclustering reveals breast cancer tumour subgroups with common clinical features and improves prediction of disease recurrence
Source: BMC Genomics. 2013 Feb 13;14:102. doi: 10.1186/1471-2164-14-102 (PMC3598775; doi:10.1186/1471-2164-14-102)

Supplementary Figure 1. Clinical information for each individual bicluster determined by cMonkey from gene expression data of breast cancer patients who had not received adjuvant treatment (437 patients). The clinical variables include cohort, lymph node status (LN.status: LN+, LN- and NA), estrogen receptor status (ER.status: ER+, ER- and NA), progesterone receptor status (PgR.status: PgR+, PgR- and NA), tumour grade (Grade: G1, G2, G3 and NA), molecular subtype (Subtype: Basal, Her2, Luminal A, Luminal B, Normal and None), tumour size (Size in mm) and patient Age (in years). Missing information is noted as NA (Not Available). The percentage noted on each bar indicates the percentage of tumour samples of the specific category in each bicluster.

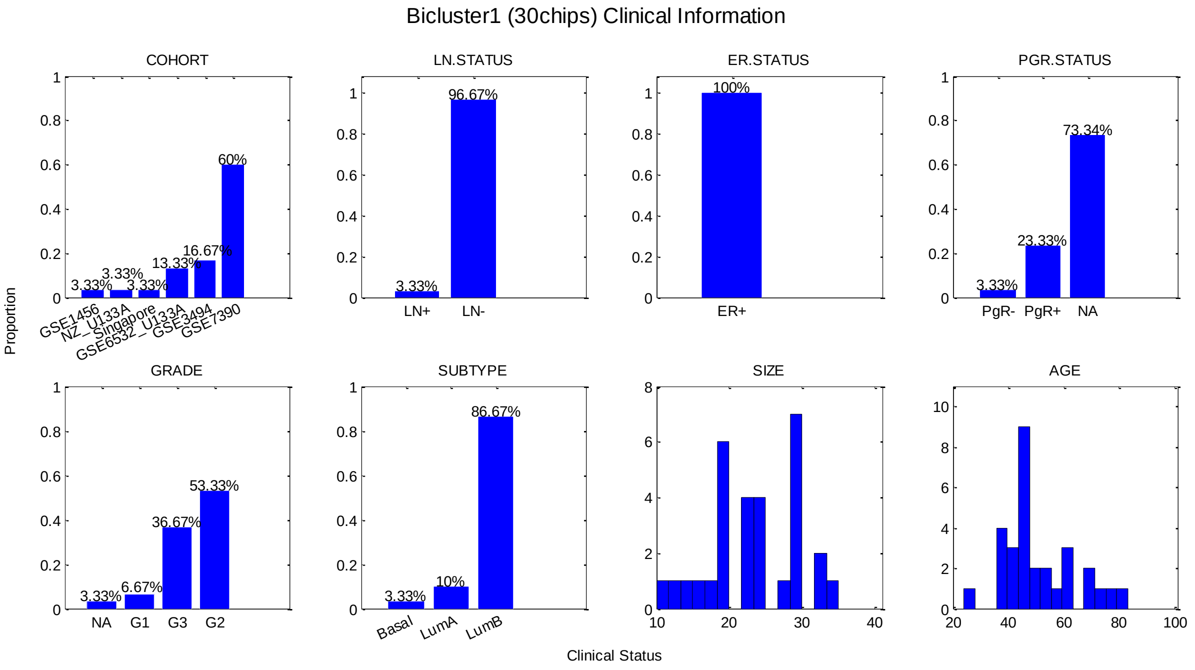

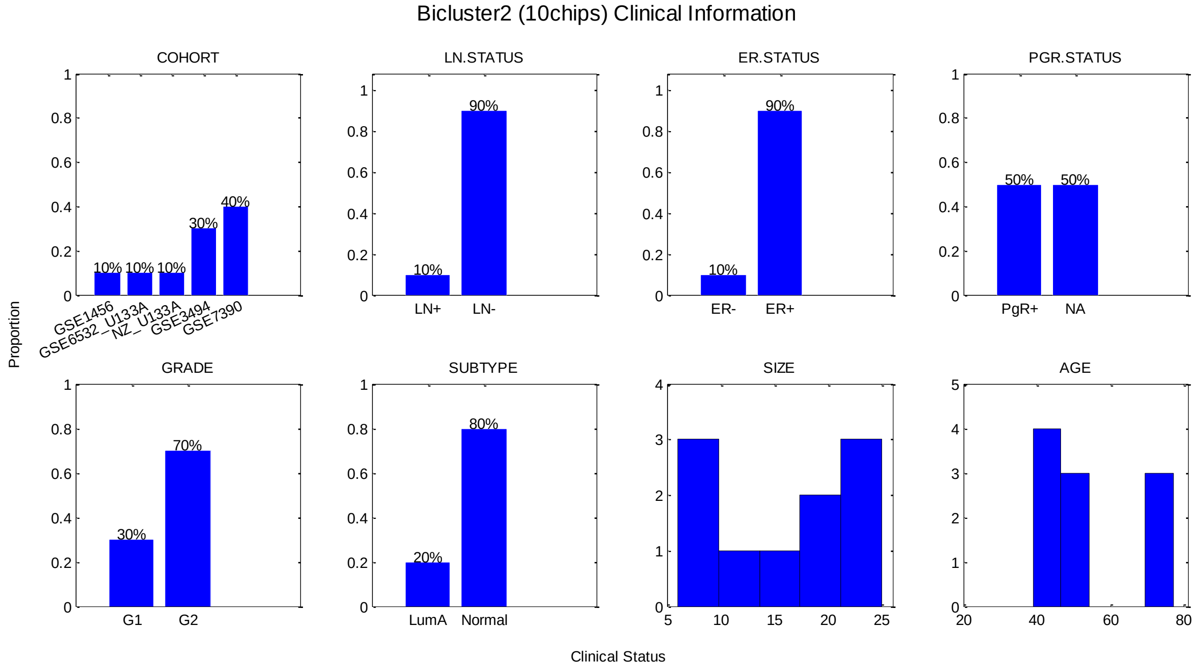

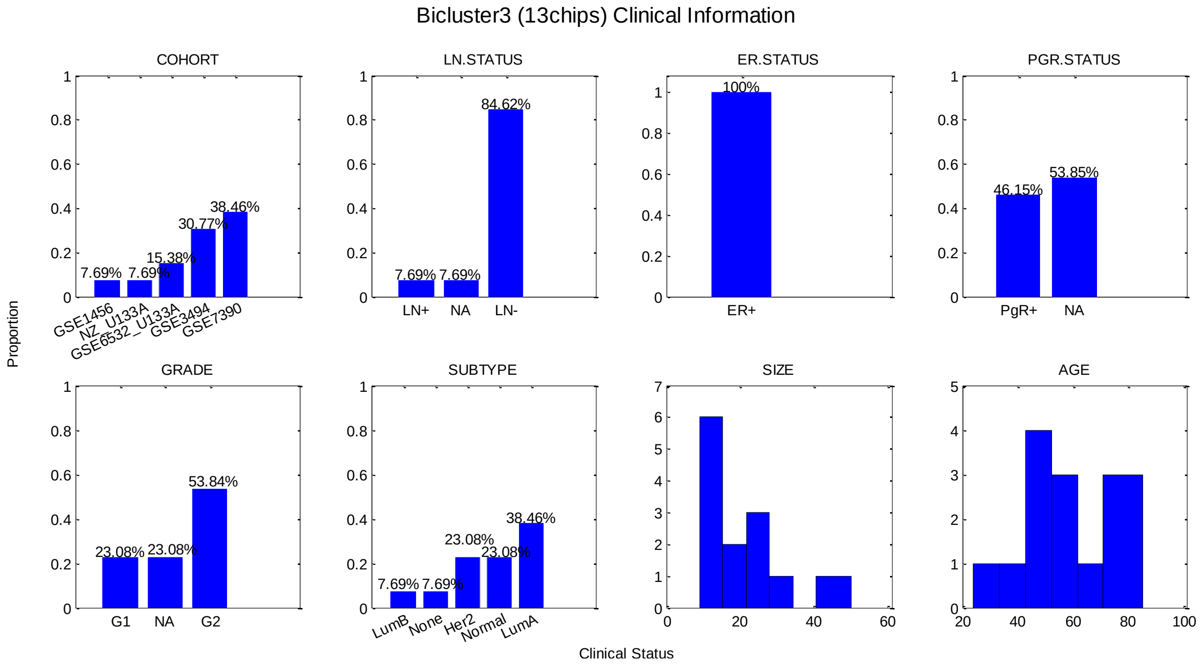
Bicluster3 (13chips) Clinical Information
COHORT
7.69% 7.69% 15.38% 30.77% 38.46%
GSE1456 NZ_U133A GSE6532_U133A GSE3494 GSE7390
LN.STATUS
7.69% 7.69% 84.62%
LN+ NA LN-
ER.STATUS
100%
ER+
PGR.STATUS
46.15% 53.85%
PgR+ NA
GRADE
23.08% 23.08% 53.84%
G1 NA G2
SUBTYPE
7.69% 7.69% 23.08% 23.08% 38.46%
LumB None Her2 Normal LumA
SIZE
AGE
Proportion
Clinical Status

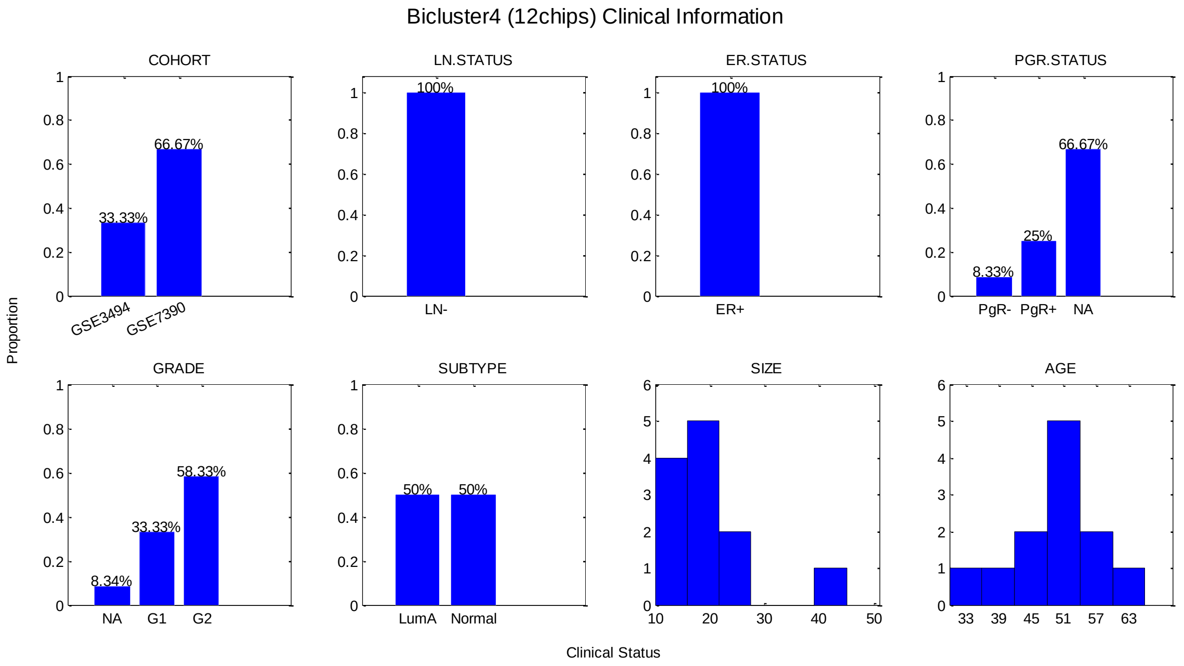
Bicluster4 (12chips) Clinical Information
COHORT
33.33% 66.67%
GSE3494 GSE7390
LN.STATUS
100%
LN-
ER.STATUS
100%
ER+
PGR.STATUS
8.33% 25% 66.67%
PgR- PgR+ NA
GRADE
8.34% 33.33% 58.33%
NA G1 G2
SUBTYPE
50% 50%
LumA Normal
SIZE
AGE
Proportion
Clinical Status

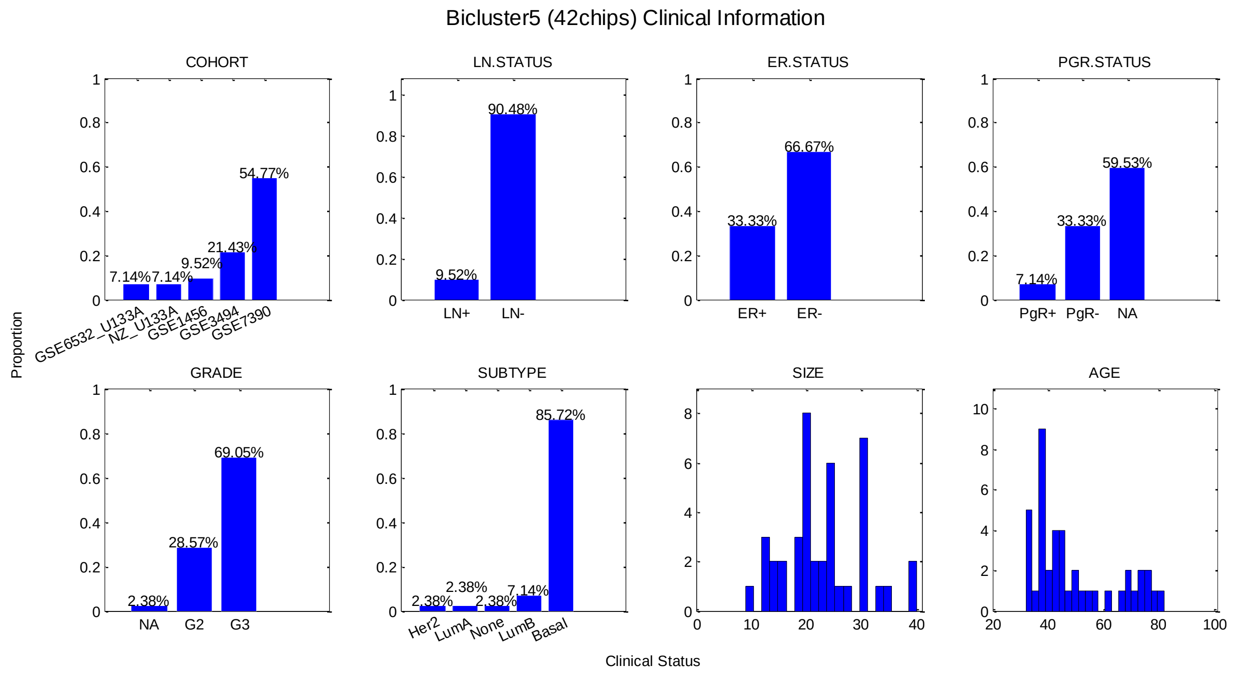
Bicluster5 (42chips) Clinical Information
COHORT
7.14% 7.14% 9.52% 21.43% 54.77%
GSE6532_U133A NZ_U133A GSE1456 GSE3494 GSE7390
LN.STATUS
9.52% 90.48%
LN+ LN-
ER.STATUS
33.33% 66.67%
ER+ ER-
PGR.STATUS
7.14% 33.33% 59.53%
PgR+ PgR- NA
GRADE
2.38% 28.57% 69.05%
NA G2 G3
SUBTYPE
2.38% 2.38% 2.38% 7.14% 85.72%
Her2 LumA None LumB Basal
SIZE
AGE
Proportion
Clinical Status

Bicluster6 (19chips) Clinical Information

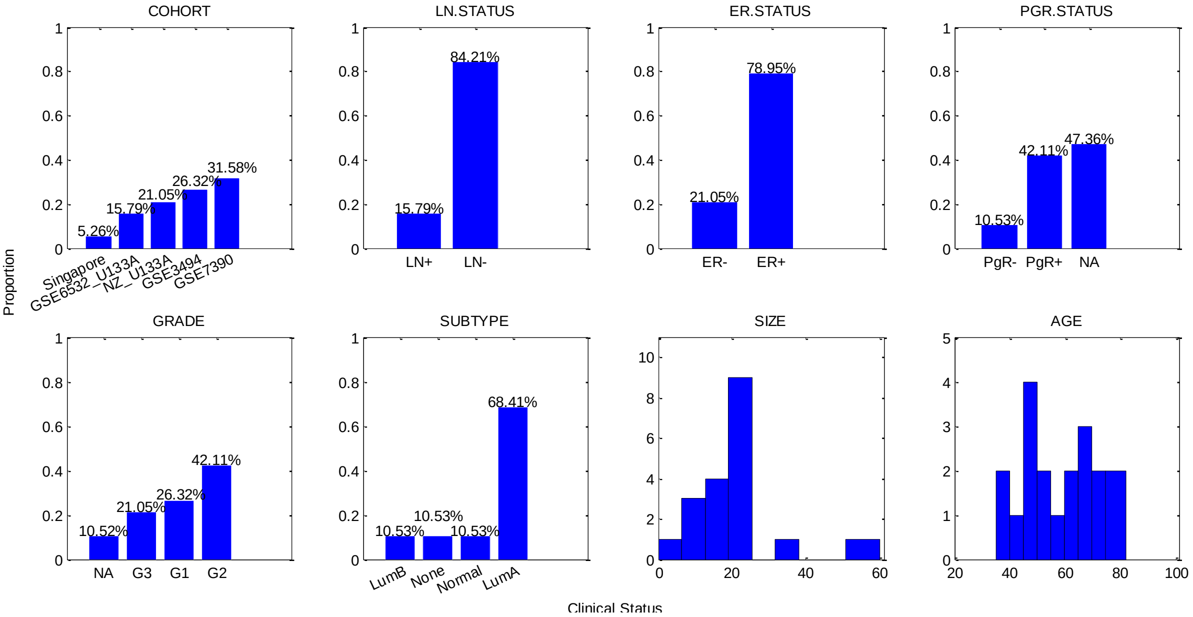

Bicluster7 (31chips) Clinical Information

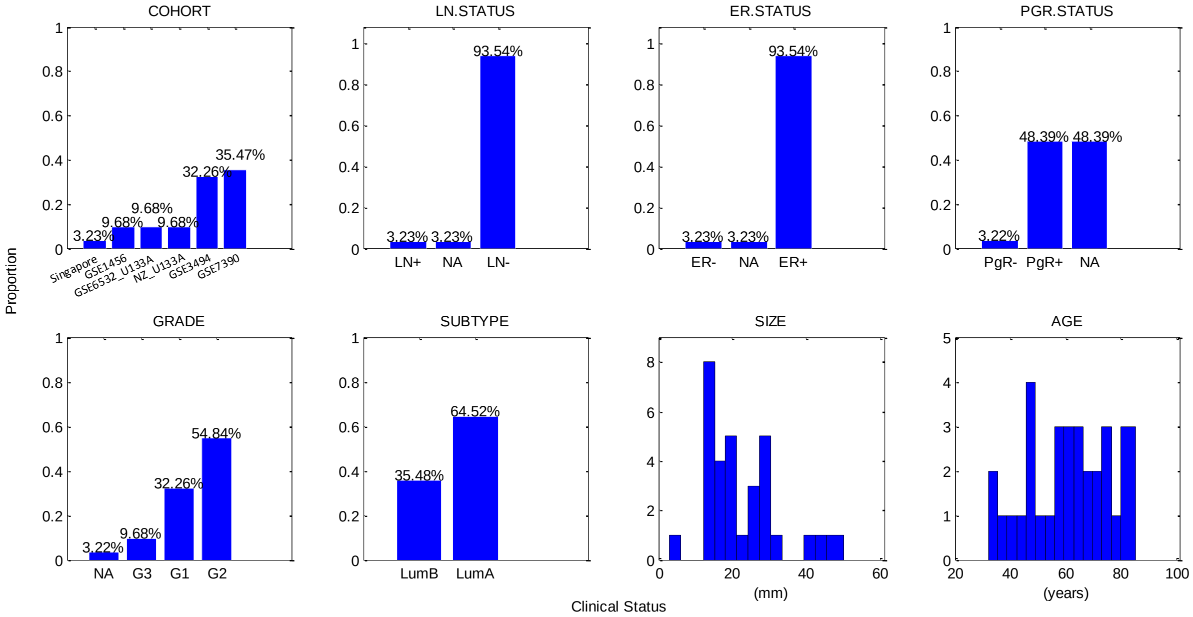

Bicluster8 (10chips) Clinical Information

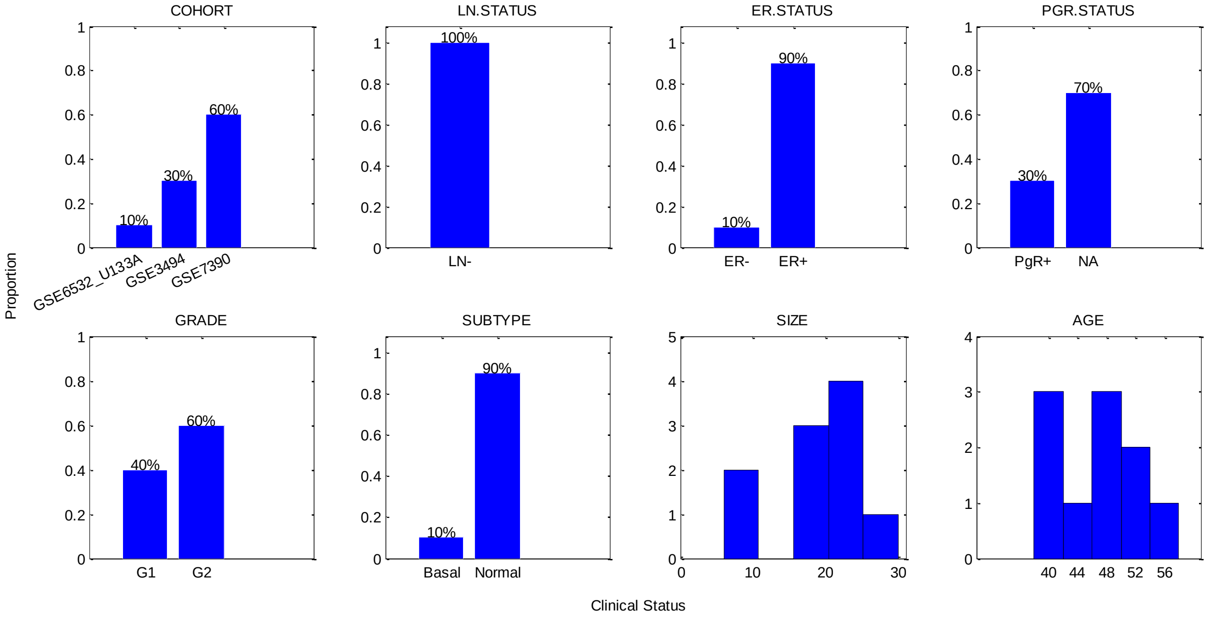

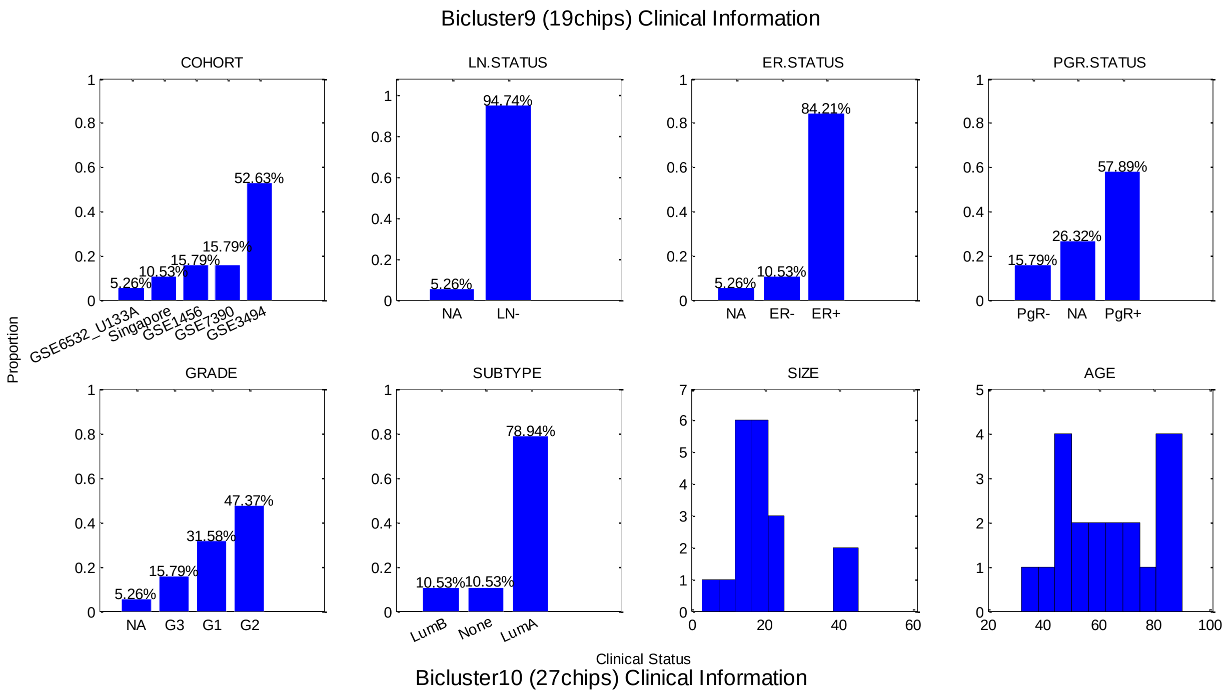
Bicluster9 (19chips) Clinical Information
COHORT
52.63%
15.79%
15.79%
10.53%
5.26%
GSE6532_U133A
Singapore
GSE1456
GSE7390
GSE3494
LN.STATUS
94.74%
5.26%
NA
LN-
ER.STATUS
84.21%
10.53%
5.26%
NA
ER-
ER+
PGR.STATUS
57.89%
26.32%
15.79%
PgR-
NA
PgR+
Proportion
GRADE
47.37%
31.58%
15.79%
5.26%
NA
G3
G1
G2
SUBTYPE
78.94%
10.53%
10.53%
LumB
None
LumA
SIZE
AGE
Clinical Status

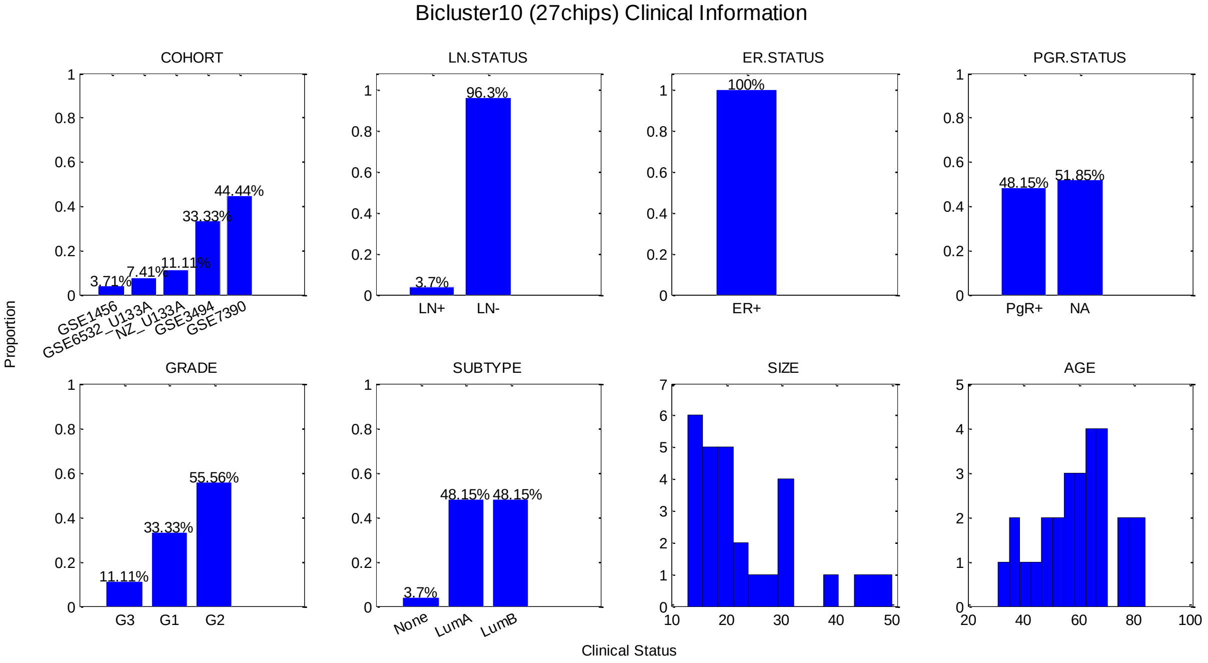
Bicluster10 (27chips) Clinical Information
COHORT
44.44%
33.33%
11.11%
7.41%
3.71%
GSE1456
GSE6532_U133A
NZ_U133A
GSE3494
GSE7390
LN.STATUS
96.3%
3.7%
LN+
LN-
ER.STATUS
100%
ER+
PGR.STATUS
48.15%
51.85%
PgR+
NA
Proportion
GRADE
55.56%
33.33%
11.11%
G3
G1
G2
SUBTYPE
48.15%
48.15%
3.7%
None
LumA
LumB
SIZE
AGE
Clinical Status

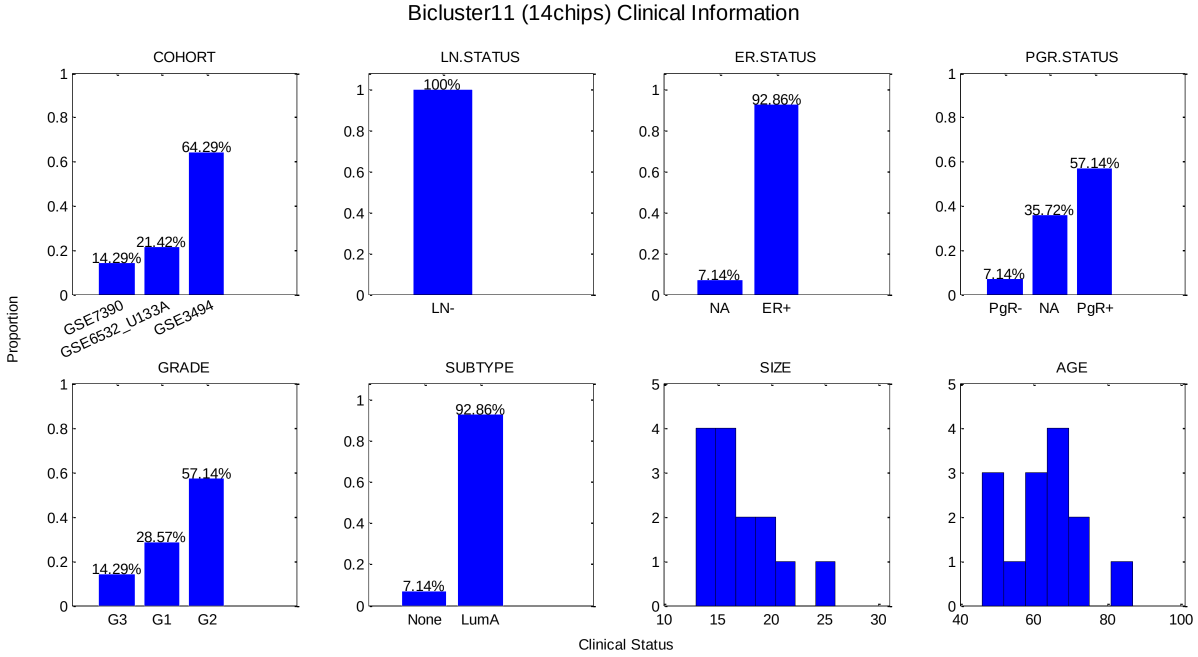
Bicluster11 (14chips) Clinical Information
COHORT
64.29%
21.42%
14.29%
GSE7390
GSE6532_U133A
GSE3494
LN.STATUS
100%
LN-
ER.STATUS
92.86%
7.14%
NA
ER+
PGR.STATUS
57.14%
35.72%
7.14%
PgR-
NA
PgR+
Proportion
GRADE
57.14%
28.57%
14.29%
G3
G1
G2
SUBTYPE
92.86%
7.14%
None
LumA
SIZE
AGE
Clinical Status

Bicluster12 (28chips) Clinical Information

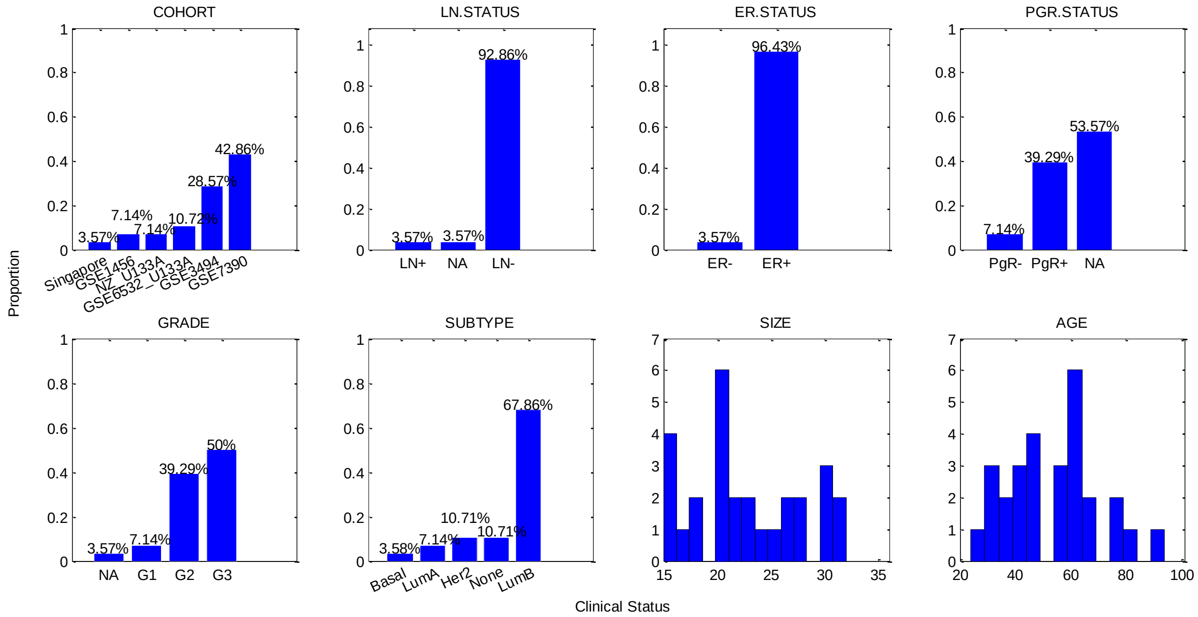

Bicluster13 (13chips) Clinical Information

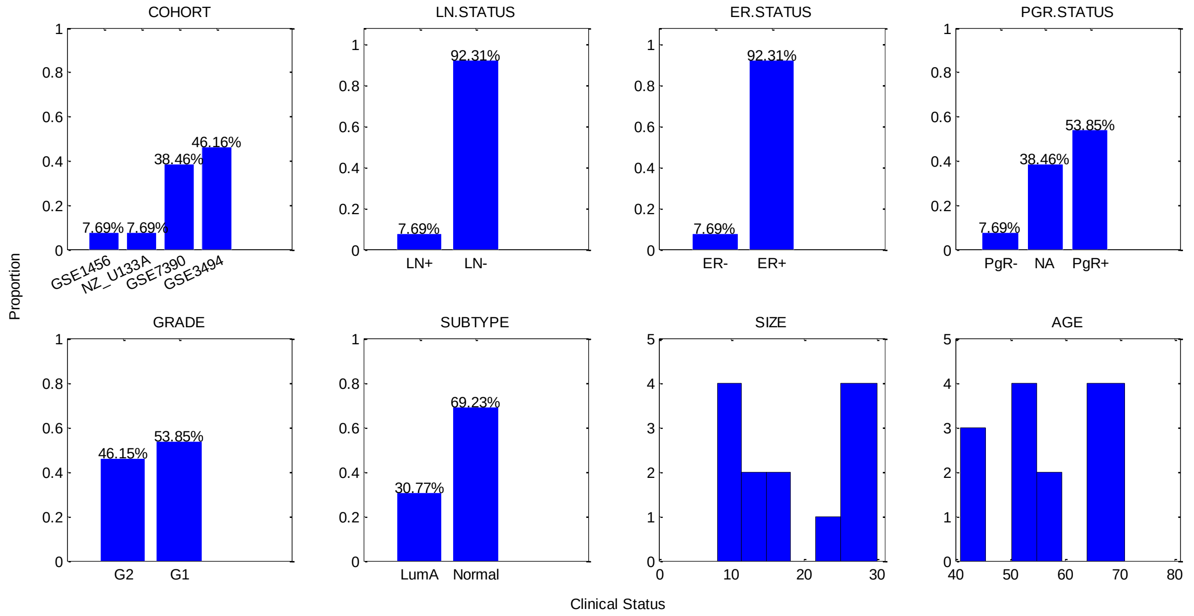

Bicluster14 (13chips) Clinical Information

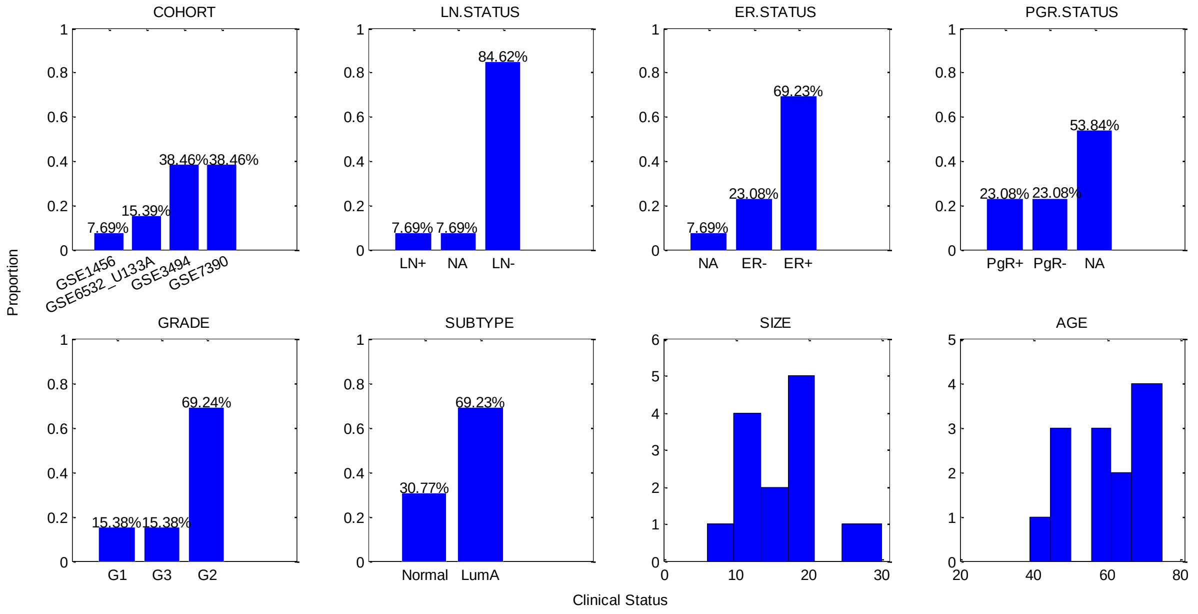

Bicluster15 (16chips) Clinical Information

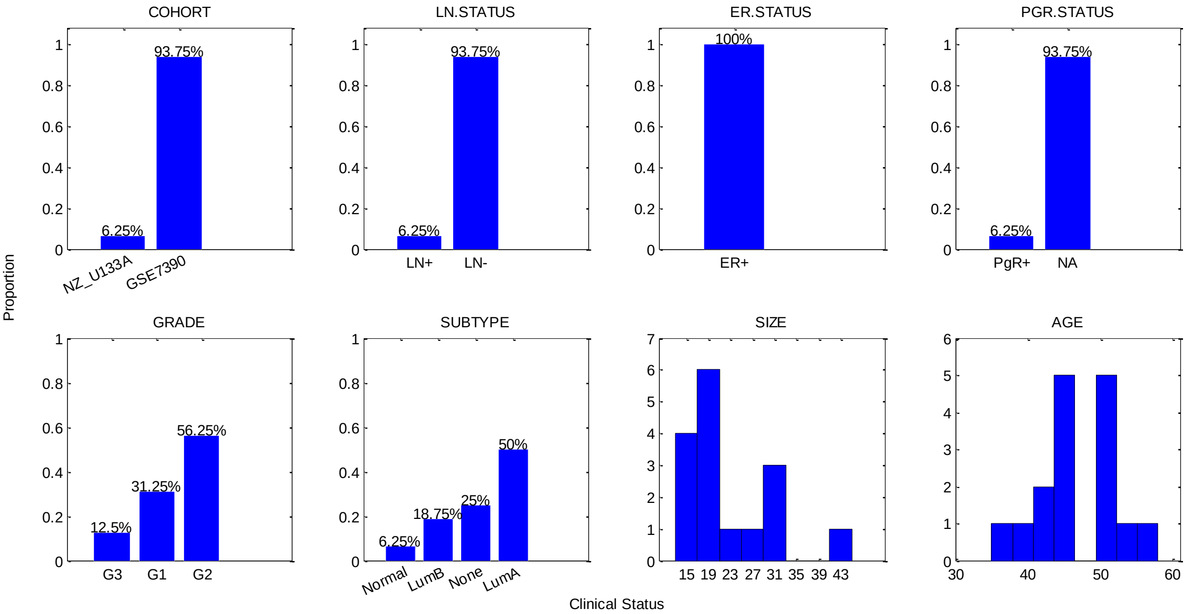

Bicluster16 (14chips) Clinical Information

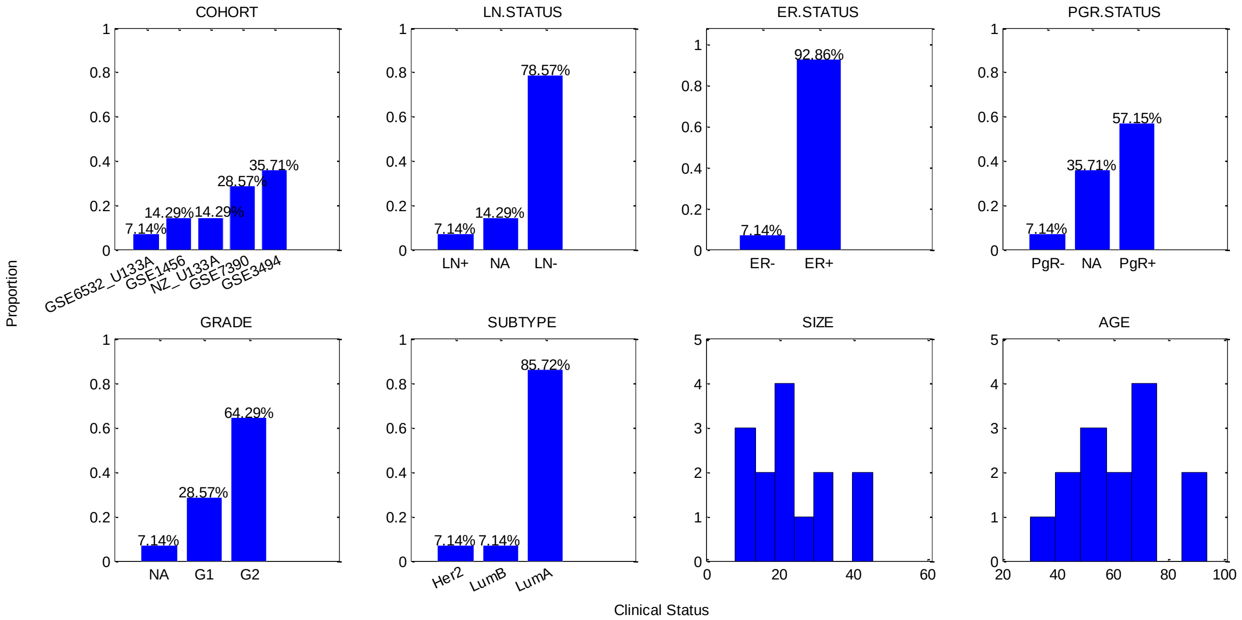

Bicluster17 (23chips) Clinical Information

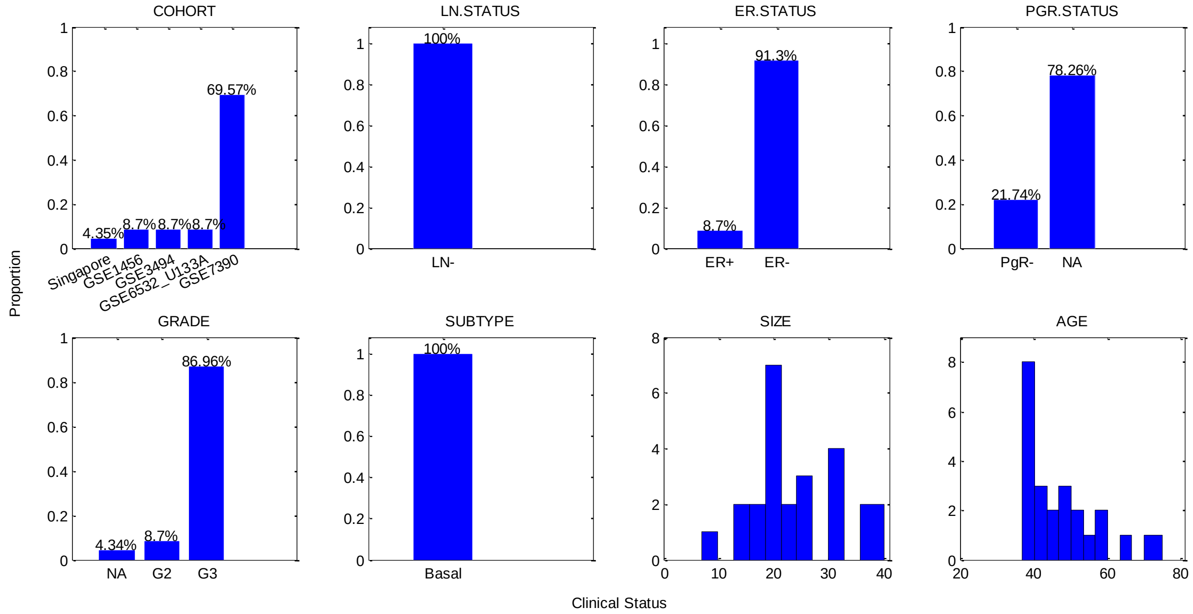

Bicluster18 (26chips) Clinical Information

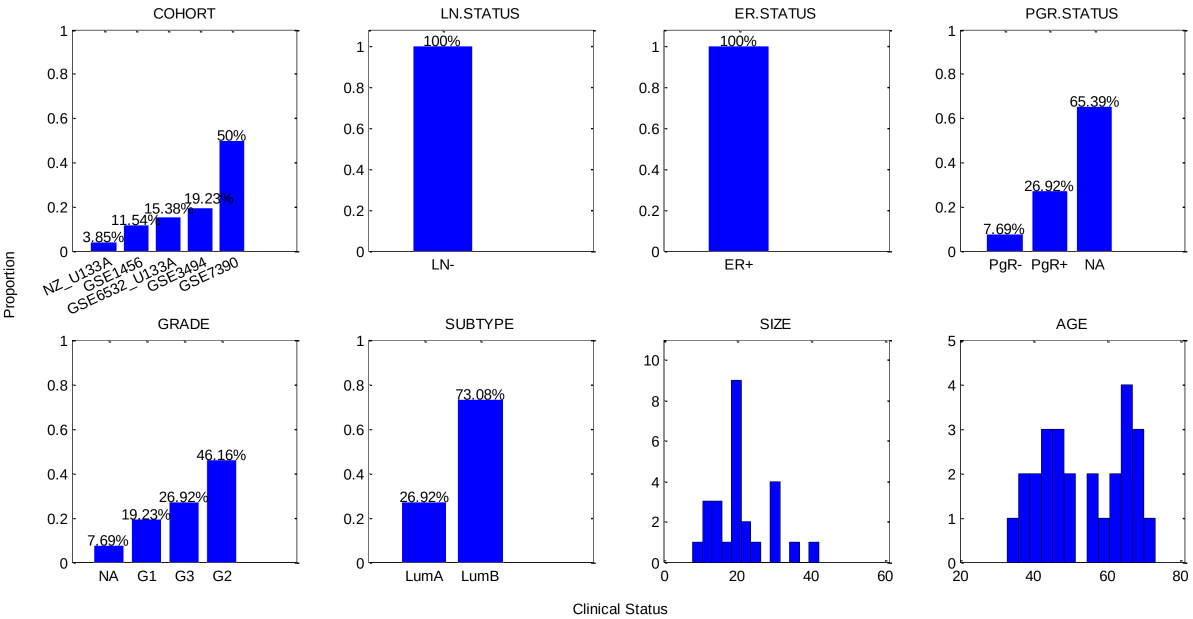

Bicluster19 (12chips) Clinical Information

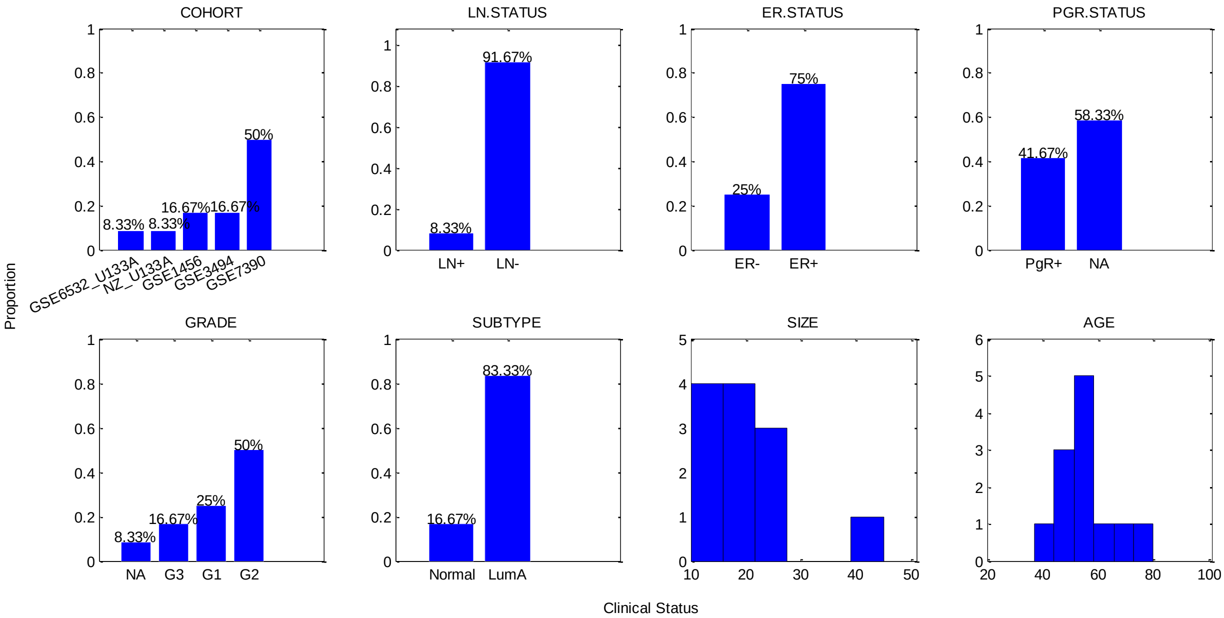

Bicluster20 (27chips) Clinical Information

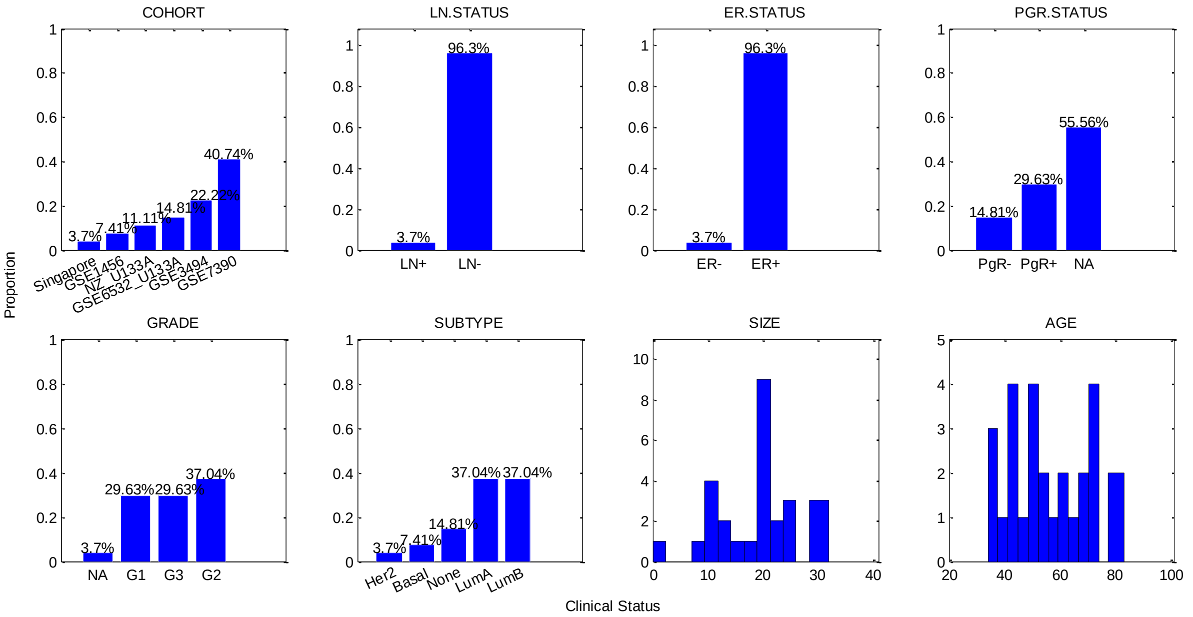

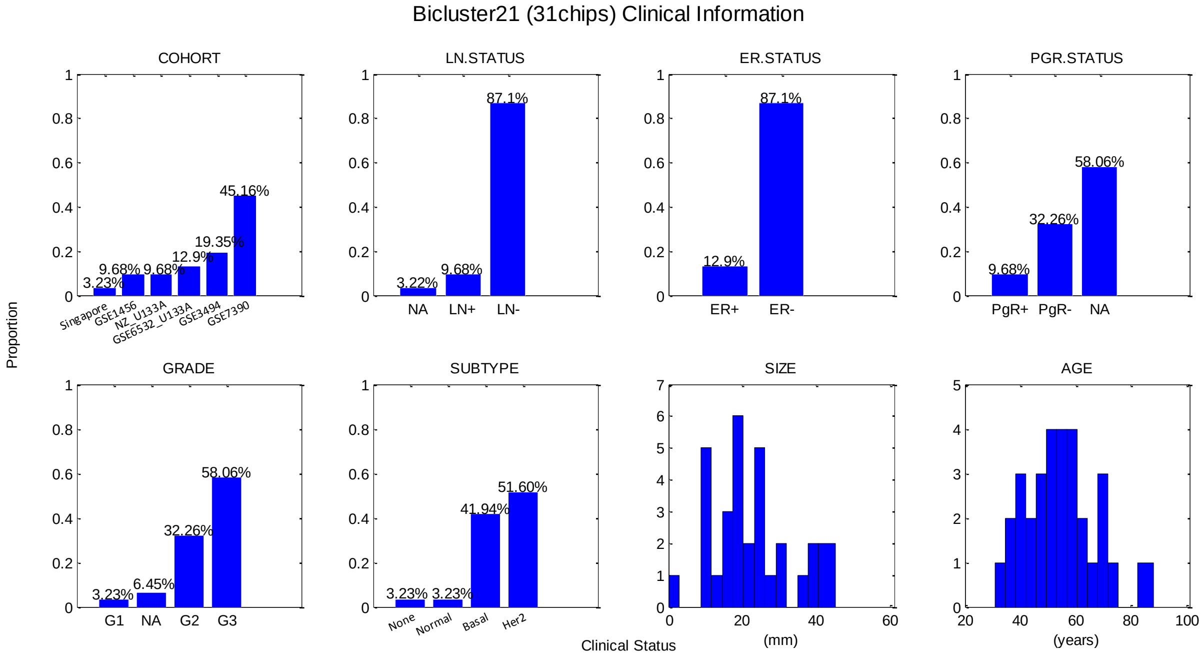
Bicluster21 (31chips) Clinical Information
COHORT
Singapore GSE1456 NZ_U133A GSE6532_U133A GSE3494 GSE7390
3.23% 9.68% 9.68% 12.9% 19.35% 45.16%
LN.STATUS
NA LN+ LN-
3.22% 9.68% 87.1%
ER.STATUS
ER+ ER-
12.9% 87.1%
PGR.STATUS
PgR+ PgR- NA
9.68% 32.26% 58.06%
GRADE
G1 NA G2 G3
3.23% 6.45% 32.26% 58.06%
SUBTYPE
None Normal Basal Her2
3.23% 3.23% 41.94% 51.60%
SIZE
(mm)
AGE
(years)
Proportion
Clinical Status

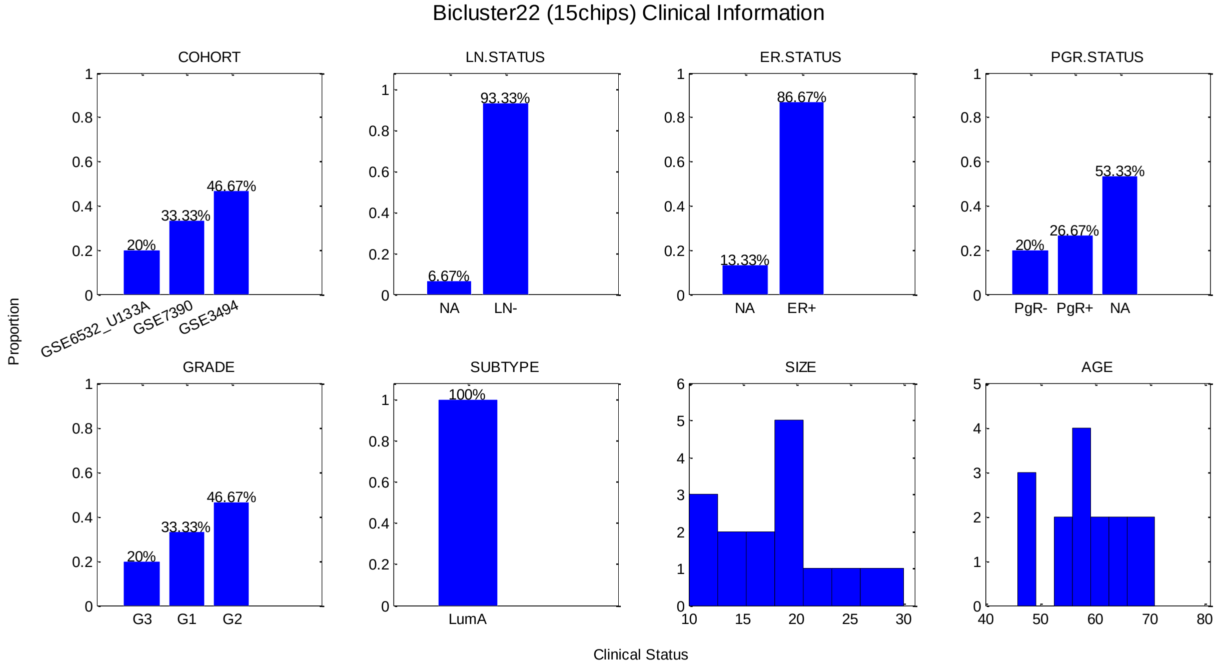
Bicluster22 (15chips) Clinical Information
COHORT
GSE6532_U133A GSE7390 GSE3494
20% 33.33% 46.67%
LN.STATUS
NA LN-
6.67% 93.33%
ER.STATUS
NA ER+
13.33% 86.67%
PGR.STATUS
PgR- PgR+ NA
20% 26.67% 53.33%
GRADE
G3 G1 G2
20% 33.33% 46.67%
SUBTYPE
LumA
100%
SIZE
AGE
Proportion
Clinical Status

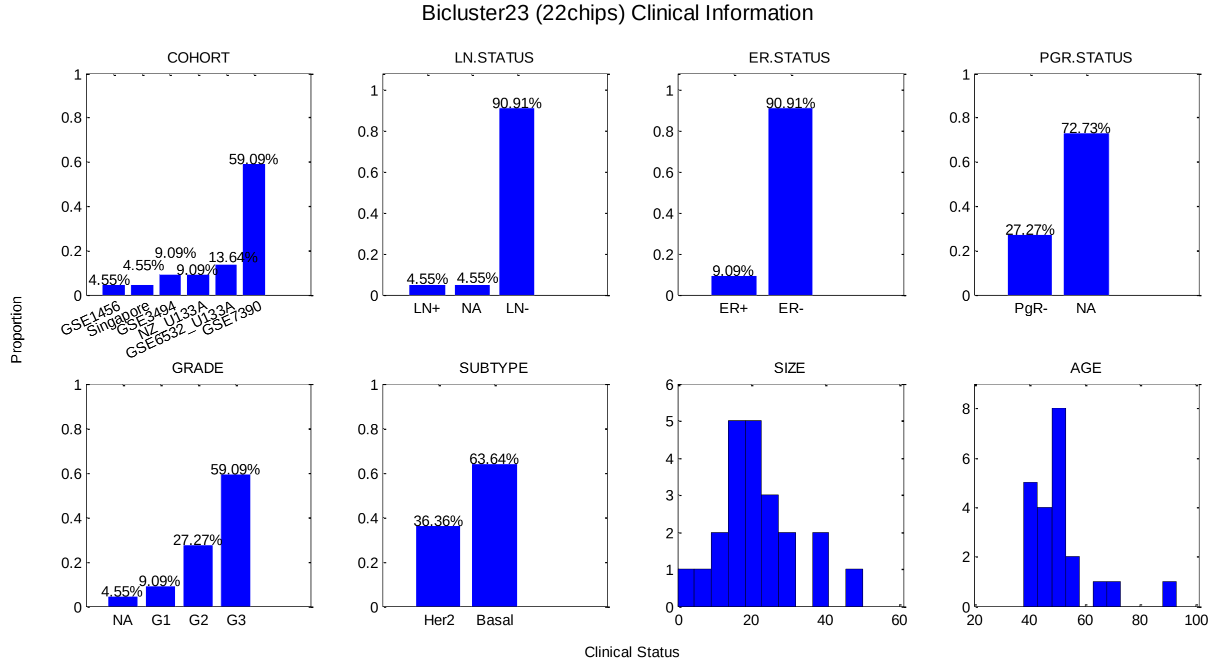
Bicluster23 (22chips) Clinical Information
COHORT
GSE1456 Singapore GSE3494 NZ_U133A GSE6532_U133A GSE7390
4.55% 4.55% 9.09% 9.09% 13.64% 59.09%
LN.STATUS
LN+ NA LN-
4.55% 4.55% 90.91%
ER.STATUS
ER+ ER-
9.09% 90.91%
PGR.STATUS
PgR- NA
27.27% 72.73%
GRADE
NA G1 G2 G3
4.55% 9.09% 27.27% 59.09%
SUBTYPE
Her2 Basal
36.36% 63.64%
SIZE
AGE
Proportion
Clinical Status

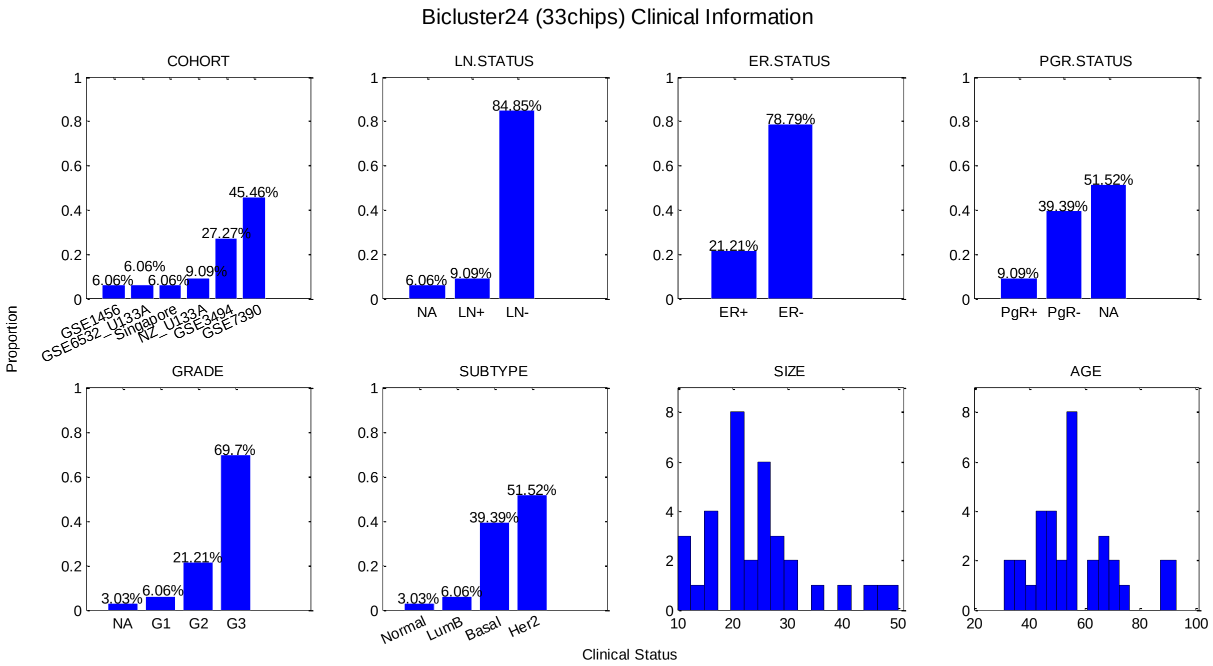
Bicluster24 (33chips) Clinical Information
COHORT
LN.STATUS
ER.STATUS
PGR.STATUS
GRADE
SUBTYPE
SIZE
AGE
Proportion
Clinical Status
6.06%
6.06%
6.06%
9.09%
27.27%
45.46%
GSE1456
GSE6532_U133A
Singapore
NZ_U133A
GSE3494
GSE7390
6.06%
9.09%
84.85%
NA
LN+
LN-
21.21%
78.79%
ER+
ER-
9.09%
39.39%
51.52%
PgR+
PgR-
NA
3.03%
6.06%
21.21%
69.7%
NA
G1
G2
G3
3.03%
6.06%
39.39%
51.52%
Normal
LumB
Basal
Her2

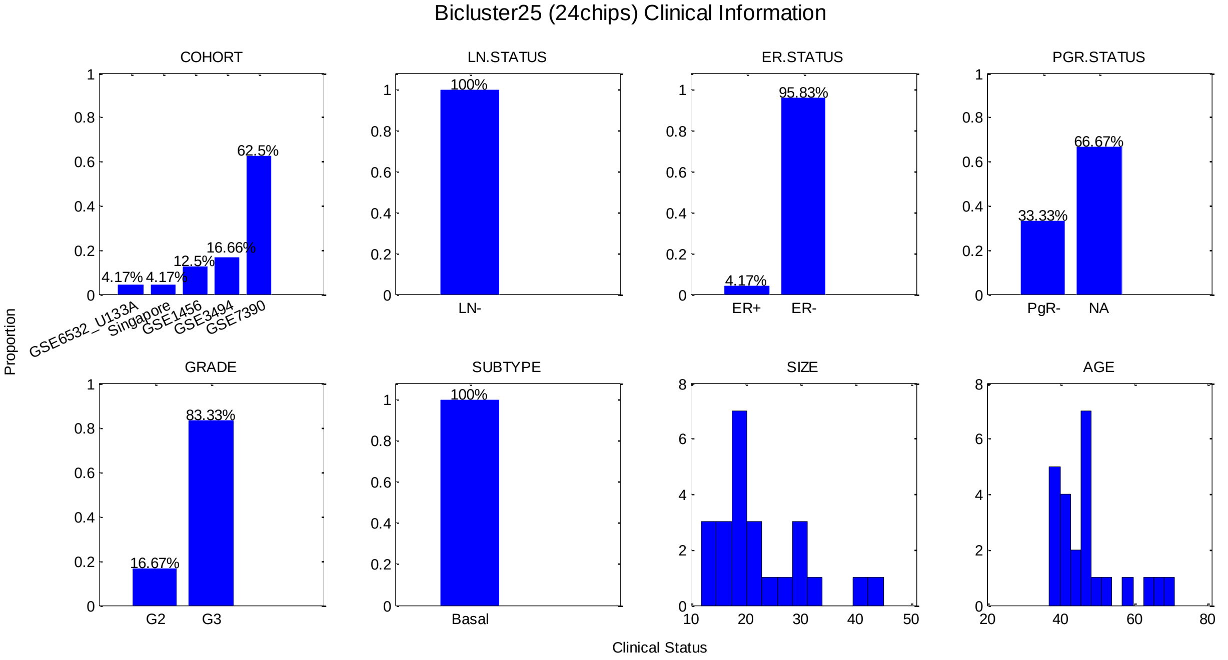
Bicluster25 (24chips) Clinical Information
COHORT
LN.STATUS
ER.STATUS
PGR.STATUS
GRADE
SUBTYPE
SIZE
AGE
Proportion
Clinical Status
4.17%
4.17%
12.5%
16.66%
62.5%
GSE6532_U133A
Singapore
GSE1456
GSE3494
GSE7390
100%
LN-
4.17%
95.83%
ER+
ER-
33.33%
66.67%
PgR-
NA
16.67%
83.33%
G2
G3
100%
Basal

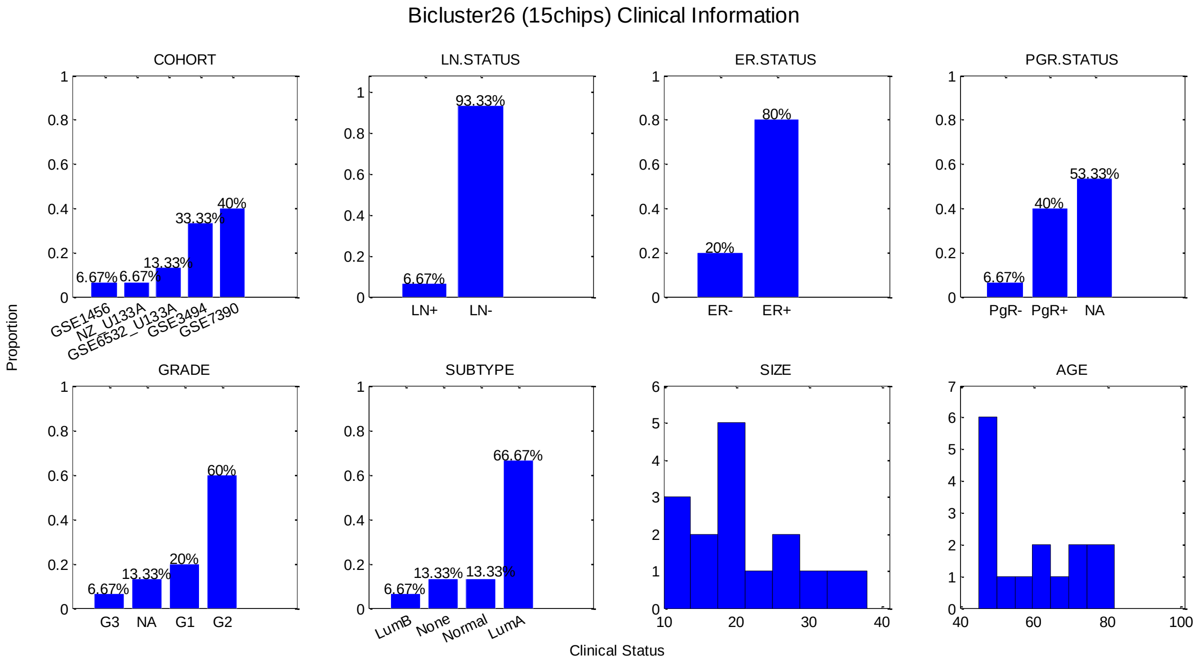
Bicluster26 (15chips) Clinical Information
COHORT
LN.STATUS
ER.STATUS
PGR.STATUS
GRADE
SUBTYPE
SIZE
AGE
Proportion
Clinical Status
6.67%
6.67%
13.33%
33.33%
40%
GSE1456
NZ_U133A
GSE6532_U133A
GSE3494
GSE7390
6.67%
93.33%
LN+
LN-
20%
80%
ER-
ER+
6.67%
40%
53.33%
PgR-
PgR+
NA
6.67%
13.33%
20%
60%
G3
NA
G1
G2
6.67%
13.33%
13.33%
66.67%
LumB
None
Normal
LumA

Bicluster27 (15chips) Clinical Information

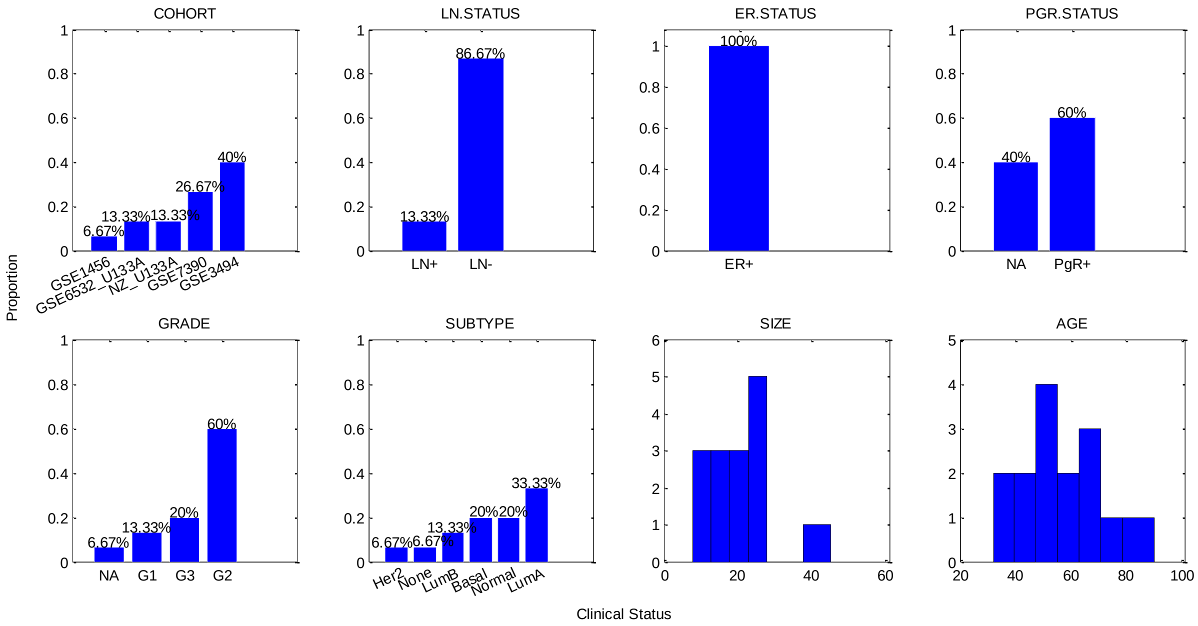

Bicluster28 (16chips) Clinical Information

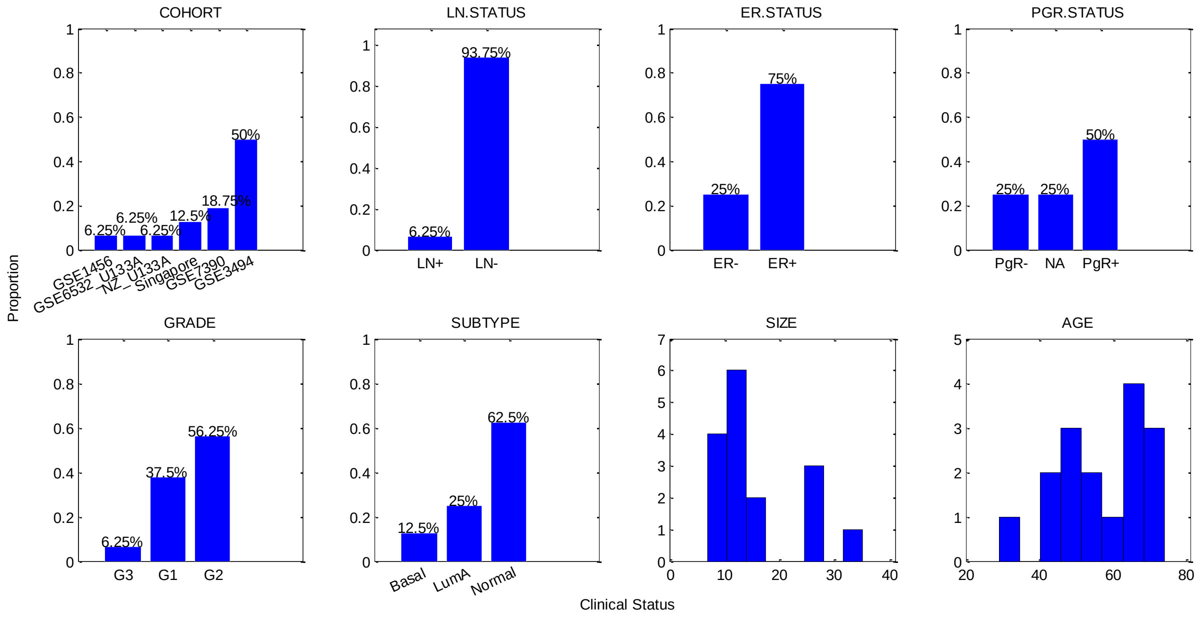

Bicluster29 (23chips) Clinical Information

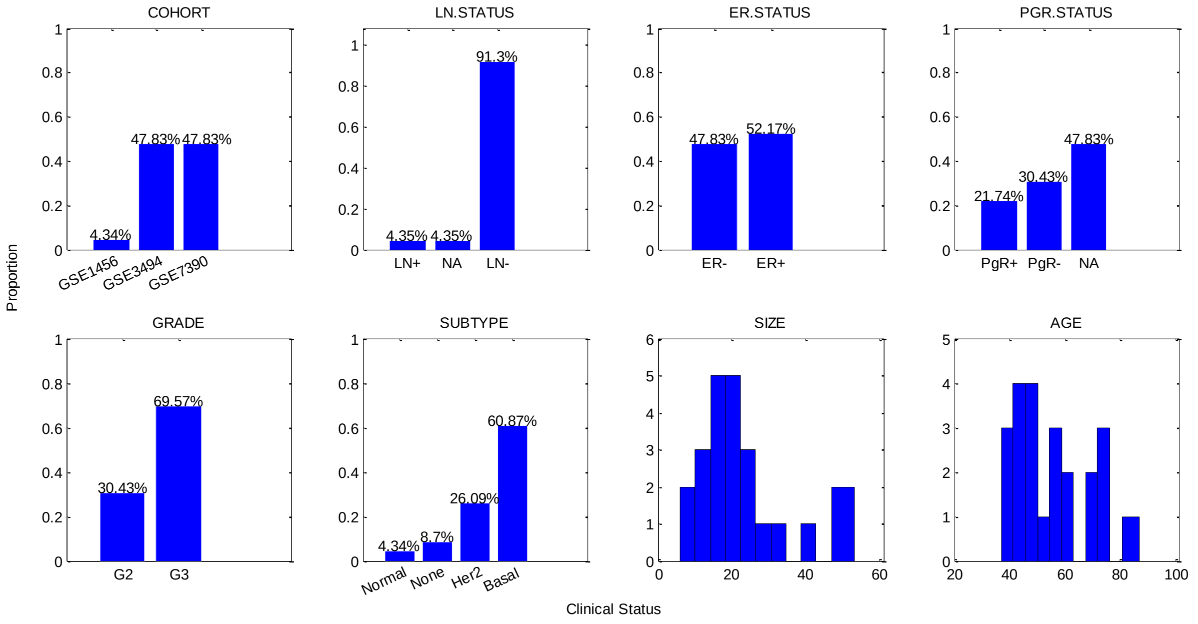

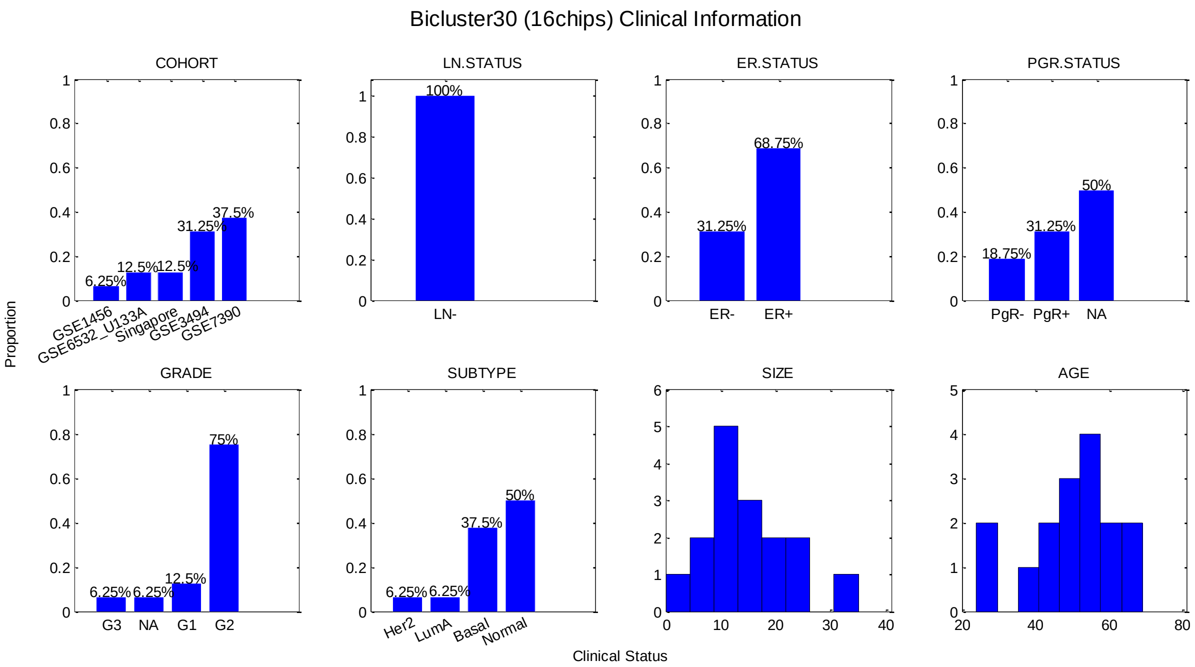
Bicluster30 (16chips) Clinical Information
COHORT
6.25%
12.5%
12.5%
31.25%
37.5%
GSE1456
GSE6532_U133A
Singapore
GSE3494
GSE7390
LN.STATUS
100%
LN-
ER.STATUS
31.25%
68.75%
ER-
ER+
PGR.STATUS
18.75%
31.25%
50%
PgR-
PgR+
NA
Proportion
GRADE
6.25%
6.25%
12.5%
75%
G3
NA
G1
G2
SUBTYPE
6.25%
6.25%
37.5%
50%
Her2
LumA
Basal
Normal
SIZE
AGE
Clinical Status

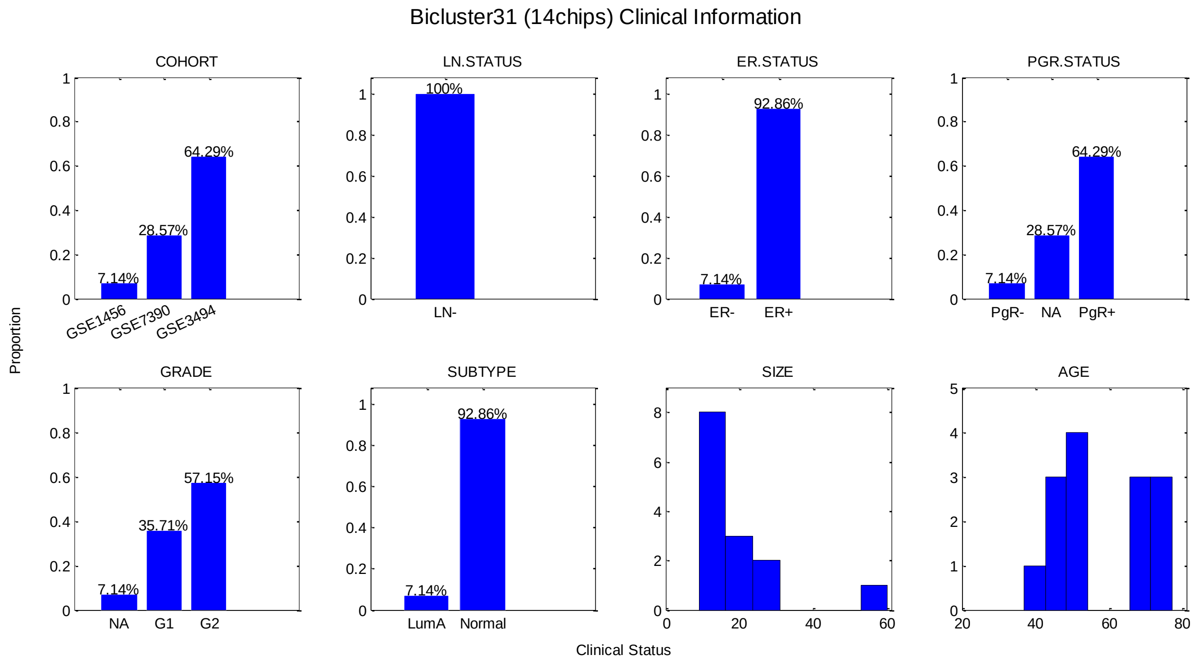
Bicluster31 (14chips) Clinical Information
COHORT
7.14%
28.57%
64.29%
GSE1456
GSE7390
GSE3494
LN.STATUS
100%
LN-
ER.STATUS
7.14%
92.86%
ER-
ER+
PGR.STATUS
7.14%
28.57%
64.29%
PgR-
NA
PgR+
Proportion
GRADE
7.14%
35.71%
57.15%
NA
G1
G2
SUBTYPE
7.14%
92.86%
LumA
Normal
SIZE
AGE
Clinical Status

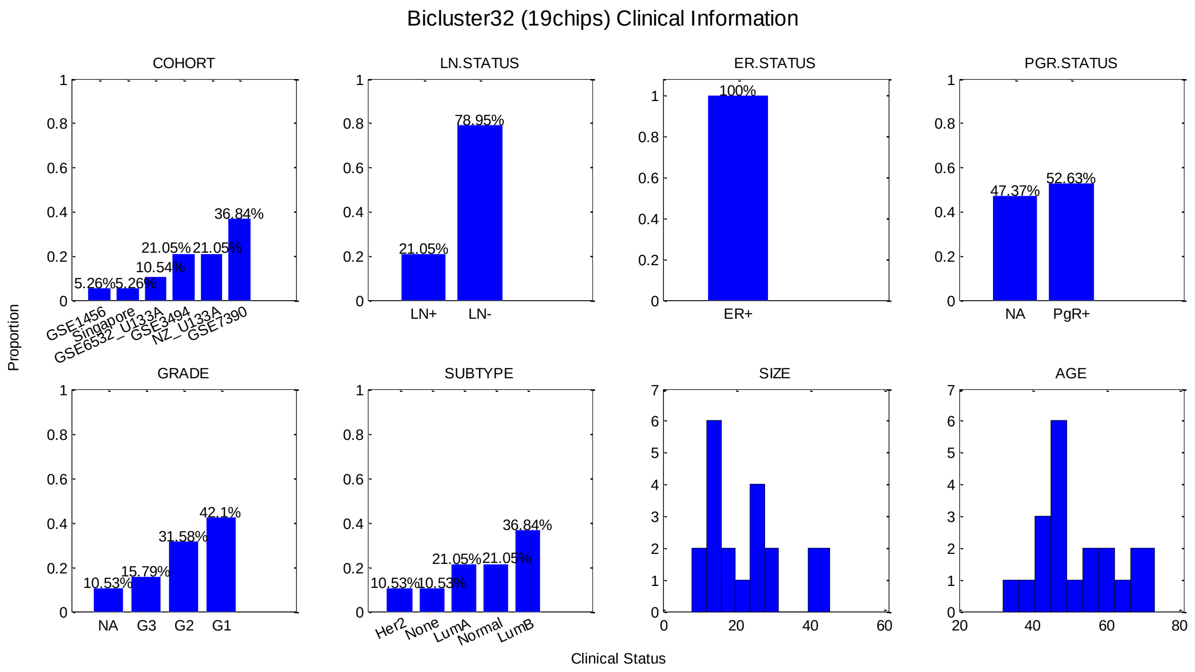
Bicluster32 (19chips) Clinical Information
COHORT
5.26%
5.26%
10.54%
21.05%
21.05%
36.84%
GSE1456
Singapore
GSE6532_U133A
GSE3494
NZ_U133A
GSE7390
LN.STATUS
21.05%
78.95%
LN+
LN-
ER.STATUS
100%
ER+
PGR.STATUS
47.37%
52.63%
NA
PgR+
Proportion
GRADE
10.53%
15.79%
31.58%
42.1%
NA
G3
G2
G1
SUBTYPE
10.53%
10.53%
21.05%
21.05%
36.84%
Her2
None
LumA
Normal
LumB
SIZE
AGE
Clinical Status

Bicluster33 (16chips) Clinical Information

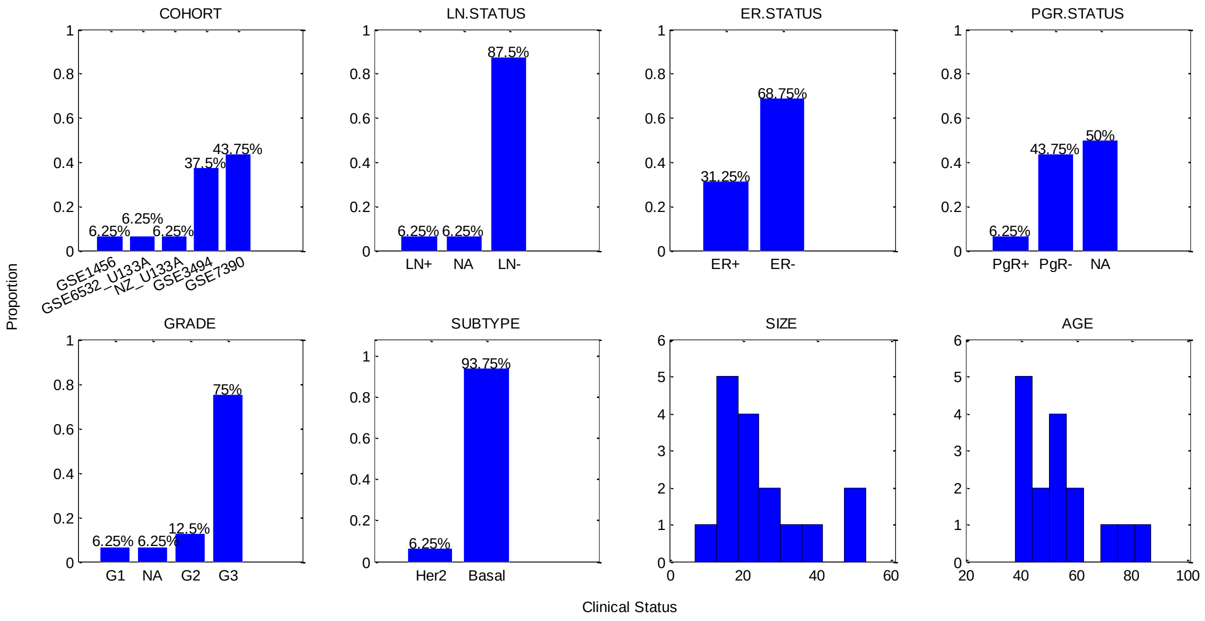

Bicluster34 (18chips) Clinical Information

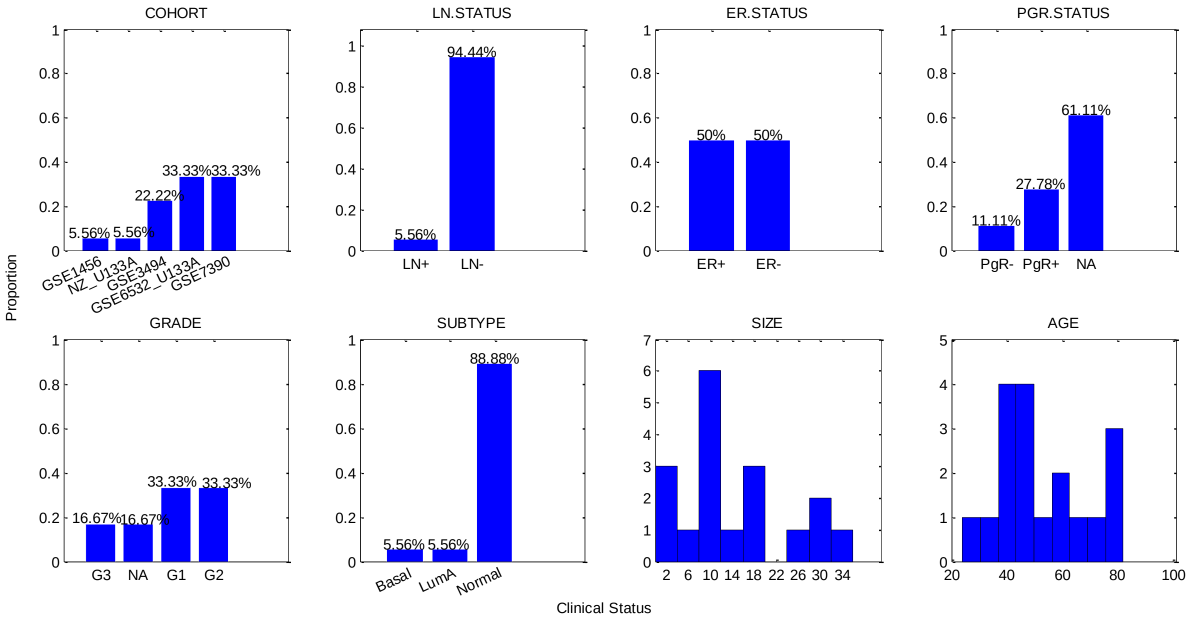

Bicluster35 (21chips) Clinical Information

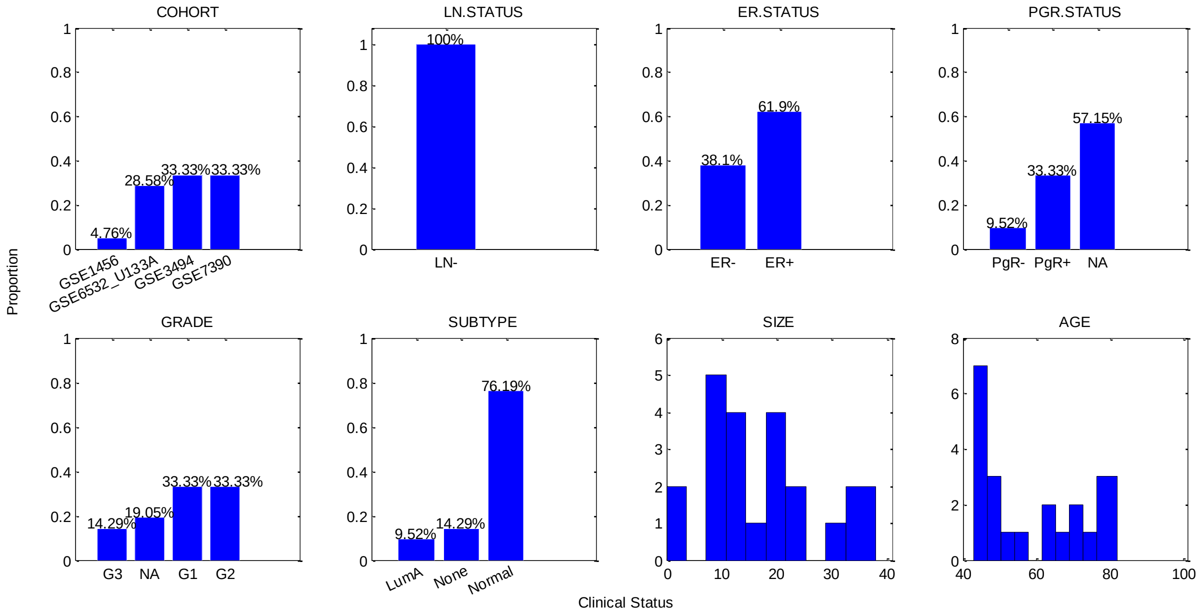

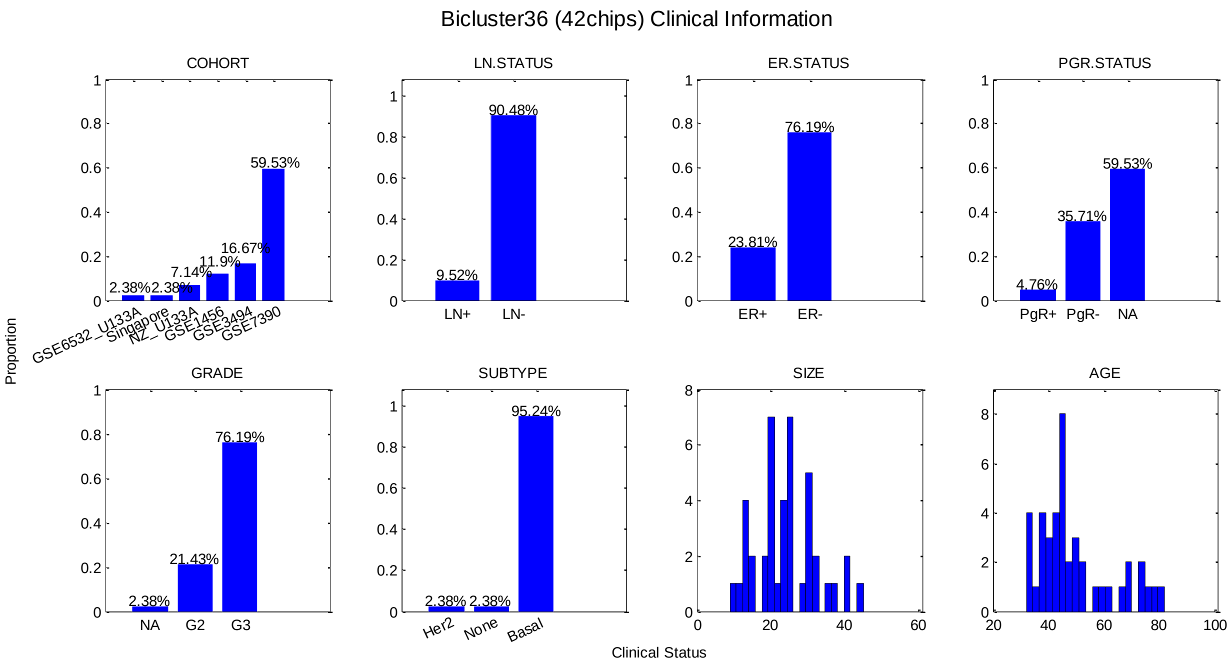
Bicluster36 (42chips) Clinical Information
COHORT
2.38% 2.38% 7.14% 11.9% 16.67% 59.53%
GSE6532_U133A Singapore NZ_U133A GSE1456 GSE3494 GSE7390
LN.STATUS
9.52% 90.48%
LN+ LN-
ER.STATUS
23.81% 76.19%
ER+ ER-
PGR.STATUS
4.76% 35.71% 59.53%
PgR+ PgR- NA
Proportion
GRADE
2.38% 21.43% 76.19%
NA G2 G3
SUBTYPE
2.38% 2.38% 95.24%
Her2 None Basal
SIZE
AGE
Clinical Status

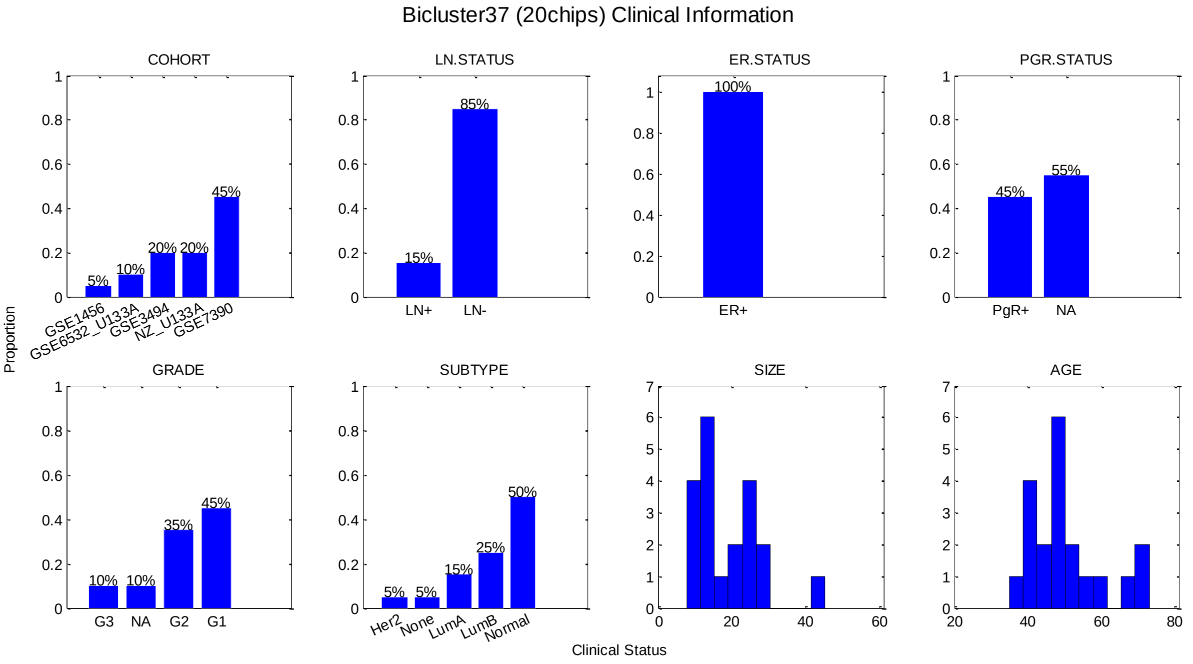
Bicluster37 (20chips) Clinical Information
COHORT
5% 10% 20% 20% 45%
GSE1456 GSE6532_U133A GSE3494 NZ_U133A GSE7390
LN.STATUS
15% 85%
LN+ LN-
ER.STATUS
100%
ER+
PGR.STATUS
45% 55%
PgR+ NA
Proportion
GRADE
10% 10% 35% 45%
G3 NA G2 G1
SUBTYPE
5% 5% 15% 25% 50%
Her2 None LumA LumB Normal
SIZE
AGE
Clinical Status

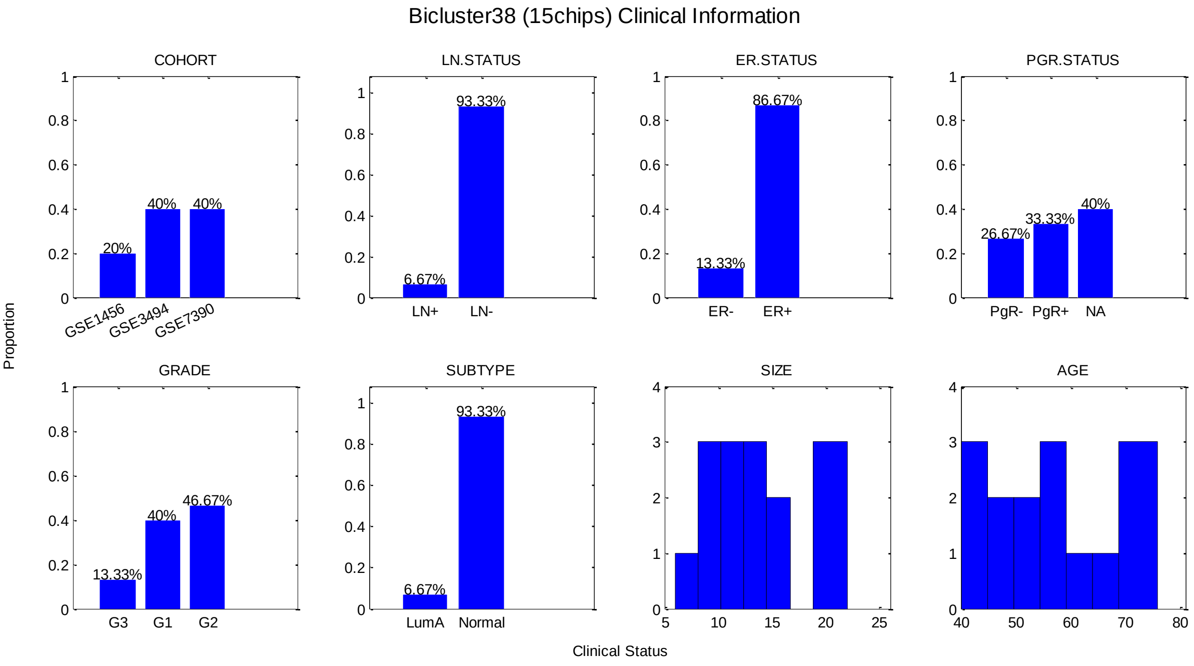
Bicluster38 (15chips) Clinical Information
COHORT
20% 40% 40%
GSE1456 GSE3494 GSE7390
LN.STATUS
6.67% 93.33%
LN+ LN-
ER.STATUS
13.33% 86.67%
ER- ER+
PGR.STATUS
26.67% 33.33% 40%
PgR- PgR+ NA
Proportion
GRADE
13.33% 40% 46.67%
G3 G1 G2
SUBTYPE
6.67% 93.33%
LumA Normal
SIZE
AGE
Clinical Status

Bicluster39 (15chips) Clinical Information

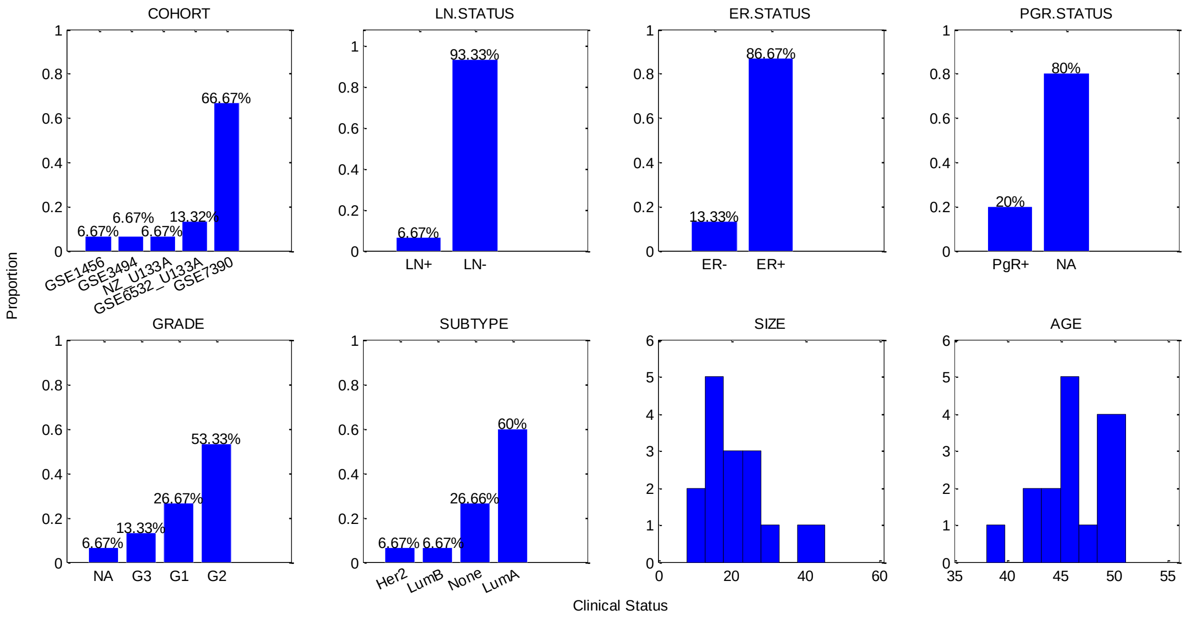

Bicluster40 (26chips) Clinical Information

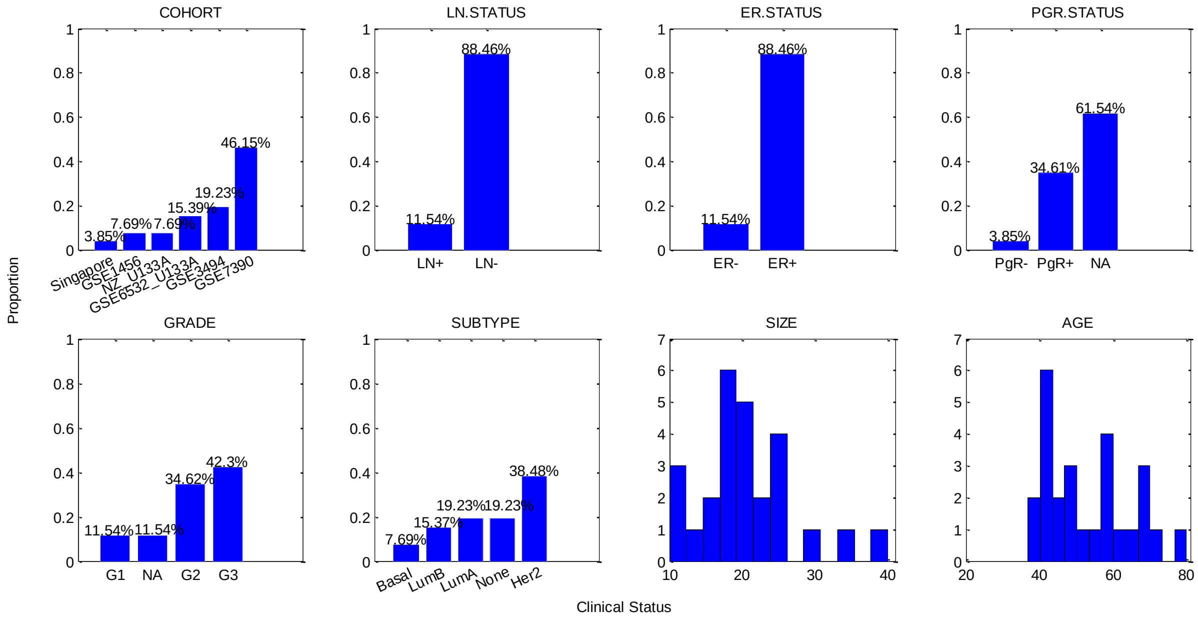

Bicluster41 (18chips) Clinical Information

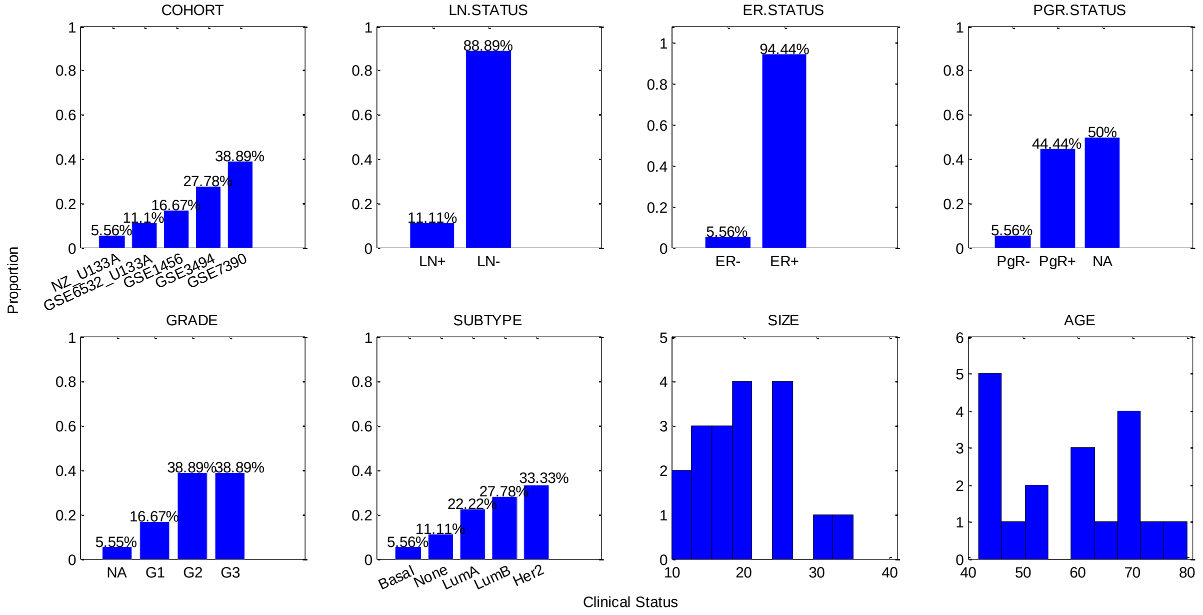

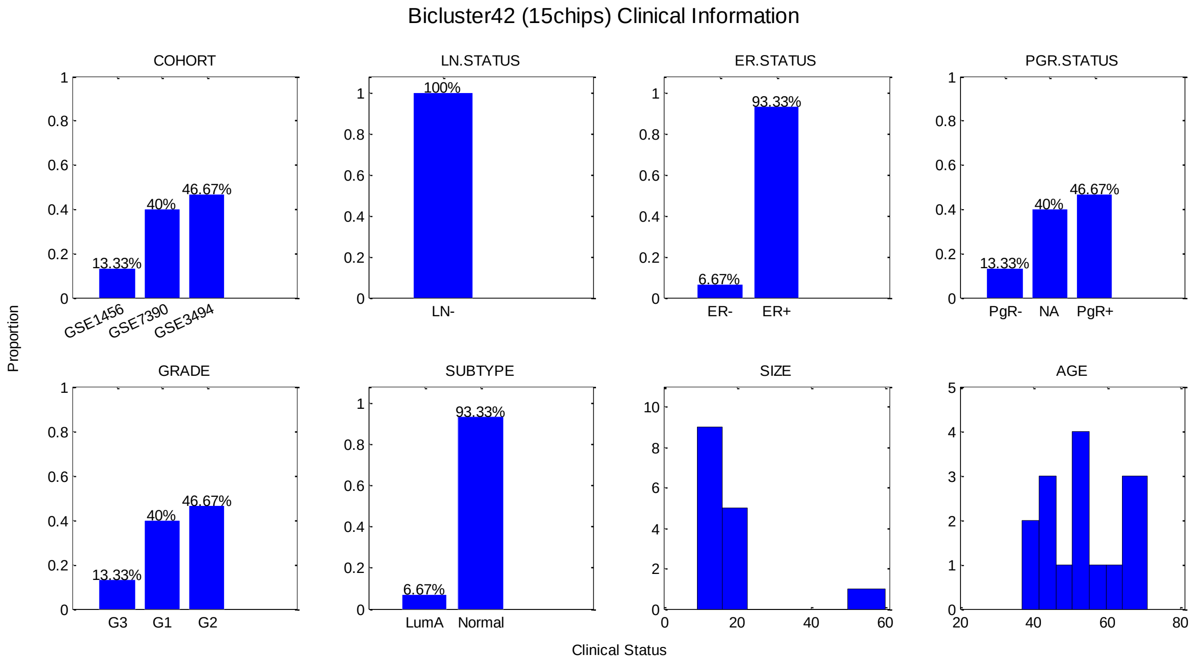
Bicluster42 (15chips) Clinical Information
COHORT
13.33%
40%
46.67%
GSE1456
GSE7390
GSE3494
LN.STATUS
100%
LN-
ER.STATUS
6.67%
93.33%
ER-
ER+
PGR.STATUS
13.33%
40%
46.67%
PgR-
NA
PgR+
Proportion
GRADE
13.33%
40%
46.67%
G3
G1
G2
SUBTYPE
6.67%
93.33%
LumA
Normal
SIZE
AGE
Clinical Status

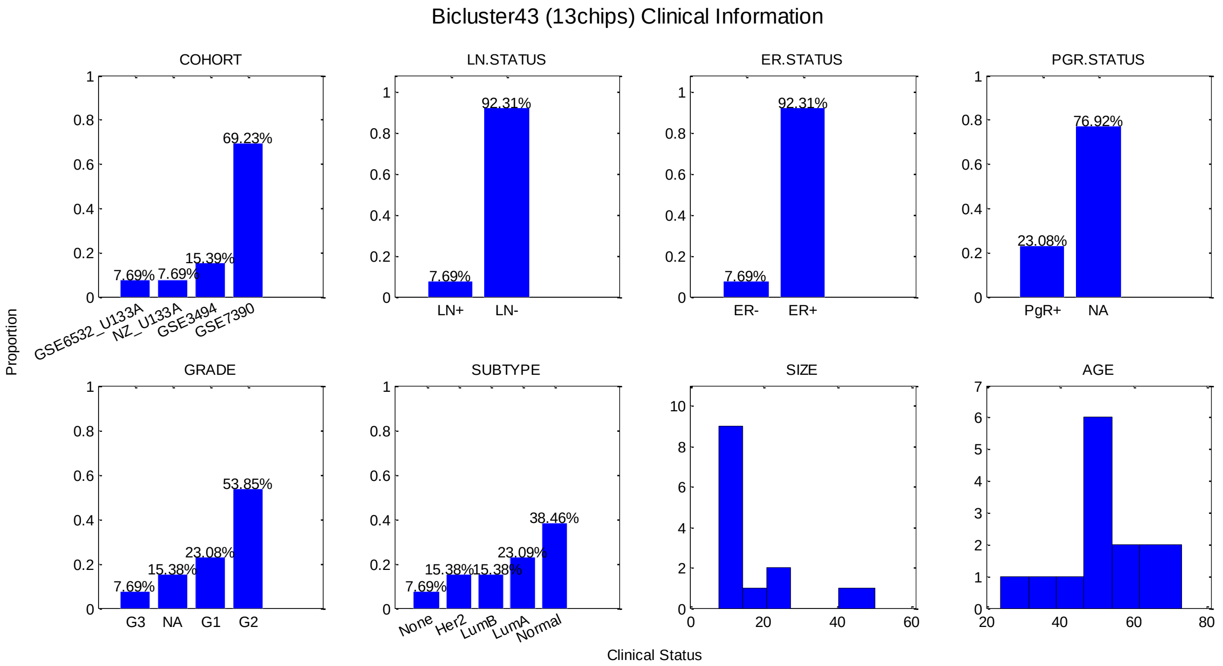
Bicluster43 (13chips) Clinical Information
COHORT
7.69%
7.69%
15.39%
69.23%
GSE6532_U133A
NZ_U133A
GSE3494
GSE7390
LN.STATUS
7.69%
92.31%
LN+
LN-
ER.STATUS
7.69%
92.31%
ER-
ER+
PGR.STATUS
23.08%
76.92%
PgR+
NA
Proportion
GRADE
7.69%
15.38%
23.08%
53.85%
G3
NA
G1
G2
SUBTYPE
7.69%
15.38%
15.38%
23.09%
38.46%
None
Her2
LumB
LumA
Normal
SIZE
AGE
Clinical Status

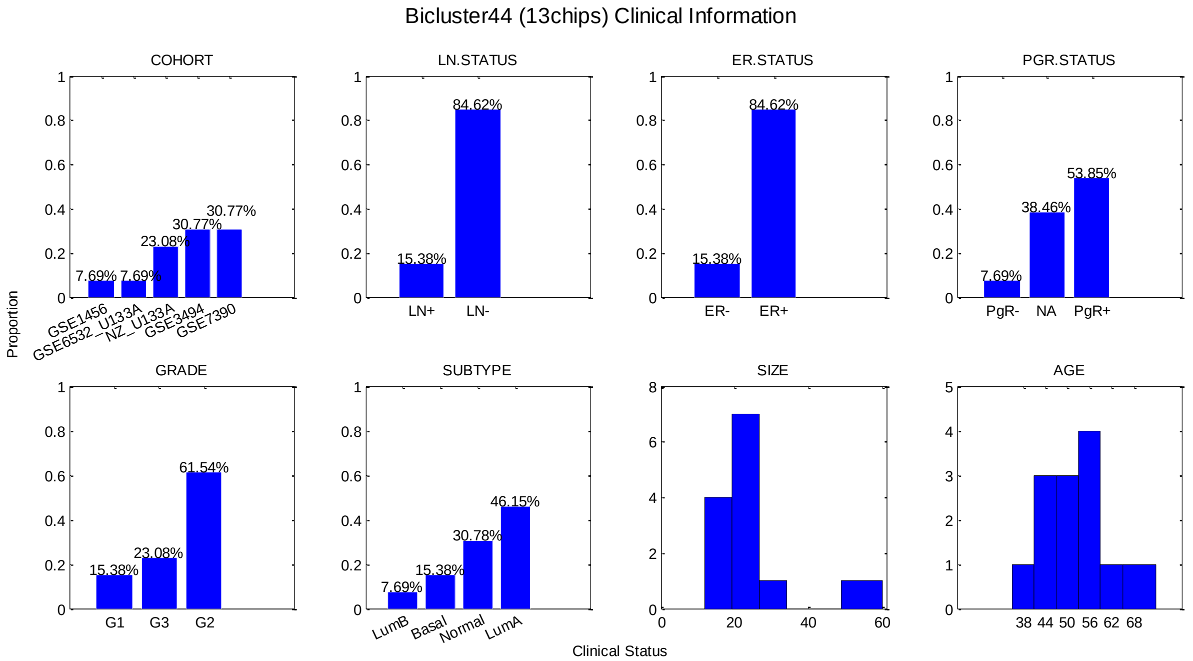
Bicluster44 (13chips) Clinical Information
COHORT
7.69%
7.69%
23.08%
30.77%
30.77%
GSE1456
GSE6532_U133A
NZ_U133A
GSE3494
GSE7390
LN.STATUS
15.38%
84.62%
LN+
LN-
ER.STATUS
15.38%
84.62%
ER-
ER+
PGR.STATUS
7.69%
38.46%
53.85%
PgR-
NA
PgR+
Proportion
GRADE
15.38%
23.08%
61.54%
G1
G3
G2
SUBTYPE
7.69%
15.38%
30.78%
46.15%
LumB
Basal
Normal
LumA
SIZE
AGE
38 44 50 56 62 68
Clinical Status

Supplementary Figure 2. Kaplan-Meier estimates of survival distribution of randomly selected patient groups. Kaplan-Meier estimate of disease free survival (DFS) for 44 groups with arbitrary patient membership (all patients were randomly allocated into 44 groups), superimposed on survival curve of (A) histological grades and (B) tumour subtypes, respectively. Distribution of bicluster-associated survival curves in Figure 4 is much broader than the distribution of patient group-associated survival curves by chance.

(A)

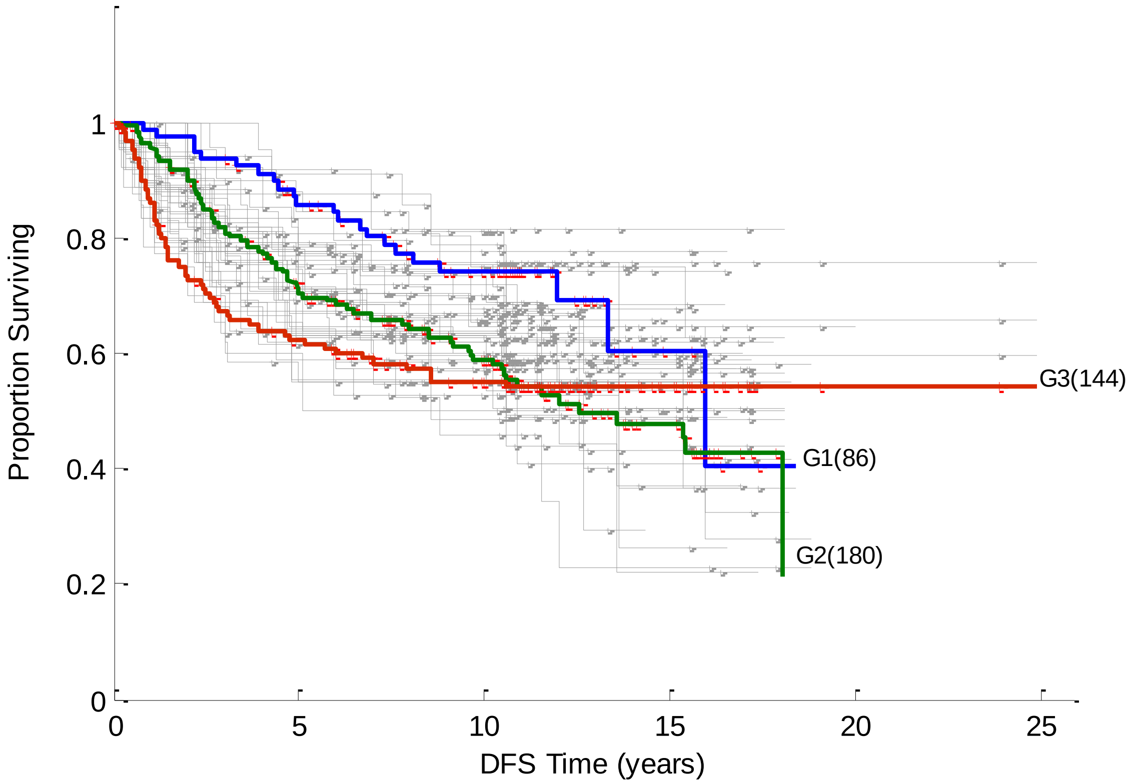

(B)

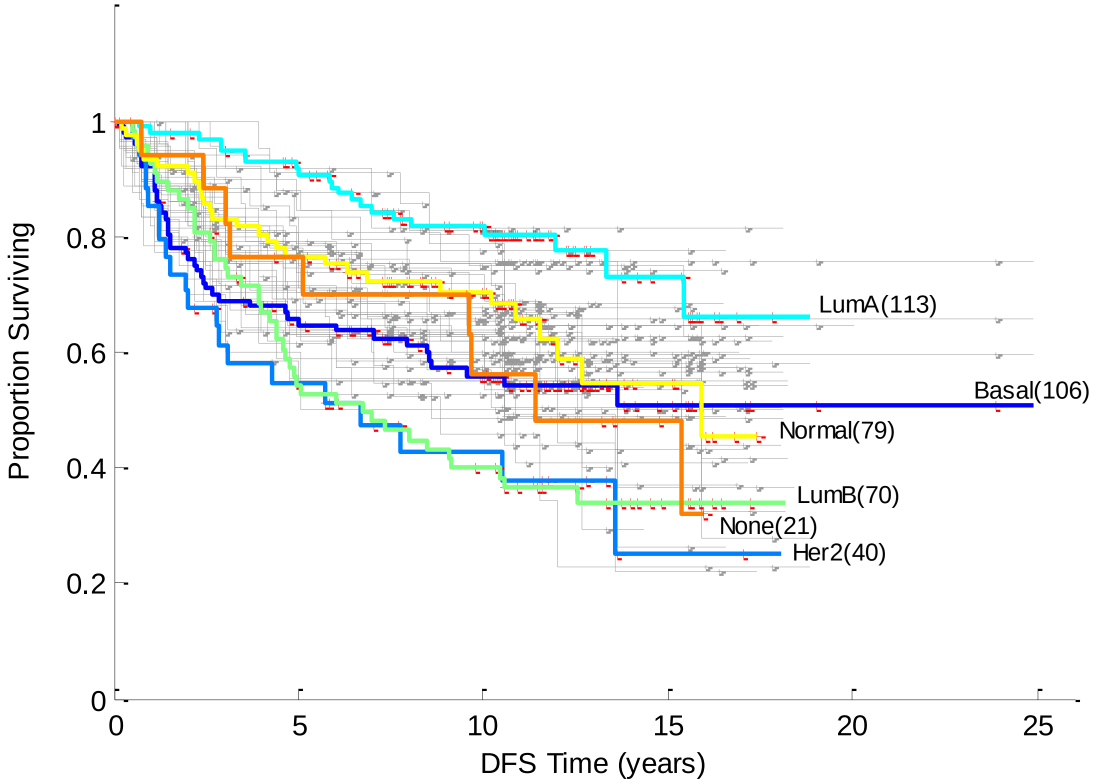

Supplementary Figure 3. Kaplan-Meier plots of pairs of biclusters showing differences in survival. Kaplan-Meier plots estimate disease free survival (DFS) distribution of pairwise biclusters comparison with significant difference in their survival distribution (logrank p value < 0.01). In each subplot, blue curve presents patients' group associated with good prognosis while curves in green represents patients' group associated with poor prognosis. Logrank p-values denotes on the top of each subplot indicates the significance of difference in survival distribution between two biclusters.

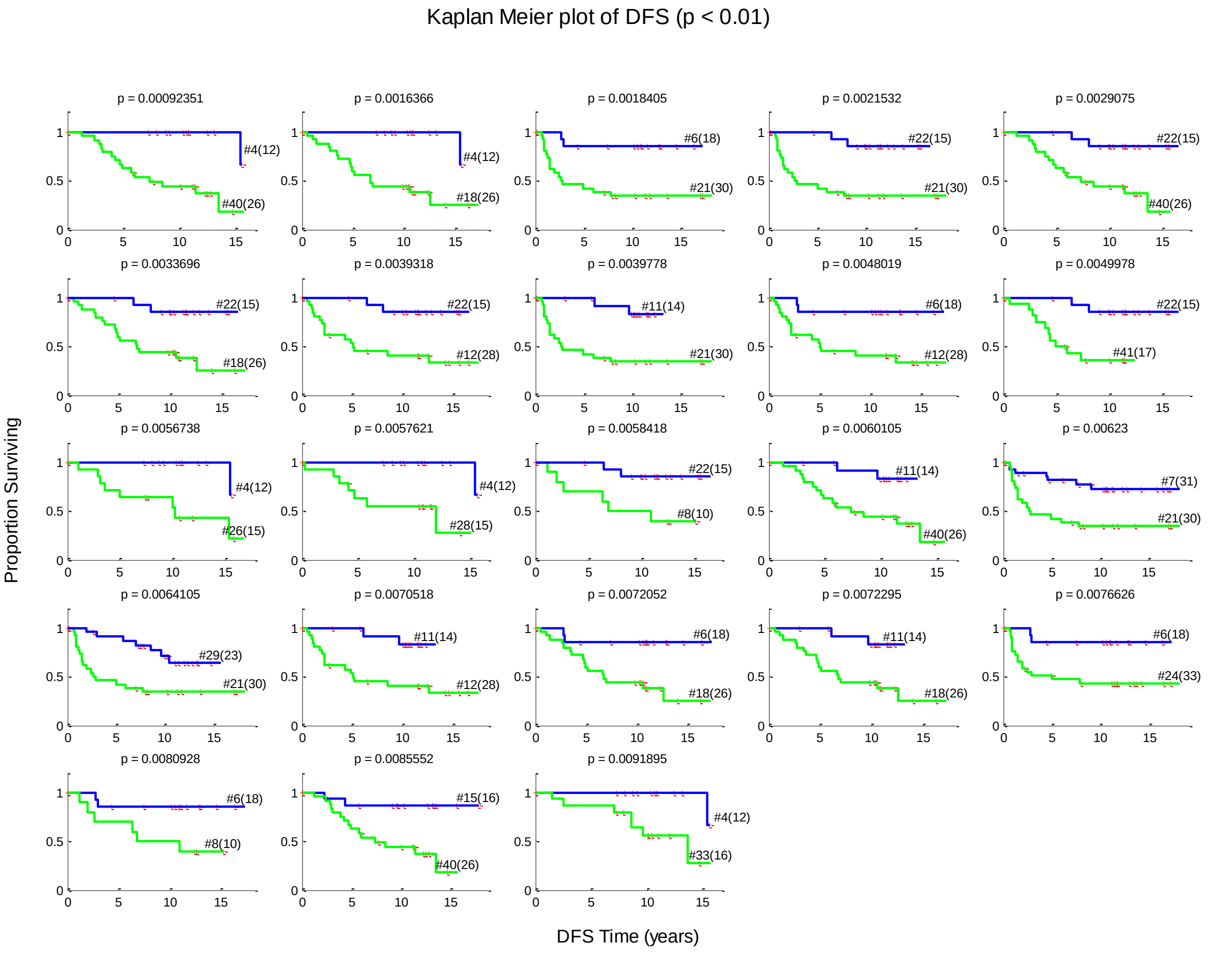

Supplementary Figure 4. Volcano plots showing genes that are differentially expressed between biclusters which have significant difference (logrank p values < 0.01) in their survival curves, associated with good and poor prognosis respectively. The statistical significance of differential gene expression was quantified by two sample t-test p-value. The fold change in expression level of each gene between two biclusters was evaluated. We identified genes whose expression level was significantly different by at least 2-fold change between biclusters associated with good and poor survival. We plot the statistical significance of differentially expressed genes against fold change in the expression levels between low and high risk biclusters to identify both statistically and biologically significant genes. By setting the cut off of significance level at 0.05 (horizontal red dashed line) and fold change at 2 (two vertical red dashed lines), the volcano plots identified genes with elevated expression levels, which represents the top-most significantly differentially expressed genes between biclusters associated with good and poor survival. The volcano plot was generated by *mavolcanoplot* in MatLab.

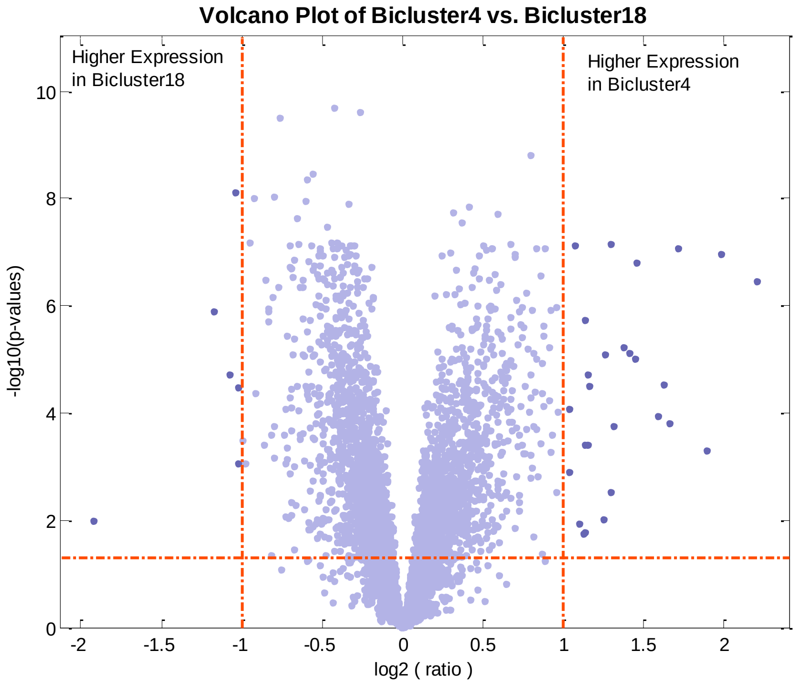

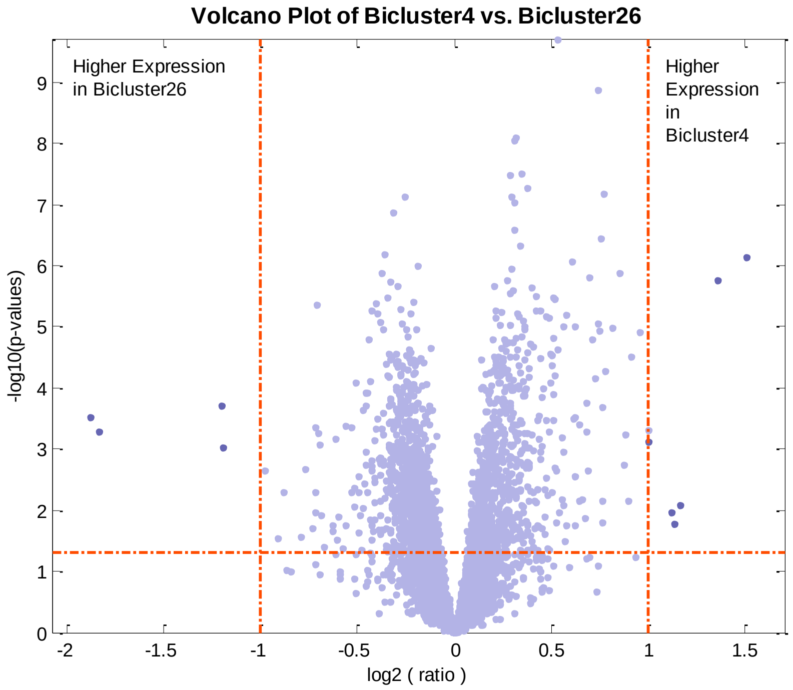

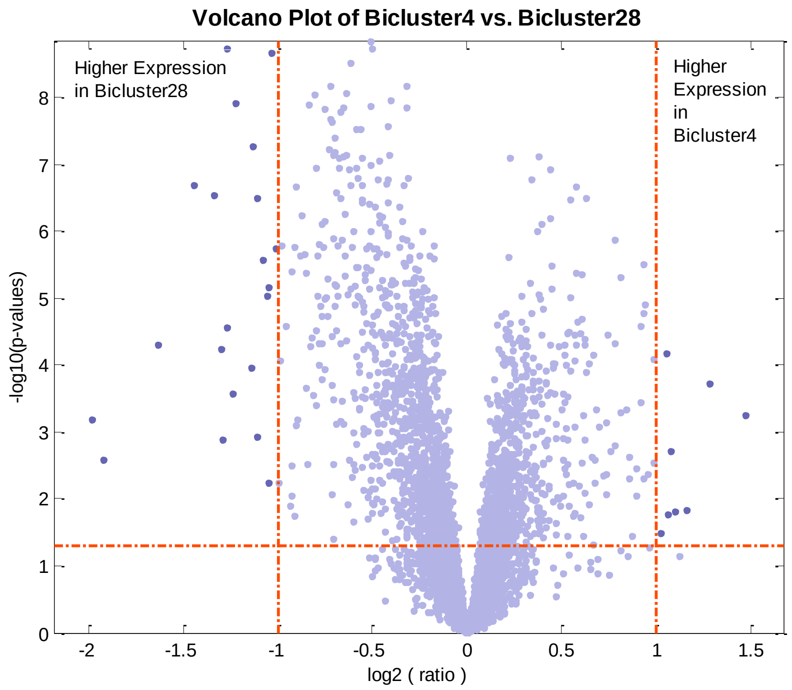
Volcano Plot of Bicluster4 vs. Bicluster28
Higher Expression in Bicluster28
Higher Expression in Bicluster4
-log10(p-values)
log2 (ratio)
0
1
2
3
4
5
6
7
8
-2
-1.5
-1
-0.5
0
0.5
1
1.5

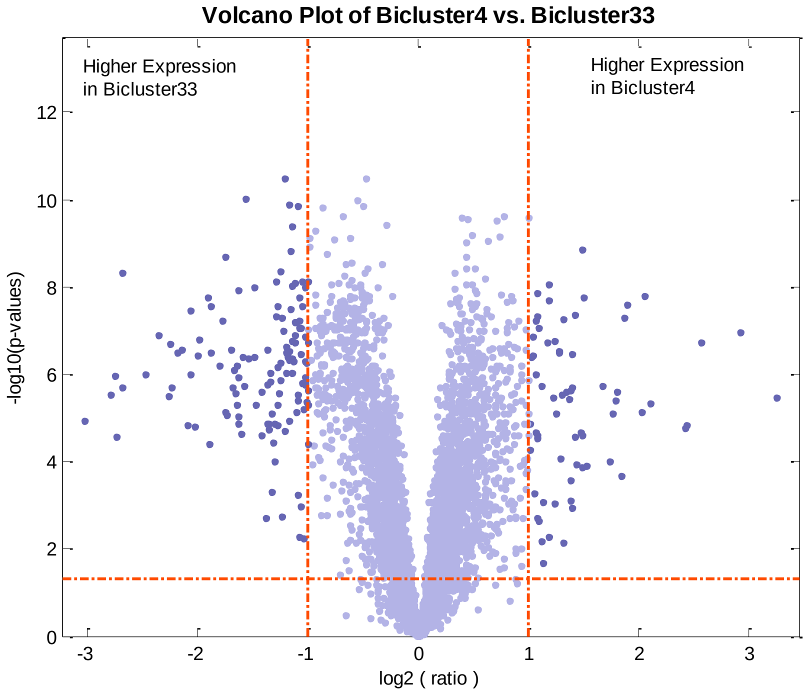
Volcano Plot of Bicluster4 vs. Bicluster33
Higher Expression in Bicluster33
Higher Expression in Bicluster4
-log10(p-values)
log2 (ratio)
0
2
4
6
8
10
12
-3
-2
-1
0
1
2
3

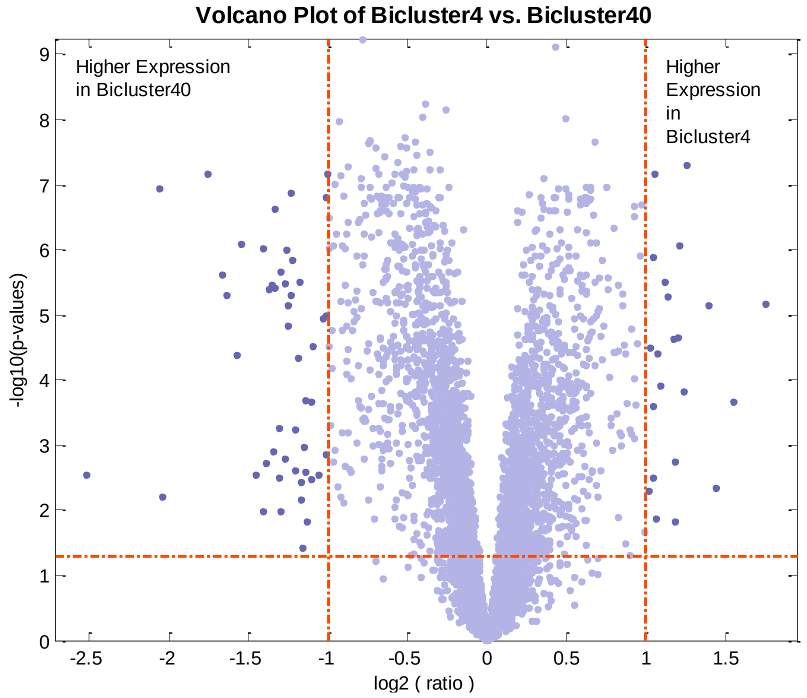
Volcano Plot of Bicluster4 vs. Bicluster40
Higher Expression in Bicluster40
Higher Expression in Bicluster4
-log10(p-values)
log2 (ratio)
0
1
2
3
4
5
6
7
8
9
-2.5
-2
-1.5
-1
-0.5
0
0.5
1
1.5

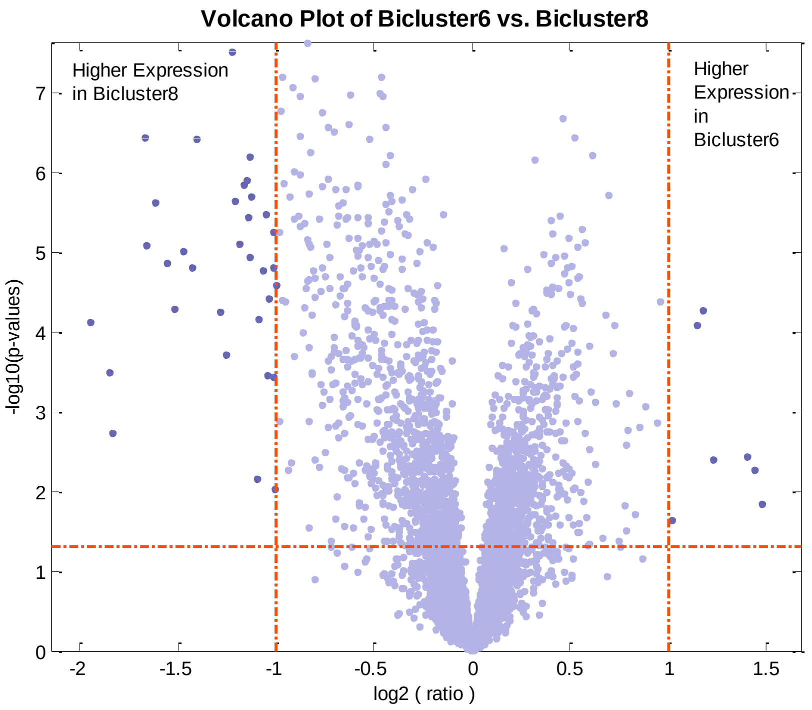
Volcano Plot of Bicluster6 vs. Bicluster8
Higher Expression in Bicluster8
Higher Expression in Bicluster6
-log10(p-values)
log2 (ratio)

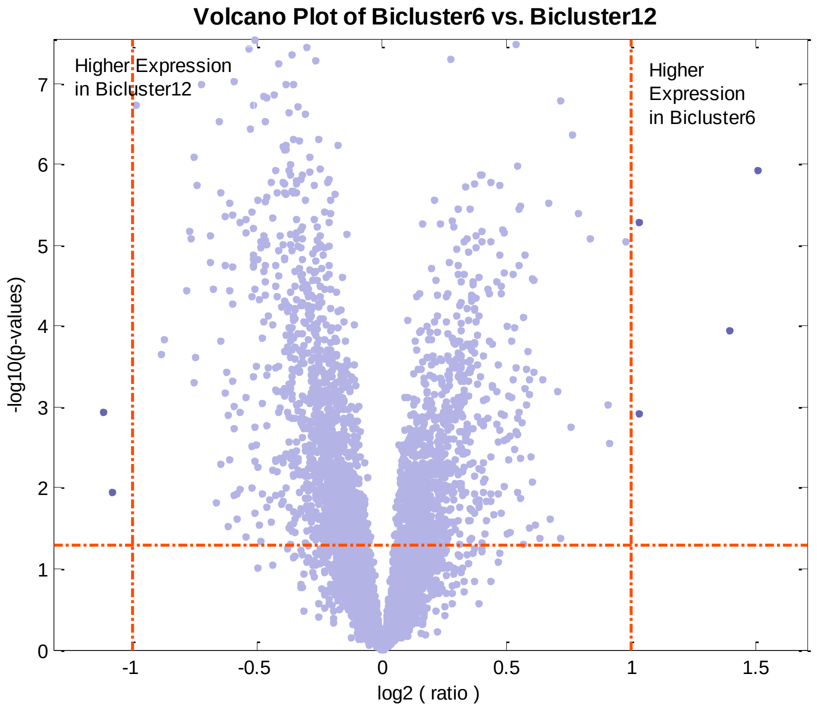
Volcano Plot of Bicluster6 vs. Bicluster12
Higher Expression in Bicluster12
Higher Expression in Bicluster6
-log10(p-values)
log2 (ratio)

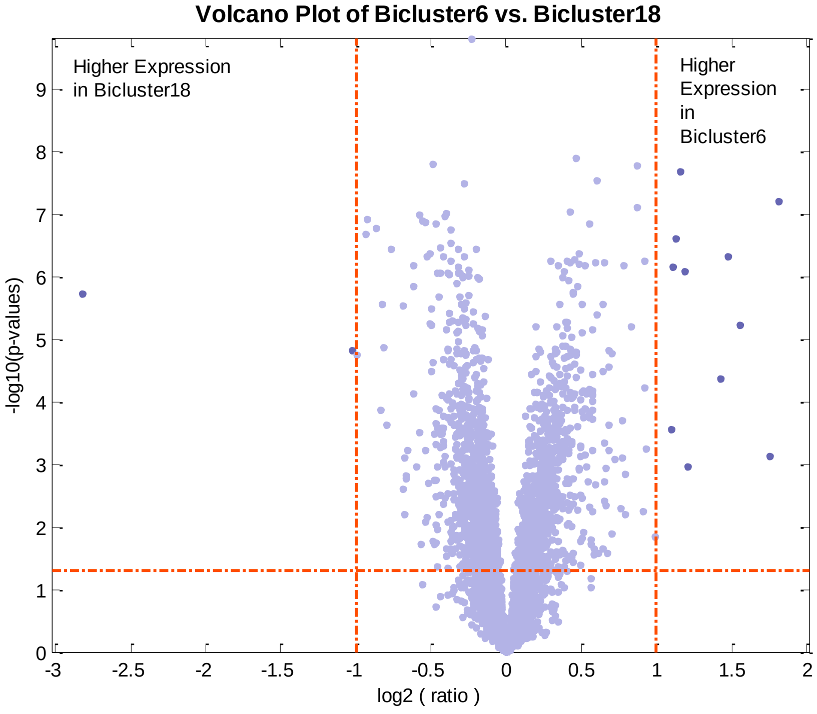
Volcano Plot of Bicluster6 vs. Bicluster18
Higher Expression in Bicluster18
Higher Expression in Bicluster6
-log10(p-values)
log2 (ratio)

Volcano Plot of Bicluster6 vs. Bicluster21

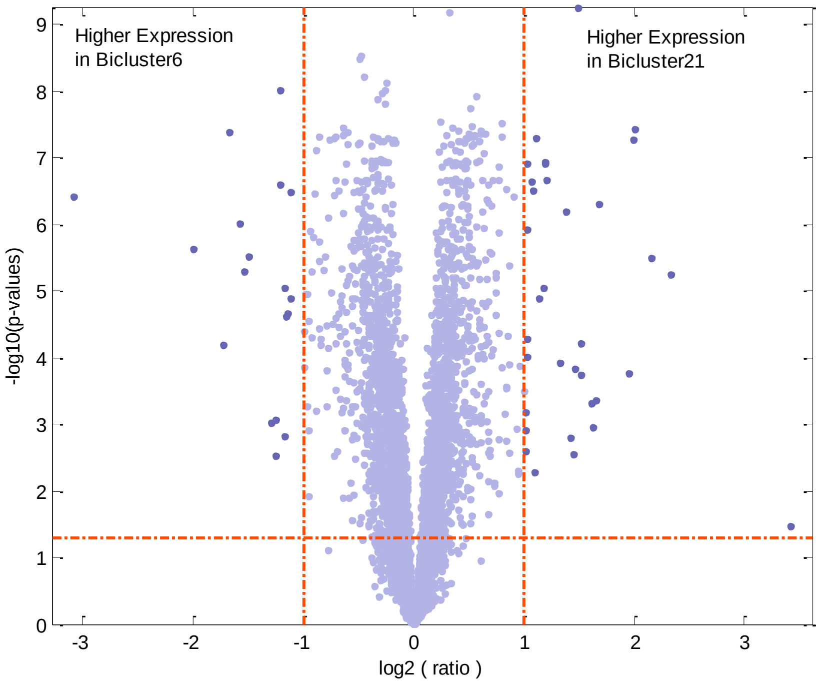

Volcano Plot of Bicluster6 vs. Bicluster24

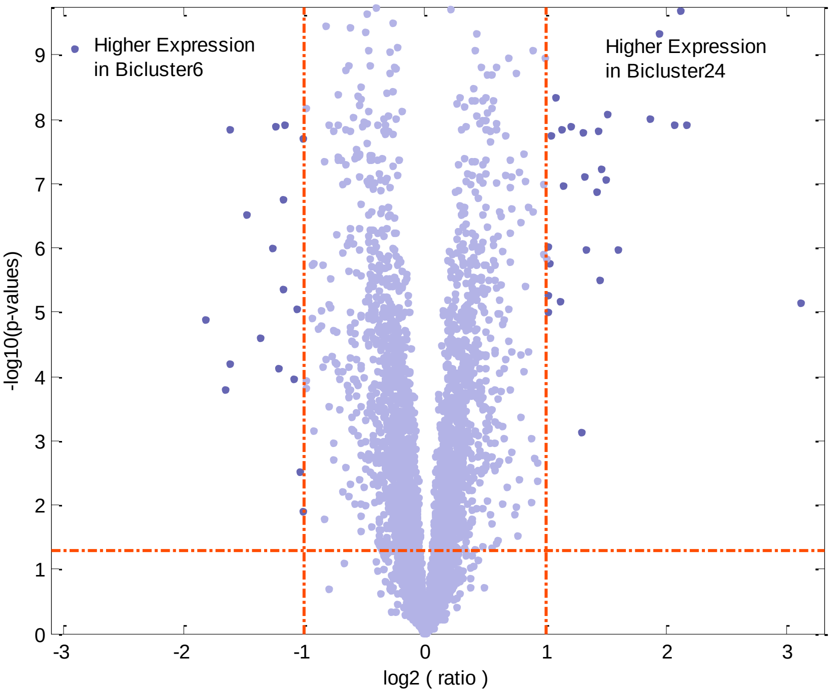

Volcano Plot of Bicluster7 vs. Bicluster21

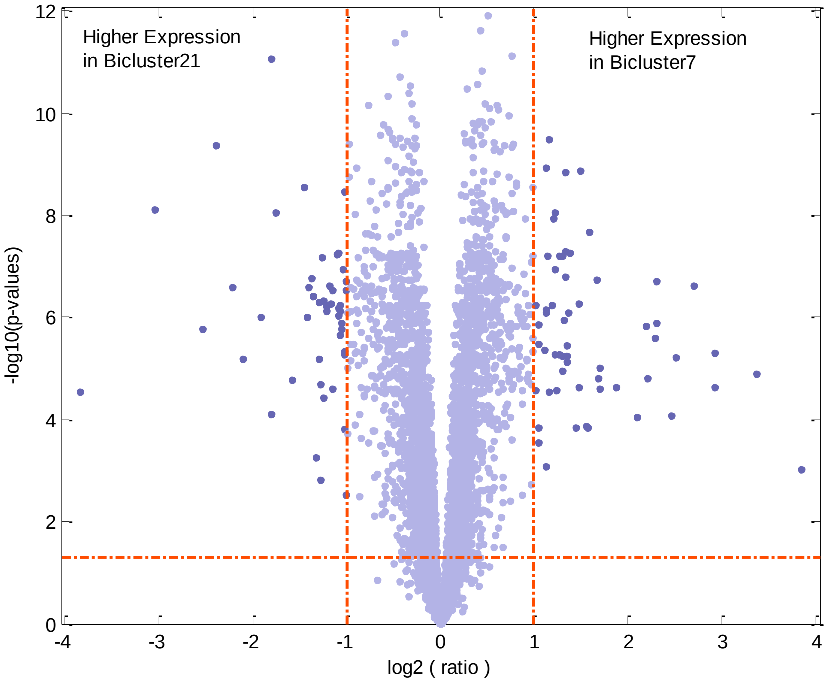

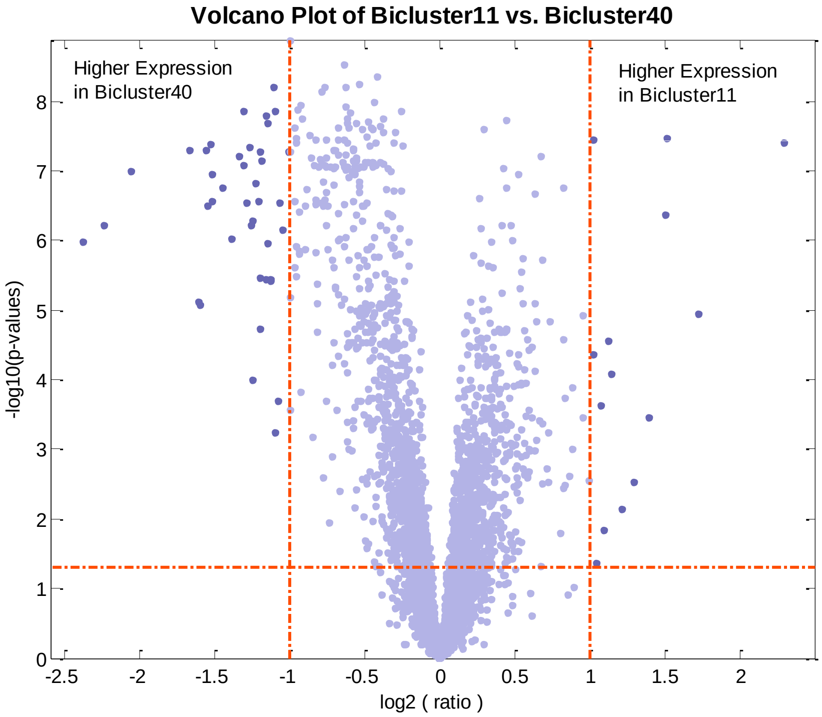
Volcano Plot of Bicluster11 vs. Bicluster40
Higher Expression in Bicluster40
Higher Expression in Bicluster11
-log10(p-values)
log2 (ratio)

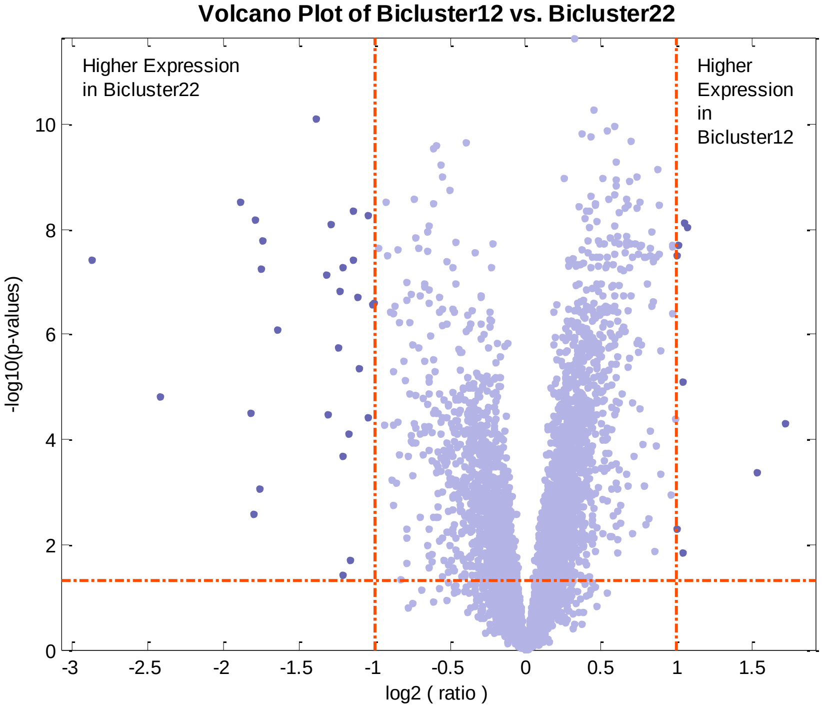
Volcano Plot of Bicluster12 vs. Bicluster22
Higher Expression in Bicluster22
Higher Expression in Bicluster12
-log10(p-values)
log2 (ratio)

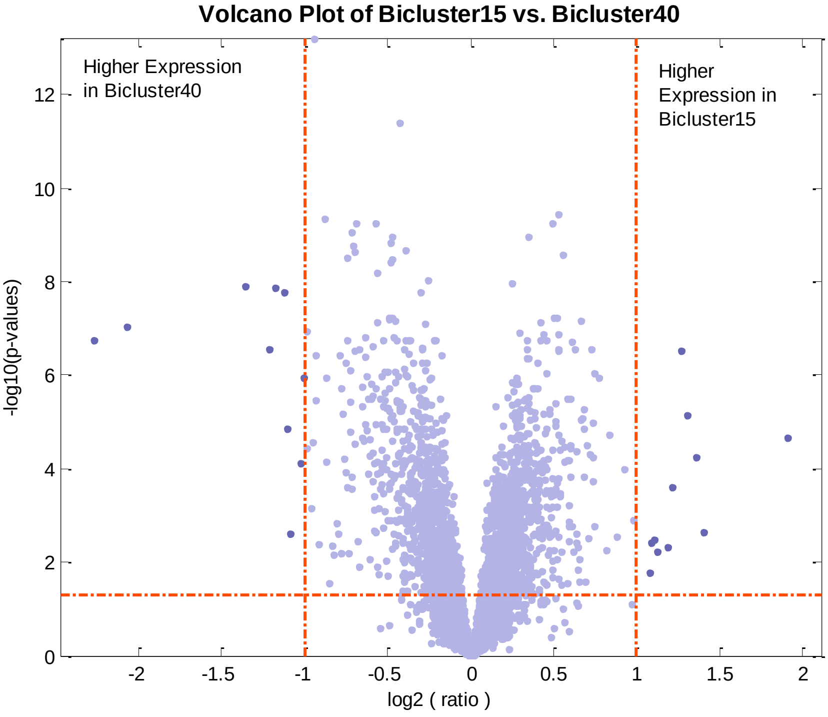
Volcano Plot of Bicluster15 vs. Bicluster40
Higher Expression in Bicluster40
Higher Expression in Bicluster15
-log10(p-values)
log2 (ratio)

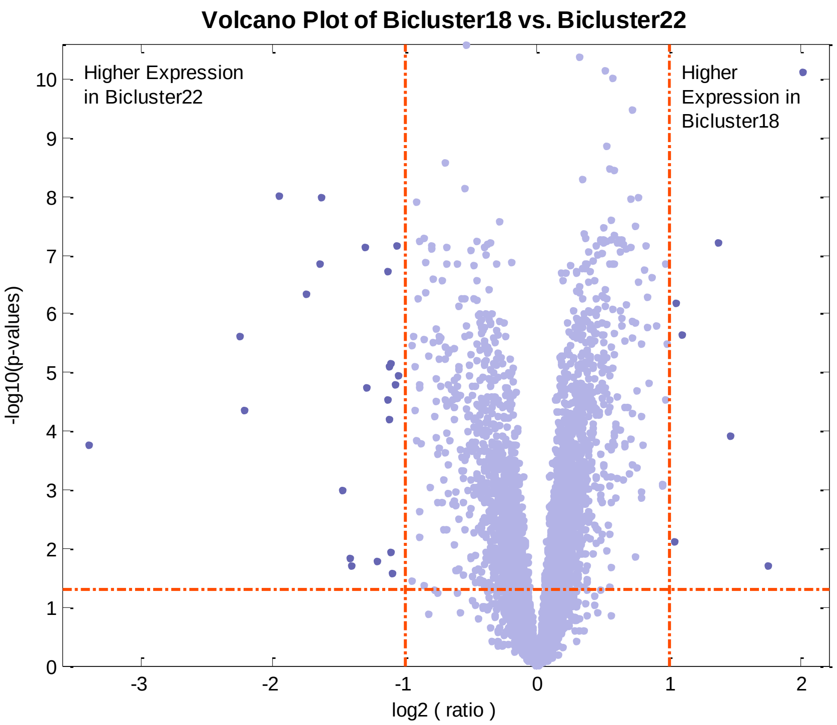
Volcano Plot of Bicluster18 vs. Bicluster22
Higher Expression in Bicluster22
Higher Expression in Bicluster18
-log10(p-values)
log2 (ratio)

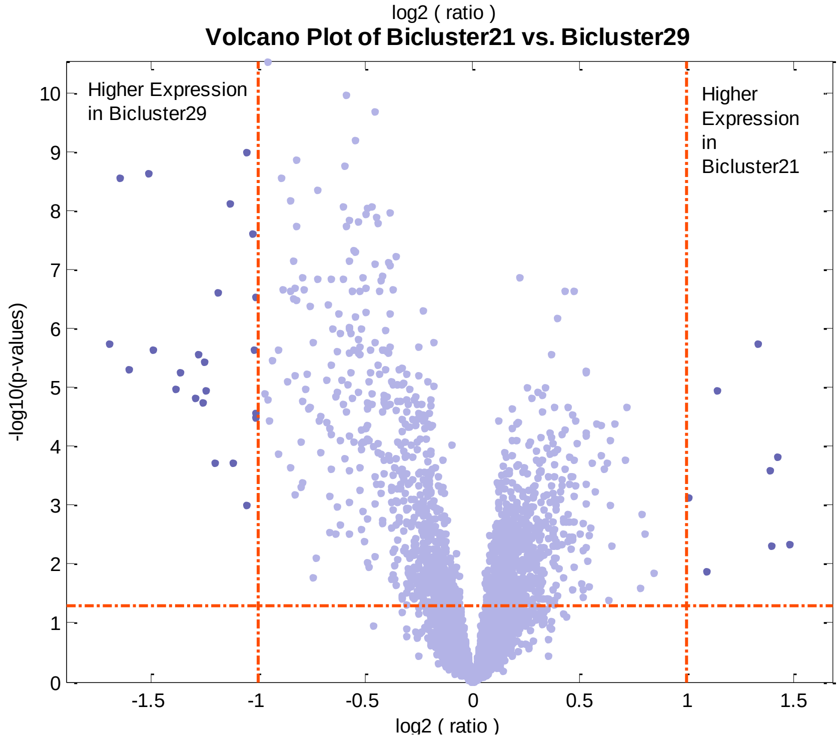
Volcano Plot of Bicluster21 vs. Bicluster29
Higher Expression in Bicluster29
Higher Expression in Bicluster21
-log10(p-values)
log2 (ratio)

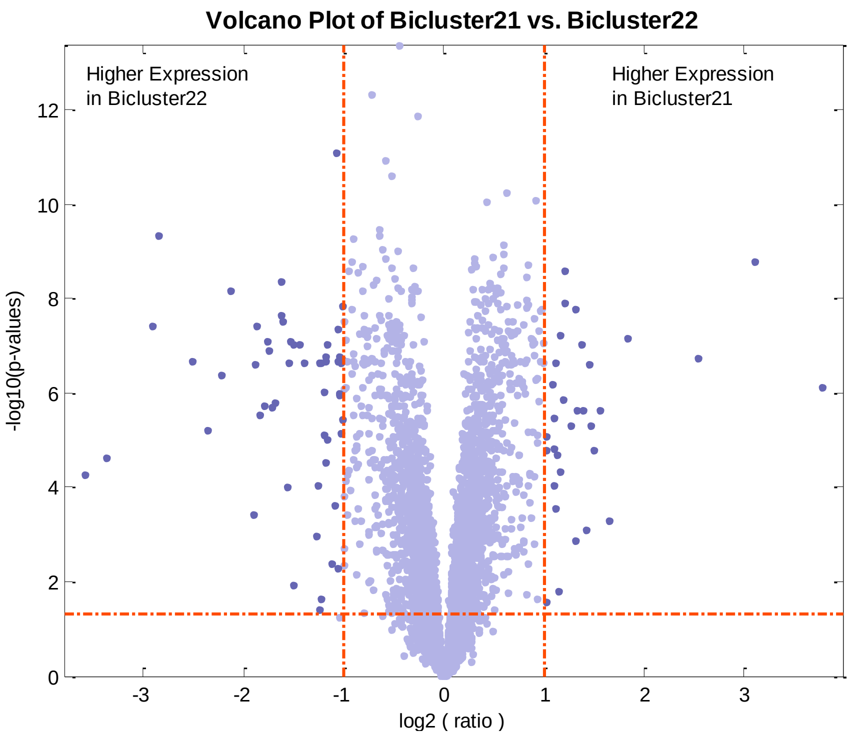
Volcano Plot of Bicluster21 vs. Bicluster22
Higher Expression in Bicluster22
Higher Expression in Bicluster21
-log10(p-values)
log2 (ratio)

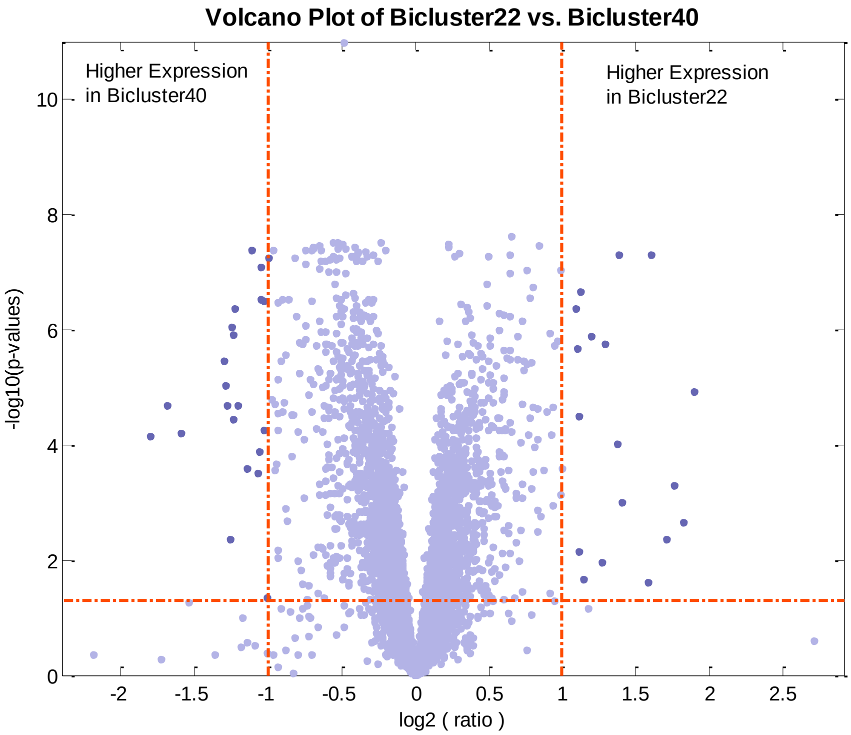
Volcano Plot of Bicluster22 vs. Bicluster40
Higher Expression
in Bicluster40
Higher Expression
in Bicluster22
-log10(p-values)
log2 (ratio)
0
2
4
6
8
10
-2
-1.5
-1
-0.5
0
0.5
1
1.5
2
2.5

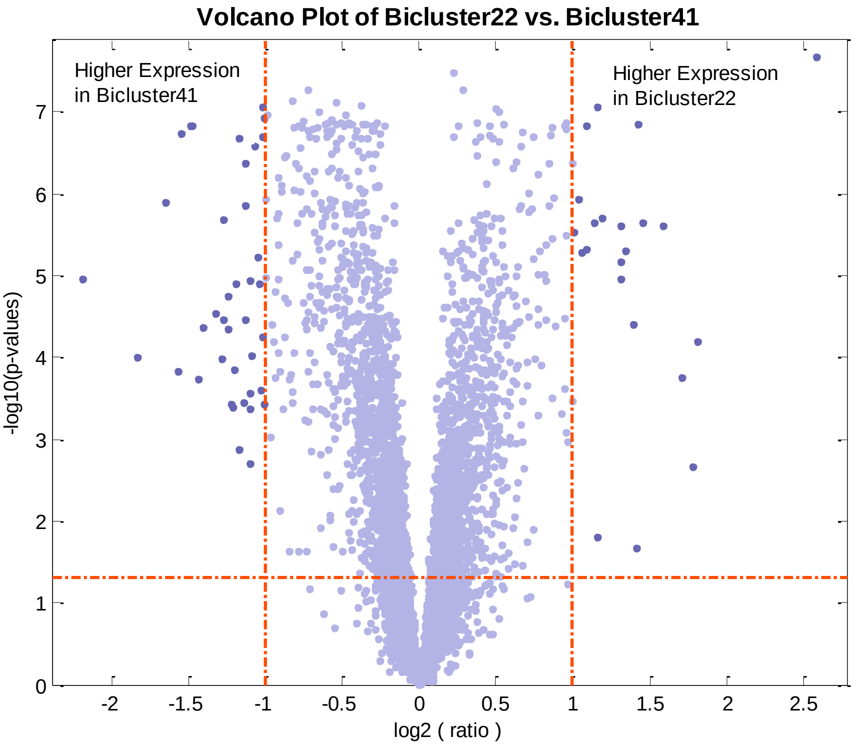
Volcano Plot of Bicluster22 vs. Bicluster41
Higher Expression
in Bicluster41
Higher Expression
in Bicluster22
-log10(p-values)
log2 (ratio)
0
1
2
3
4
5
6
7
-2
-1.5
-1
-0.5
0
0.5
1
1.5
2
2.5

Supplementary Figure 5. Functional enrichment heatmap for PAM gene classifiers of biclusters which have significant difference (logrank p values < 0.01) in their survival curves, associated with good and poor prognosis.

Bicluster4 vs. Bicluster18

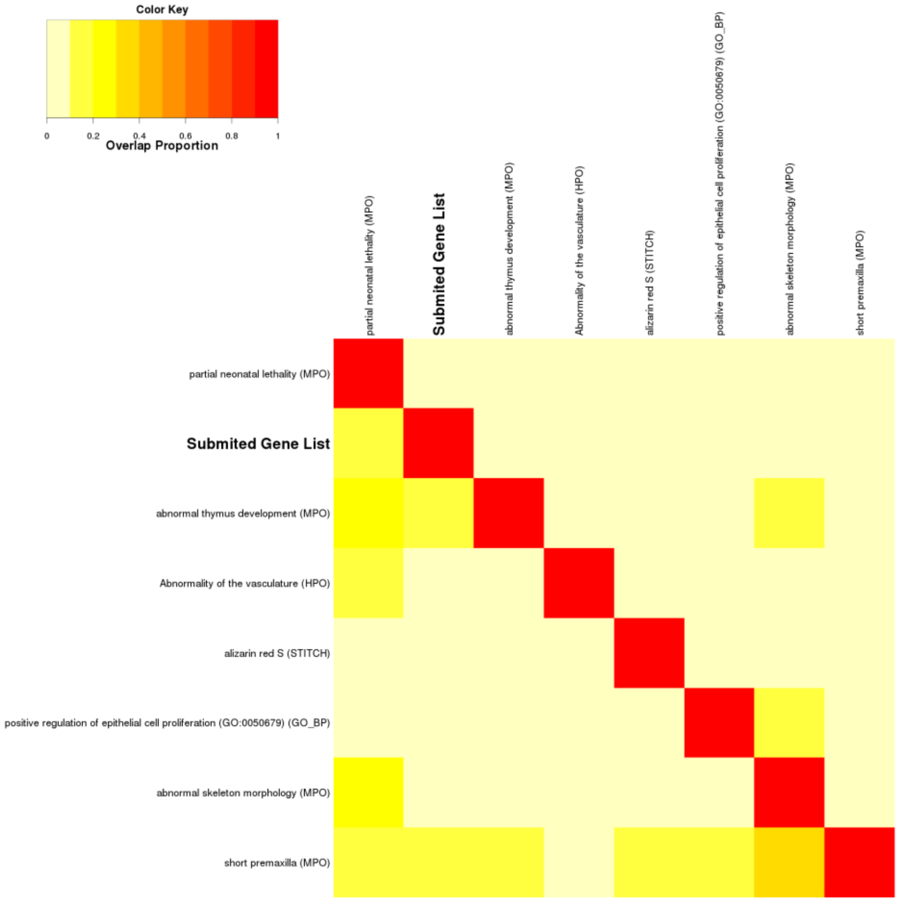

Bicluster4 vs. Bicluster26

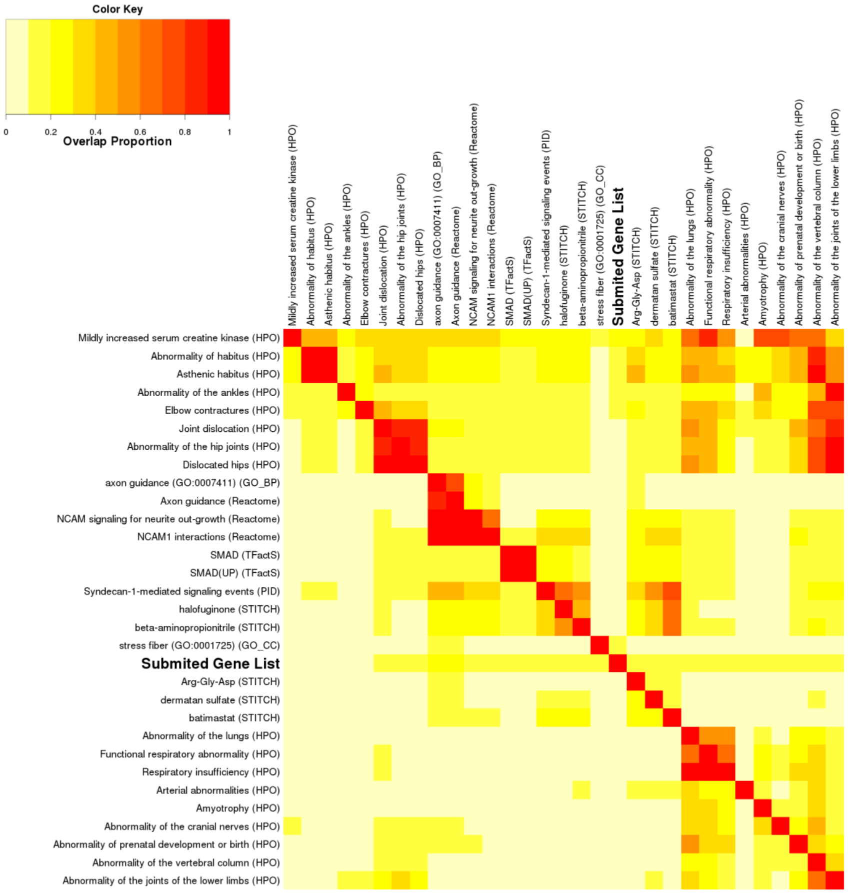

Bicluster4 vs. Bicluster33

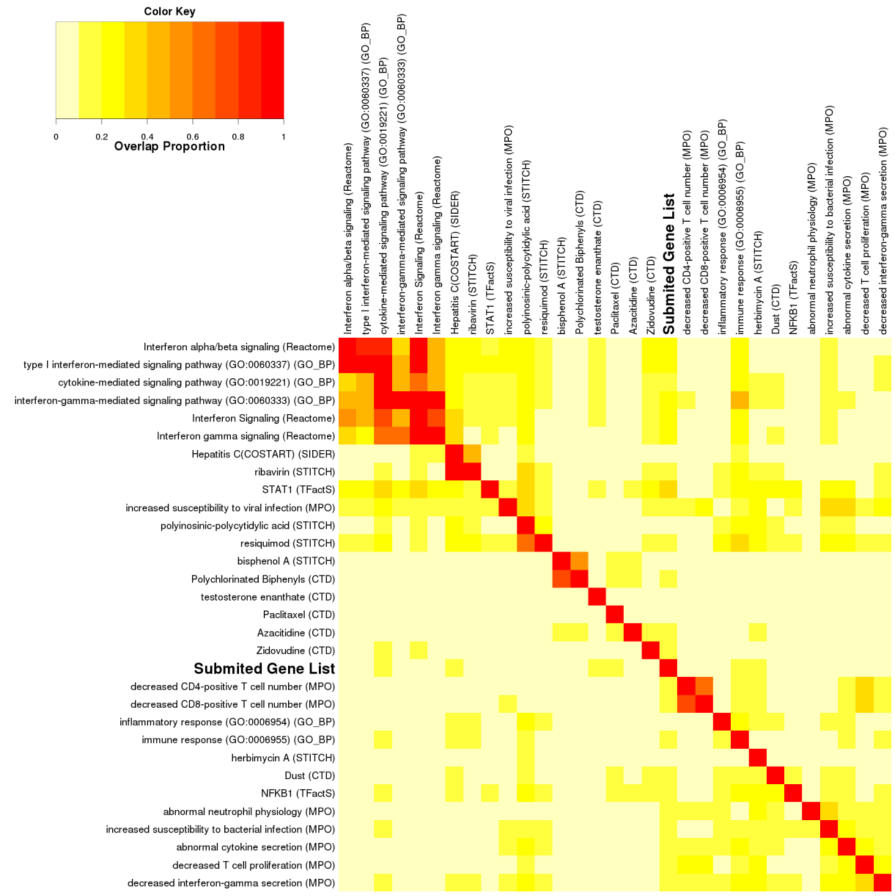

Bicluster6 vs. Bicluster8

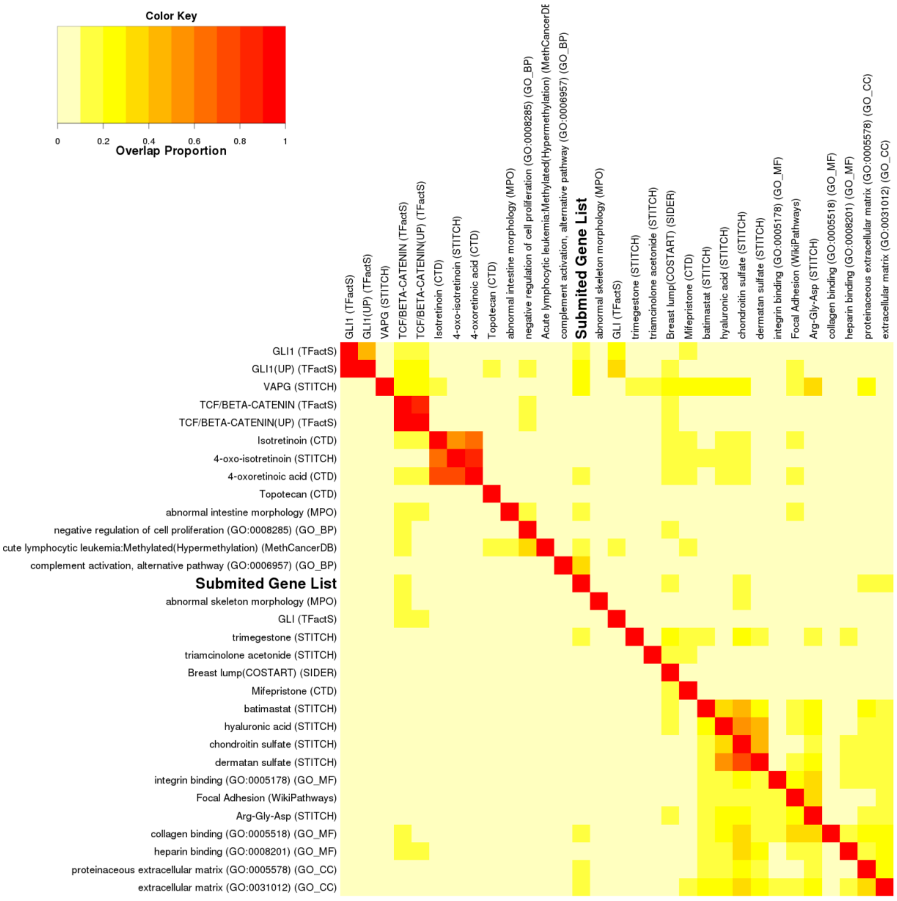

Bicluster6 vs. Bicluster12

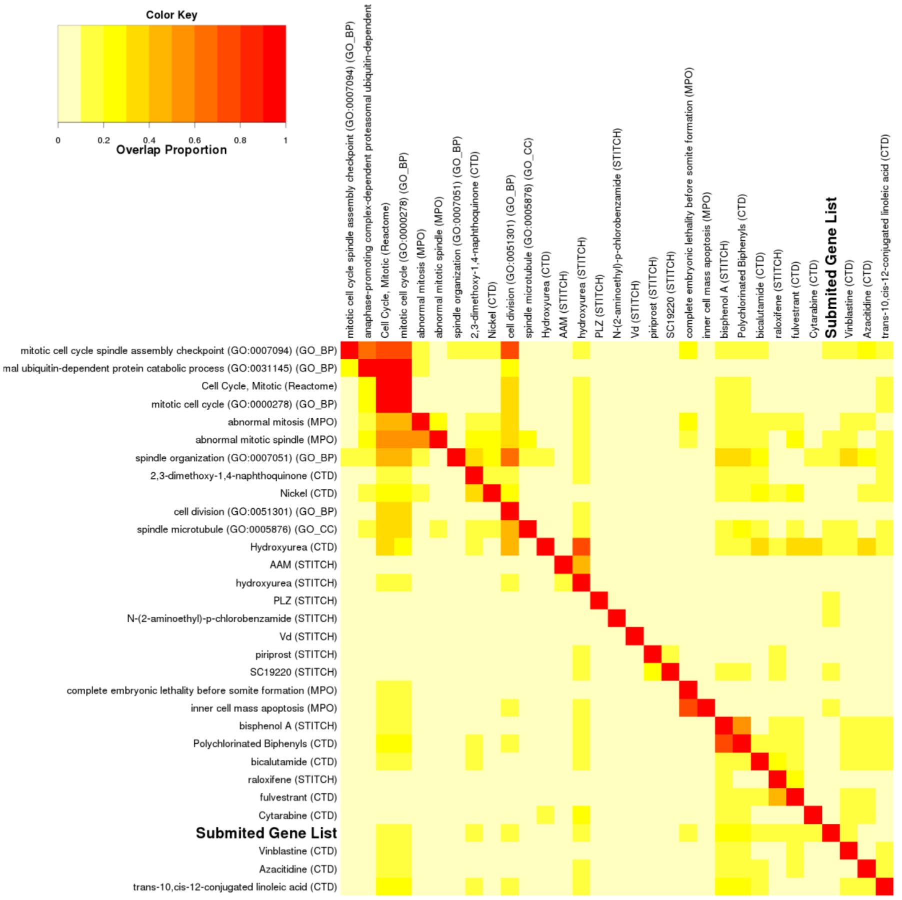

Bicluster6 vs. Bicluster21

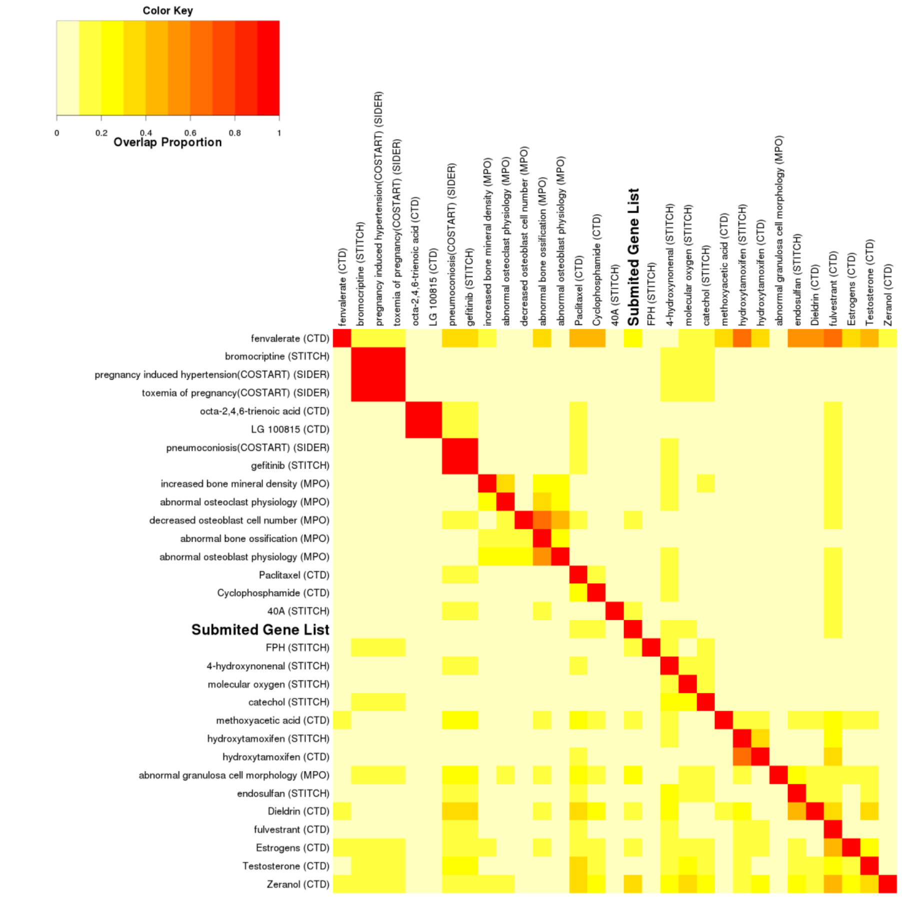

Bicluster6 vs. Bicluster24

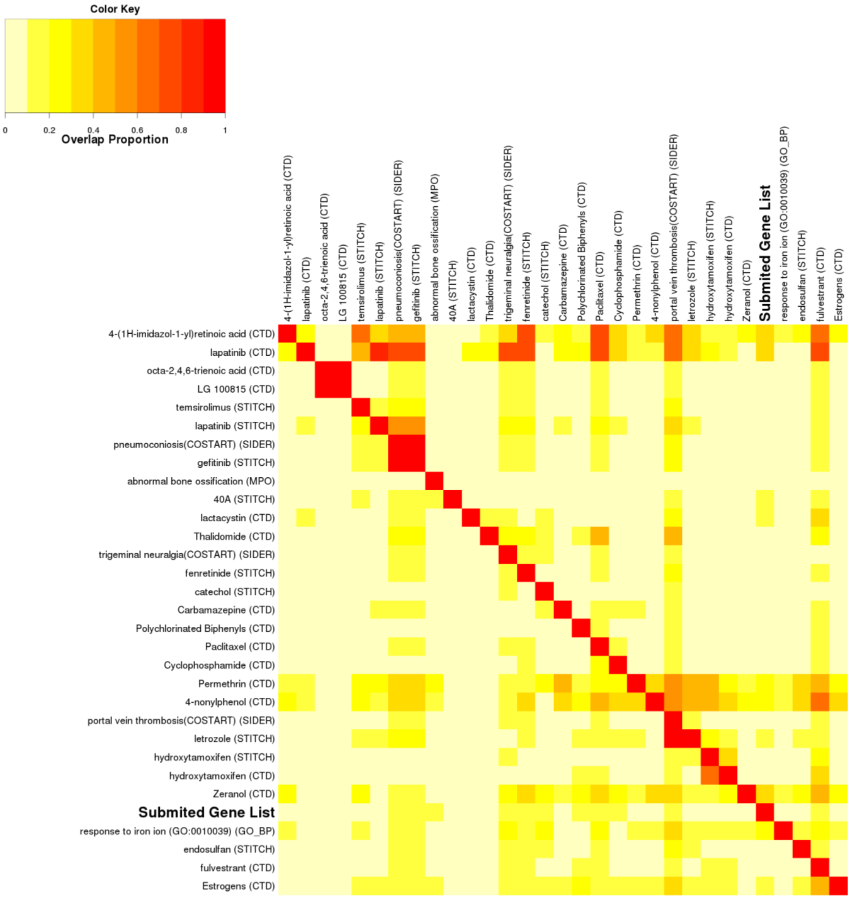

Bicluster7 vs. Bicluster21

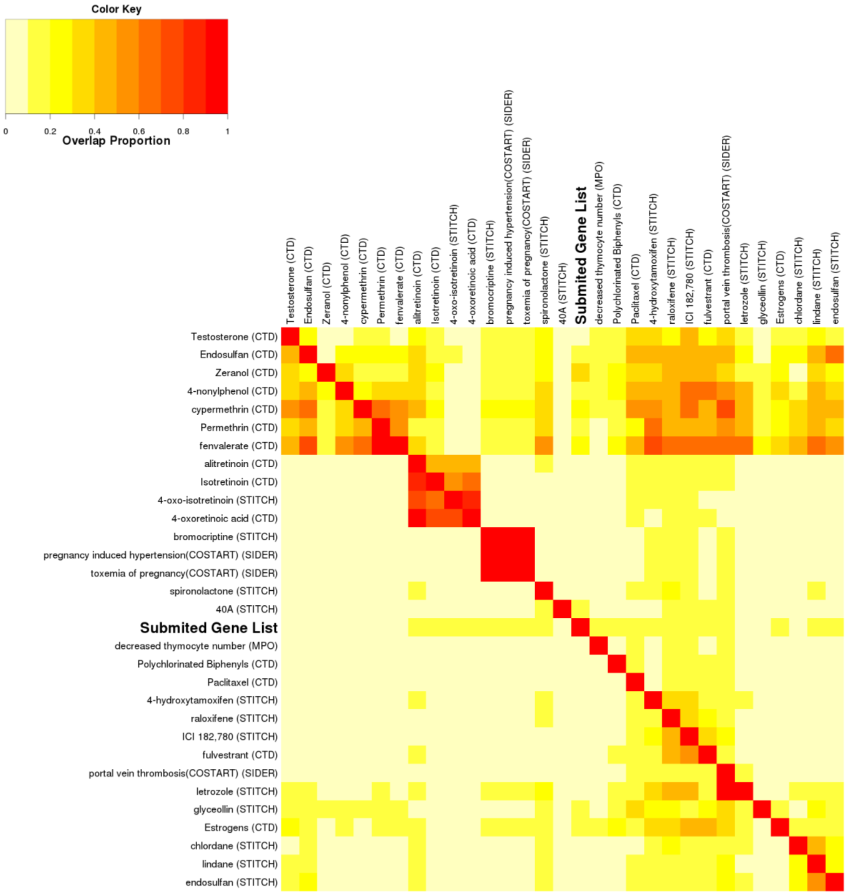

Bicluster11 vs. Bicluster12

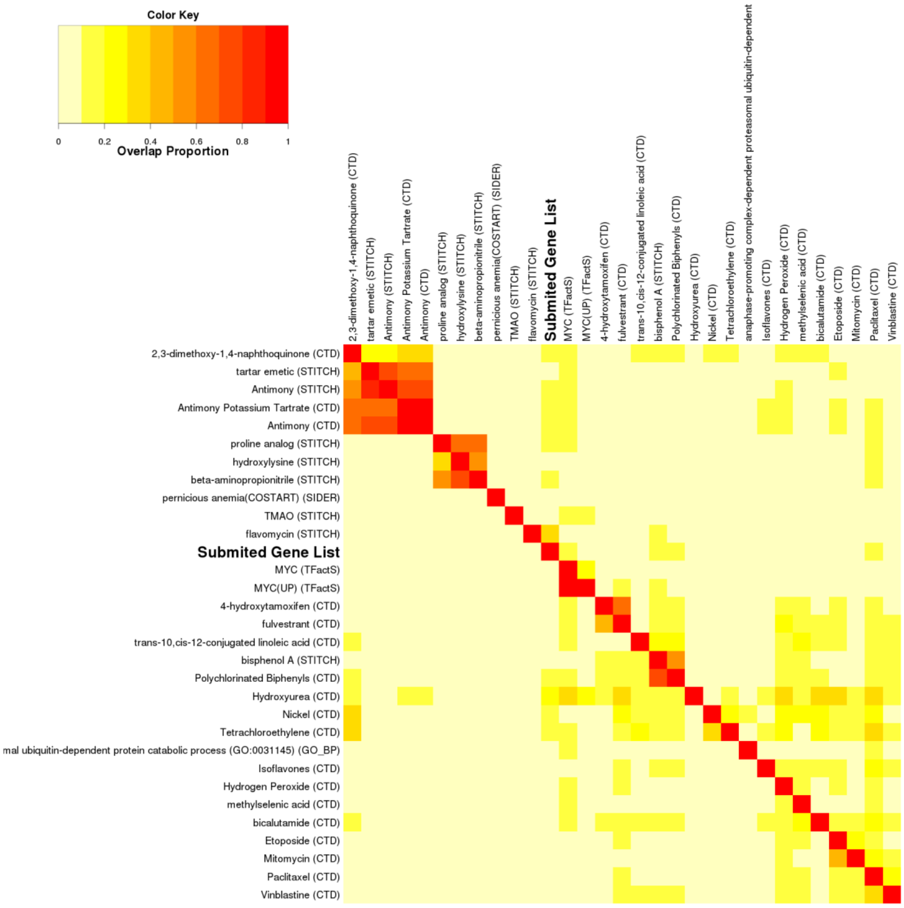

Bicluster11 vs. Bicluster18

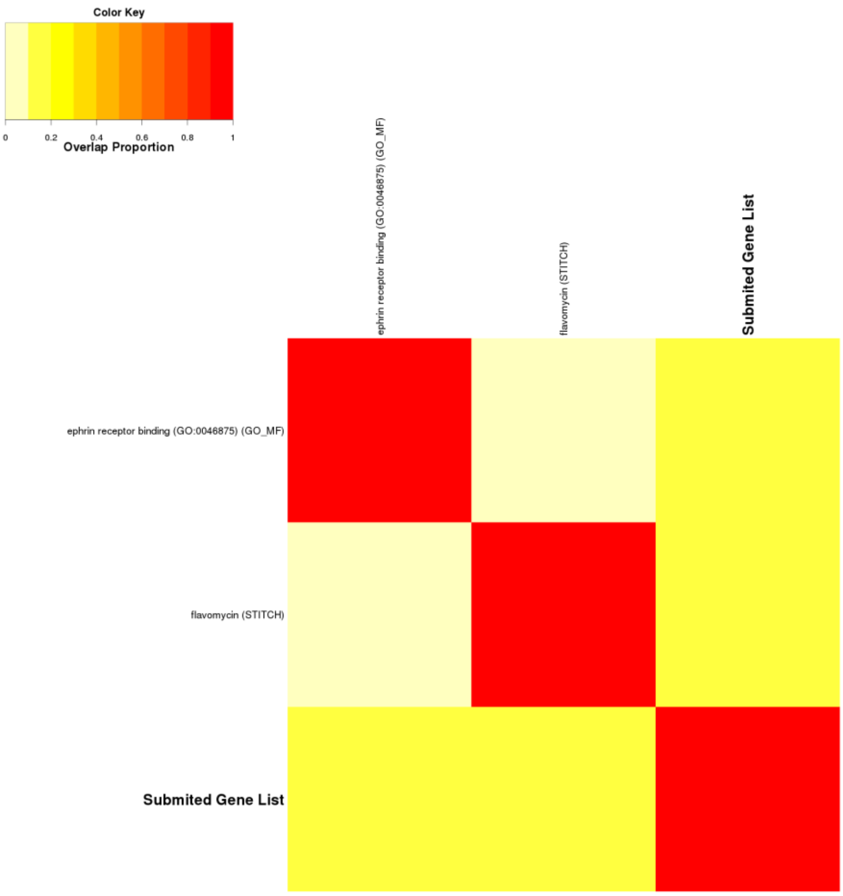

Bicluster11 vs. Bicluster21

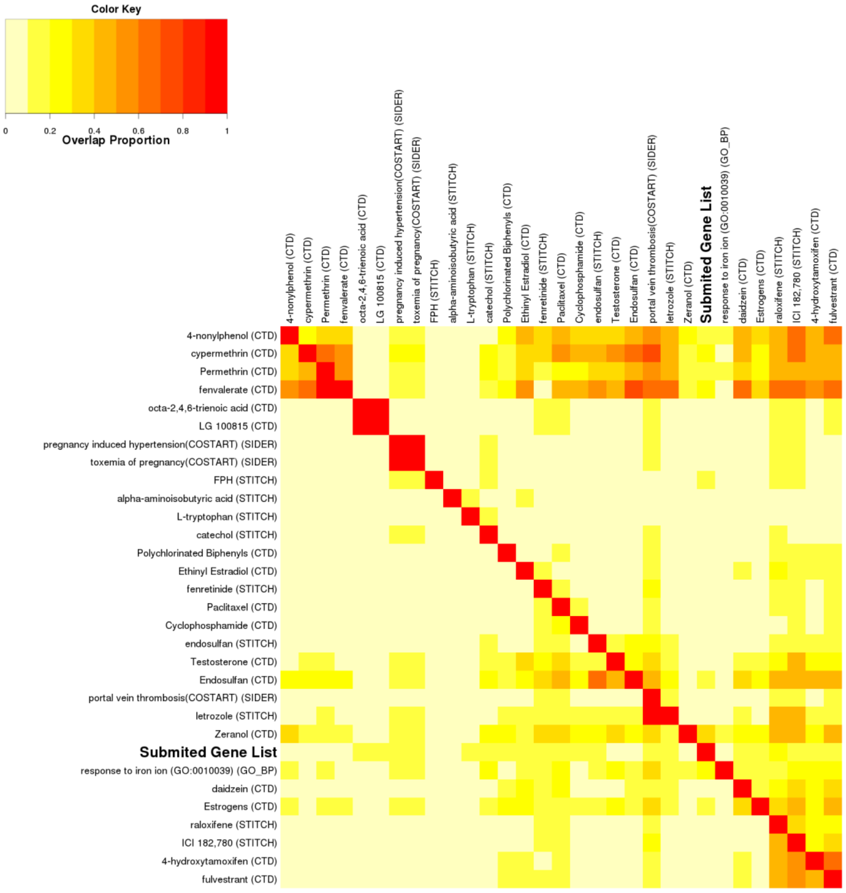

Bicluster11 vs. Bicluster40

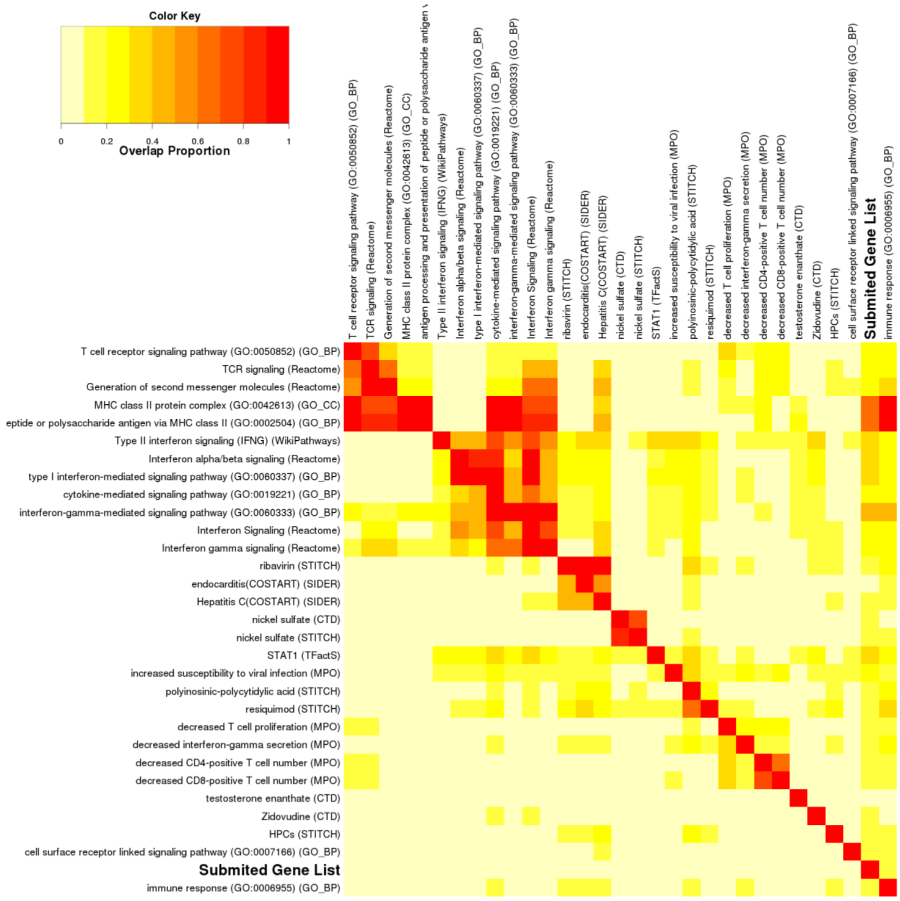

Bicluster15 vs. Bicluster40

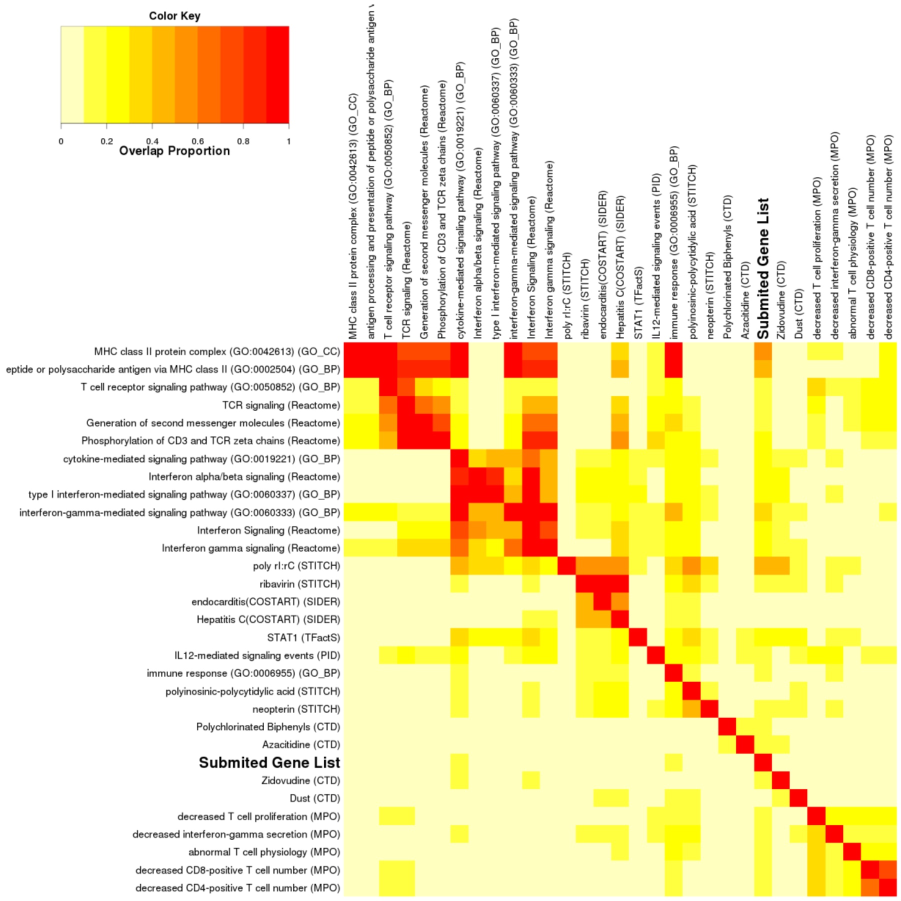

Bicluster22 vs. Bicluster8

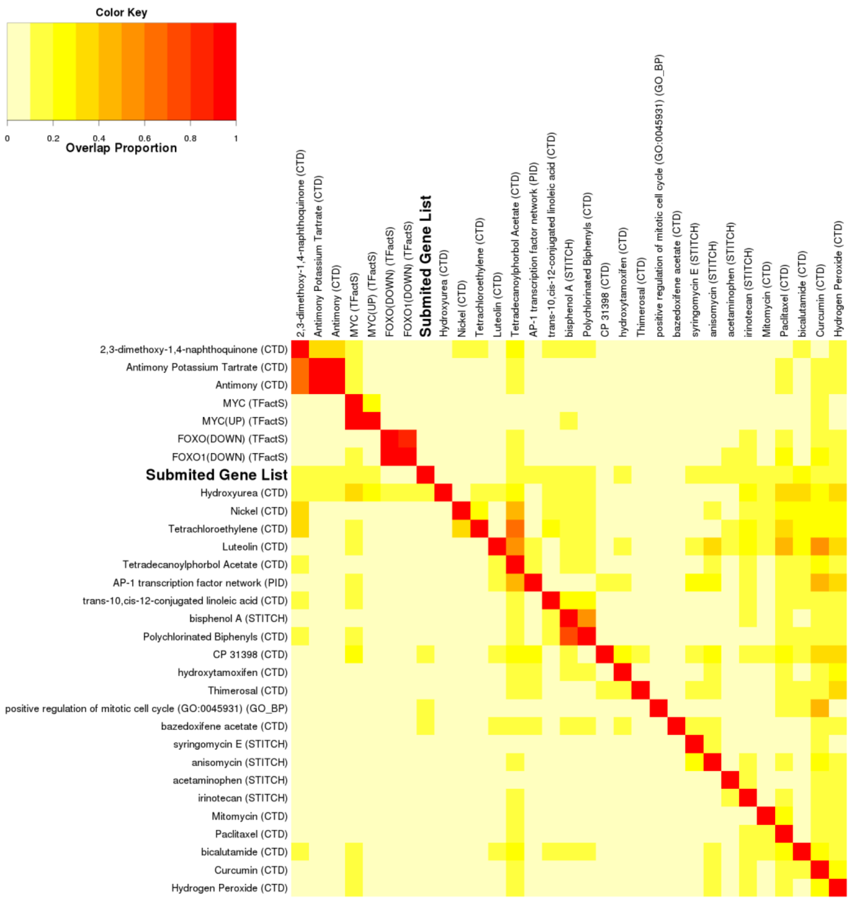

Bicluster22 vs. Bicluster21

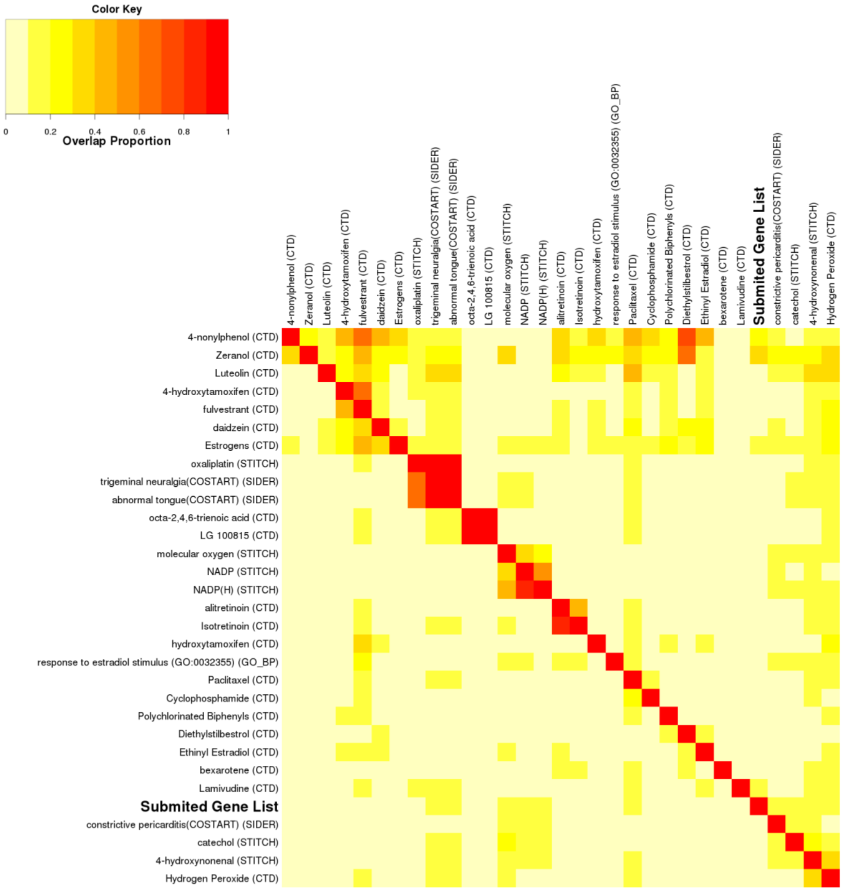

Bicluster22 vs. Bicluster40

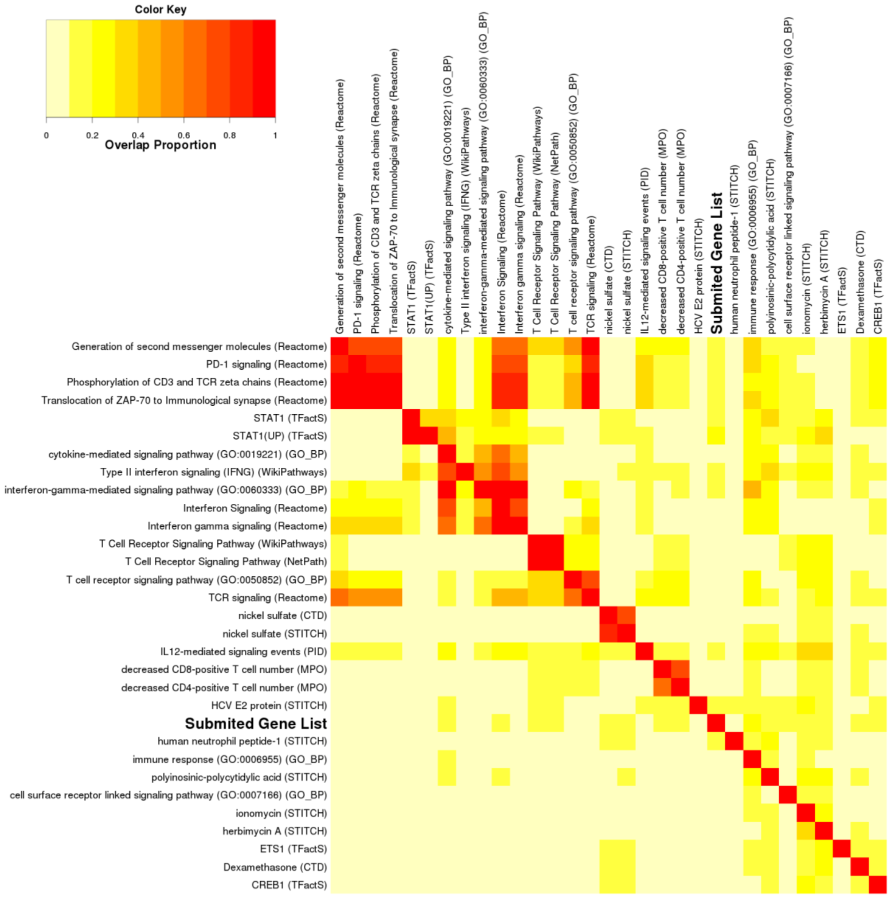

Bicluster22 vs. Bicluster41

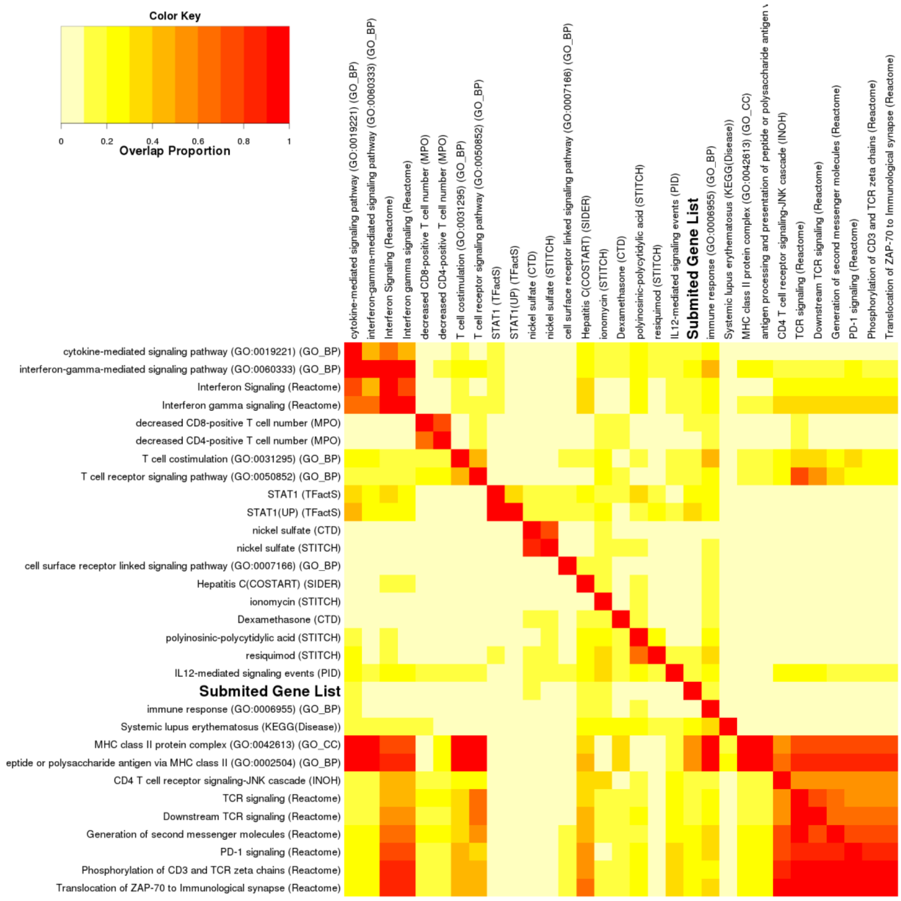

Supplement: Additional file 1: Figure S1 — Clinical information for each individual bicluster determined by cMonkey from gene expression data of breast cancer patients who had not received adjuvant treatment (437 patients). The clinical variables include cohort, lymph node status (LN.status: LN+, LN- and NA), estrogen receptor status (ER.status: ER+, ER- and NA), progesterone receptor status (PgR.status: PgR+, PgR- and NA), tumour grade (Grade: G1, G2, G3 and NA), molecular subtype (Subtype: Basal, Her2, Luminal A, Luminal B, Normal and None), tumour size (Size in mm) and patient Age (in years). Missing information is noted as NA (Not Available). The percentage noted on each bar indicates the percentage of tumour samples of the specific category in each bicluster. Figure S2. Kaplan-Meier estimates of survival distribution of randomly selected patient groups. Kaplan-Meier estimate of disease free survival (DFS) for 44 groups with arbitrary patient membership (all patients were randomly allocated into 44 groups), superimposed on survival curve of (A) histological grades and (B) tumour subtypes, respectively. Distribution of bicluster-associated survival curves in Figure 4 is much broader than the distribution of patient group-associated survival curves by chance. Figure S3. Kaplan-Meier plots of pairs of biclusters showing differences in survival. Kaplan-Meier plots estimate disease free survival (DFS) distribution of pairwise biclusters comparison with significant difference in their survival distribution (logrank p value < 0.01). In each subplot, blue curve presents patients’ group associated with good prognosis while curves in green represents patients’ group associated with poor prognosis. Logrank p-values denotes on the top of each subplot indicates the significance of difference in survival distribution between two biclusters. Figure S4. Volcano plots showing genes that are differentially expressed between biclusters which have significant difference (logrank p values < 0.01) in their survival curves, as [file 1471-2164-14-102-S1.pdf]
